# Pseudocyclic Arylbenziodoxaboroles as Water-Triggered Aryne Precursors in Reactions with Organic Sulfides

**DOI:** 10.1021/acs.orglett.4c00197

**Published:** 2024-02-26

**Authors:** Akira Yoshimura, Kim Ngo, Irina A. Mironova, Zachary S. Gardner, Gregory T. Rohde, Nami Ogura, Akiharu Ueki, Mekhman S. Yusubov, Akio Saito, Viktor V. Zhdankin

**Affiliations:** † Faculty of Pharmaceutical Sciences, Aomori University, 2-3-1 Kobata, Aomori 030-0943, Japan; ‡ Department of Chemistry and Biochemistry, University of Minnesota Duluth, Duluth, Minnesota 55812, United States; § Research School of Chemistry and Applied Biomedical Sciences, Tomsk Polytechnic University, Lenina av., 30, 634050 Tomsk, Russia; ∥ Marshall School, Duluth, Minnesota 55811, United States; ⊥ Division of Applied Chemistry, Institute of Engineering, Tokyo University of Agriculture and Technology, 2-24-16 Naka-cho, Koganei, Tokyo 184-8588, Japan

## Abstract

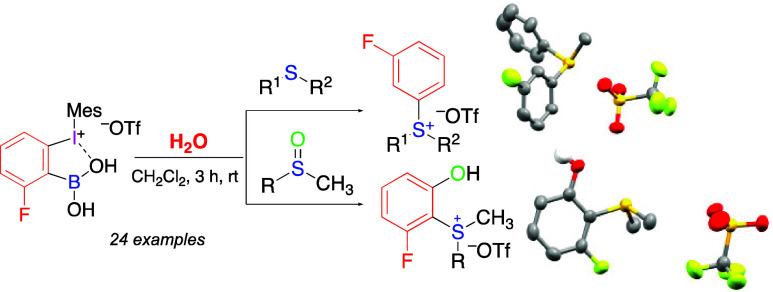

Pseudocyclic arylbenziodoxaboroles are unique aryne precursors under neutral aqueous conditions that selectively react with organic sulfides, forming the corresponding sulfonium salts. This reaction is compatible with various substituents (alkyl, halogen, CN, NO_2_, CHO, and cyclopropyl) in the aromatic ring or alkyl group of the sulfide. Similar reactions of sulfoxides afford *o*-hydroxy-substituted sulfonium salts. The structures of key products were confirmed by X-ray analysis.

Benzyne and other aryne intermediates have found broad application in modern organic synthesis.^1^ Common benzyne precursors require activation via application of heat, irradiation, a strong base (NaNH_2_, BuLi, RMgBr, LiHMDS, etc.), or the anhydrous fluoride anion, which is also a strong base and requires special handling to avoid moisture. For example, the most frequently employed Kobayashi aryne precursors, *o*-silylaryl triflates, are usually activated by being treated with anhydrous CsF under absolutely dry conditions.^1b^ The need for strongly basic, anhydrous conditions restricts the applicability of common benzyne precursors in some reactions. In particular, the synthetically important reactions of arynes **A** with organic sulfides under anhydrous basic conditions proceed via zwitterionic intermediates **B**, generally leading to various products of cyclization, rearrangement, or elimination but not to sulfonium salts (Scheme 1a).^1d,1h^

**Scheme 1:**
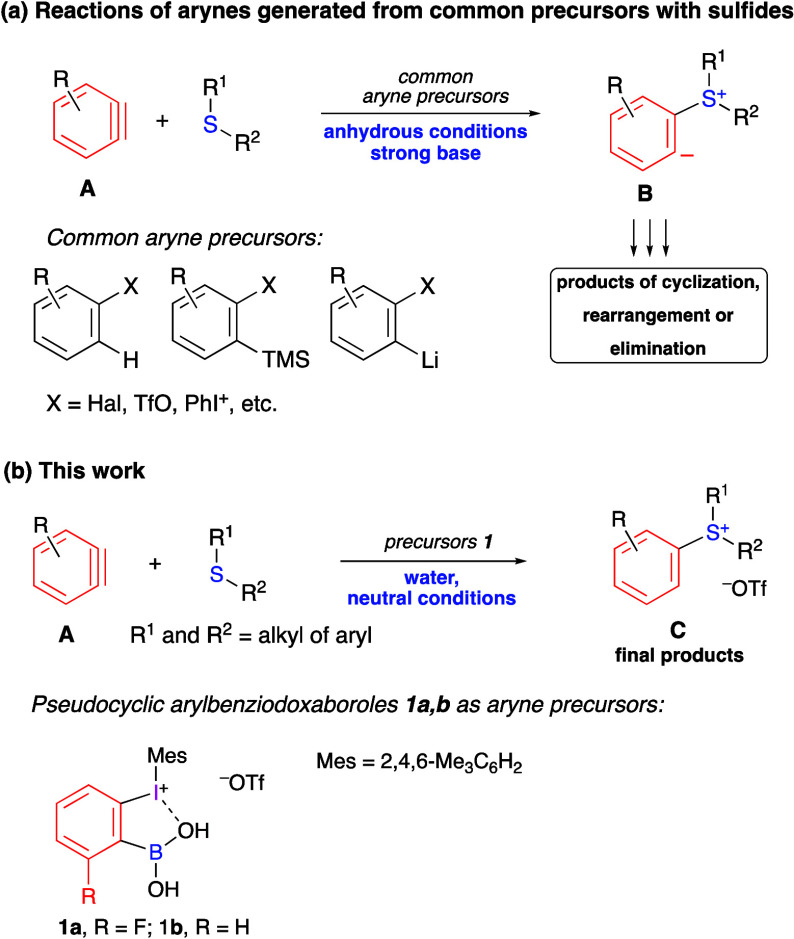
Reactions of Aryne Intermediates with Organic Sulfides

Pseudocyclic arylbenziodoxaboroles **1** are unique benzyne precursors that can be triggered under neutral or slightly acidic conditions in aqueous solutions at room temperature.^2^ We expected that in the reactions of arynes **A** with organic sulfides in the presence of water, initial zwitterionic intermediates **B** would be immediately protonated, resulting in the selective formation of various sulfonium salts **C** (Scheme 1b). It should be noted that sulfonium salts are important compounds with a wide range of applications, e.g., as acid generators in photolithography, photoinitiators in cationic polymerization, radical precursors, and cross-coupling partners in organic synthesis.^3^ In this Letter, we report reactions of benzyne precursors **1** with organic sulfides triggered by water in an organic solvent at room temperature that lead to the selective formation of various sulfonium salts.

Initially, we investigated reactions of reagent **1a** with thioanisole **2a** in various solvents at room temperature (Table 1). No product was formed when a solution of reagent **1a** and sulfide **2** was stirred in dry CH_2_Cl_2_ for 3 h at room temperature (Table 1, entry 1). However, the addition of water to the reaction mixture resulted in a dramatic change, with the most effective organic solvent:water ratio being 9:1 (v/v). The reaction of reagent **1a** with a small excess of thioanisole **2a** in a 9:1 CH_2_Cl_2_/H_2_O mixed solvent produced expected sulfonium salt **3a** in 80% yield after 3 h (Table 1, entry 2). The change in the reagent **1a**:substrate **2a** ratio to 1.2:1 further improved the yield to quantitative (Table 1, entry 3). Reducing the reaction time to 1 h decreased the yield to 79% (Table 1, entry 4). Using pure water, without any organic solvent, resulted in a 75% yield of sulfonium salt **3a** under the optimized conditions (Table 1, entry 5), probably due to the low solubility of the reactants in water. Using aqueous acetonitrile resulted in a slightly lower yield (Table 1, entry 6), and several other organic solvents were less efficienct (Table 1, entries 7–13). The reactions of reagent **1a** were highly regioselective, producing only *m*-fluoro-substituted sulfonium salt **3a**, which is in agreement with previously reported electronic effects on the regioselectivity of reactions of substituted benzyne intermediates.^1b^

**Table 1:**
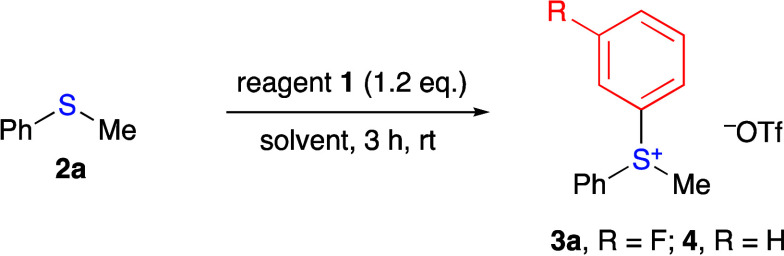
Optimization of the Reaction Conditions

entry	reagent	solvent or additive	product (%)^a^
1	**1a**	CH_2_Cl_2_	–b
2^c^	**1a**	CH_2_Cl_2_/H_2_O (9:1)	**3a** (80)
3	**1a**	CH_2_Cl_2_/H_2_O (9:1)	**3a** (quant)
4^d^	**1a**	CH_2_Cl_2_/H_2_O (9:1)	**3a** (79)
5	**1a**	H_2_O	**3a** (75)
6	**1a**	MeCN/H_2_O (9:1)	**3a** (94)
7	**1a**	Cl(CH_2_)_2_Cl/H_2_O (9:1)	**3a** (60)
8	**1a**	CHCl_3_/H_2_O (9:1)	**3a** (81)
9	**1a**	AcOEt/H_2_O (9:1)	**3a** (86)
10	**1a**	PhH/H_2_O (9:1)	**3a** (64)
11	**1a**	heptane/H_2_O (9:1)	**3a** (63)
12	**1a**	ether/H_2_O (9:1)	**3a** (74)
13	**1a**	MeOH/H_2_O (9:1)	**3a** (29)
14	**1b**	CH_2_Cl_2_/H_2_O (9:1)	**4** (trace)
15	**1b**	CH_2_Cl_2_/saturated NaHCO_3_ (9:1)	**4** (85)

a Isolated yields of products.

b Unreacted reagent **1a** was recovered from the reaction mixture; product **3** was not formed.

c **1a**:**2** ratio of 1:1.2.

d Reaction time of 1 h.

Compared to reagent **1a**, reagent **1b** is less reactive in this reaction. Only a trace amount of the corresponding product **4** was detected when a solution of reagent **1b** and sulfide **2a** was stirred in a 9:1 CH_2_Cl_2_/H_2_O mixed solvent for 3 h at room temperature (Table 1, entry 14). However, the addition of saturated aqueous NaHCO_3_ to the reaction mixture improved the yield to 85% (Table 1, entry 15). Considering the higher reactivity of reagent **1a**, we used this reagent under the optimized conditions in our next studies.

On the basis of the results presented above (Table 1, entry 3), the preparation of various sulfonium salts **3** from sulfides **2** and reagent **1a** was carried out under the optimized reaction conditions (Scheme 3). The reaction of various alkyl aryl sulfides **2** afforded the corresponding diaryl alkyl sulfonium salts **3a**–**m** in excellent yields, demonstrating compatibility with various substituents (alkyl, halogen, CN, NO_2_, CHO, and cyclopropyl) in the aromatic ring or alkyl group of sulfide **2**. Interestingly, the cyano and aldehyde functional groups do not react with aryne under these conditions. It is noteworthy that the reaction of phenyl ethyl sulfide with aryne generated from reagent **1a** and water produced sulfonium salt **3j** in quantitative yield. For comparison, the previously reported similar reaction of aryl ethyl sulfides with benzyne generated from benzenediazonium-2-carboxylate under anhydrous conditions proceeded with elimination of ethylene leading to the corresponding diaryl sulfides (ArSPh) in 90–96% yields.^4^ 2-Chloroethyl phenyl sulfide reacts with reagent **1a** to afford the product of intermolecular cyclization **3v**, which is in agreement with initial zwitterionic intermediate **B** (Scheme 1) in this reaction. The involvement of intermediate **B** is also supported by the control experiment using a 9:1 CH_2_Cl_2_/D_2_O mixed solvent, which yielded deuterated product **5** (Scheme 2). For comparison, the reaction of **1a** in a 9:1 CD_2_Cl_2_/H_2_O mixed solvent under the same conditions afforded exclusively nondeuterated product **3n**. It should be noted that in the previously reported D-labeling experiments for the reactions of benzyne with diaryl sulfides under anhydrous conditions,^5^ the organic solvent (acetonitrile) was found to be the main source of the hydrogen atom in this reaction.

**Scheme 2:**
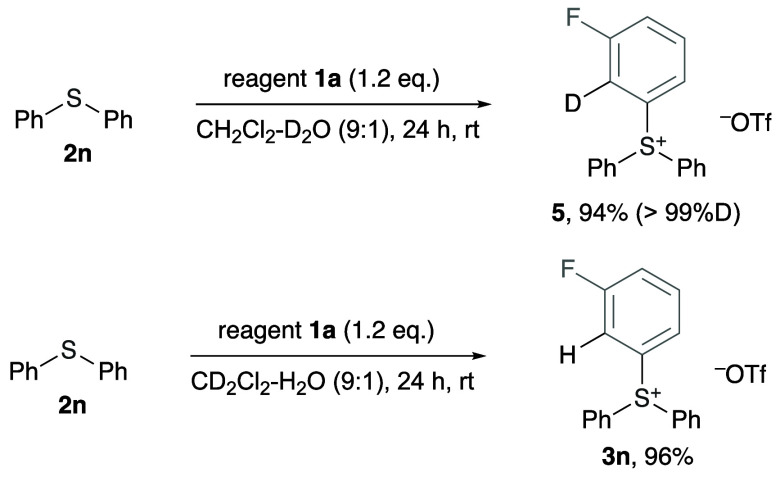
Reactions in the Presence of D_2_O or CD_2_Cl_2_

**Scheme 3:**
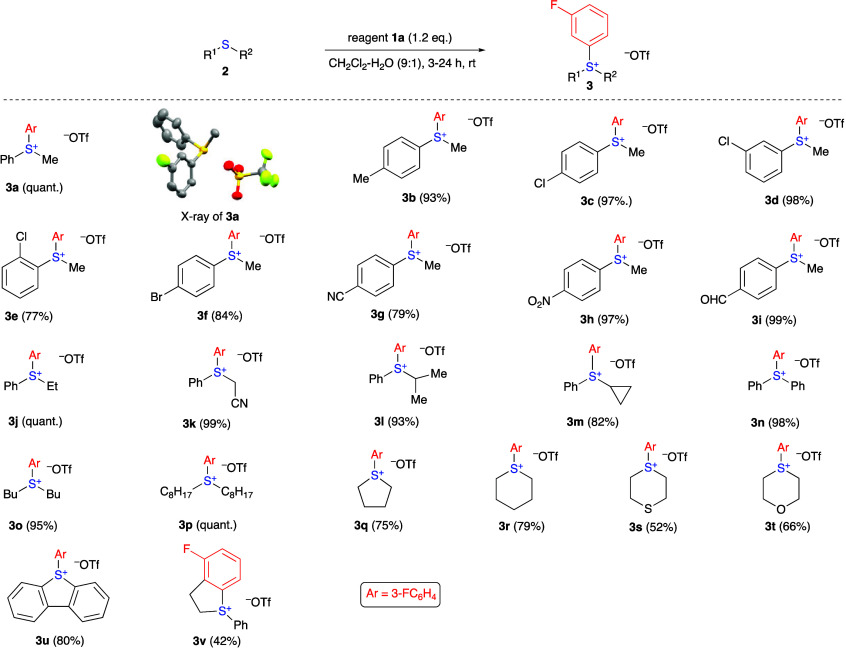
Substrate Scope and Products (**3a–v**) of the Reactions of Organic Sulfides **2** with Reagent **1a**, All reactions were conducted with 0.1 mmol of sulfide **2** and 0.12–0.2 mmol of reagent **1a** in dichloromethane (0.9 mL) and water (0.1 mL) under stirring. Isolated yields of products **3**.

To gain additional insight into the reaction mechanism, we performed a Hammett plot study of the reaction of reagent **1a** with five *para*-substituted thioanisoles (see the Supporting Information for details). The results showed that the introduction of an electron-donating group such as methyl accelerated the reaction, while the introduction of electron-withdrawing groups such as Cl, CHO, and NO_2_ decelerated it. The Hammett plot showed the good correlation of the relative rate factors with σ_p_ constants and gave a reaction constant ρ of −0.65 (*r* = 0.98). The small negative ρ values could be consistent with the results suggesting that generated aryne species **8** are highly active electrophilic species.^6^

It is important to note that the reactions of diphenyl sulfide and dibenzothiophene with reagent **1a** at room temperature afforded triaryl sulfonium salts **3n** and **3u**, respectively, in excellent yields. For comparison, the most common approach to triaryl sulfonium salts is based on the transfer of an aryl from diaryliodonium(III) salts to diaryl sulfides at 120–230 °C.^7^ Recently, a milder preparation of triaryl sulfonium salts using the Kobayashi aryne precursor was reported.^5^ However, this method works only under anhydrous conditions, and acetonitrile serves as the source of protons, as proven by the labeling experiments with CD_3_CN.^5^ The reactions of dialkyl sulfides with reagent **1a** afford the respective dialkyl aryl sulfonium salts **3o**–**t**, including cyclic sulfonium salts **3q**–**t**. Interestingly, the reaction with 1,4-dithiane **2s** selectively afforded product of monoarylation **3s**, even with an excess of **1a**.

The reactions of reagent **1a** can be further extended to organic selenides and sulfoxides. In particular, reagent **1a** reacts with Ph_2_Se in a 9:1 CH_2_Cl_2_/H_2_O mixed solvent at room temperature to give the corresponding triaryl selenonium salt in 98% yield (see product **3w** in the Supporting Information). Dimethyl sulfoxide **6a** or methyl phenyl sulfoxide **6b** reacts with **1a** under the standard conditions, forming *o*-hydroxy-substituted sulfonium salts **7a** and **7b** (Scheme 4). The structure of product **7a** was confirmed by X-ray analysis. We suggest that the mechanism of this reaction involves initial nucleophilic addition of sulfoxide to aryne **8** forming zwitterionic intermediate **9** and four-membered cyclic intermediate **10** that finally reacts with water to give product **7** (Scheme 4). Note that mesityl iodide, formed as a byproduct by reductive elimination, was detected by nuclear magnetic resonance (NMR) in the reaction mixture. This mechanism is in agreement with previously reported reactions of benzyne generated from the Kobayashi precursor with diaryl sulfoxides.^1e,8^ The previously reported reaction of sulfoxide under aprotic conditions forms *o*-aryloxy arylsulfonium salts as final products via intermediates **9** and **10**.^8^

**Scheme 4:**
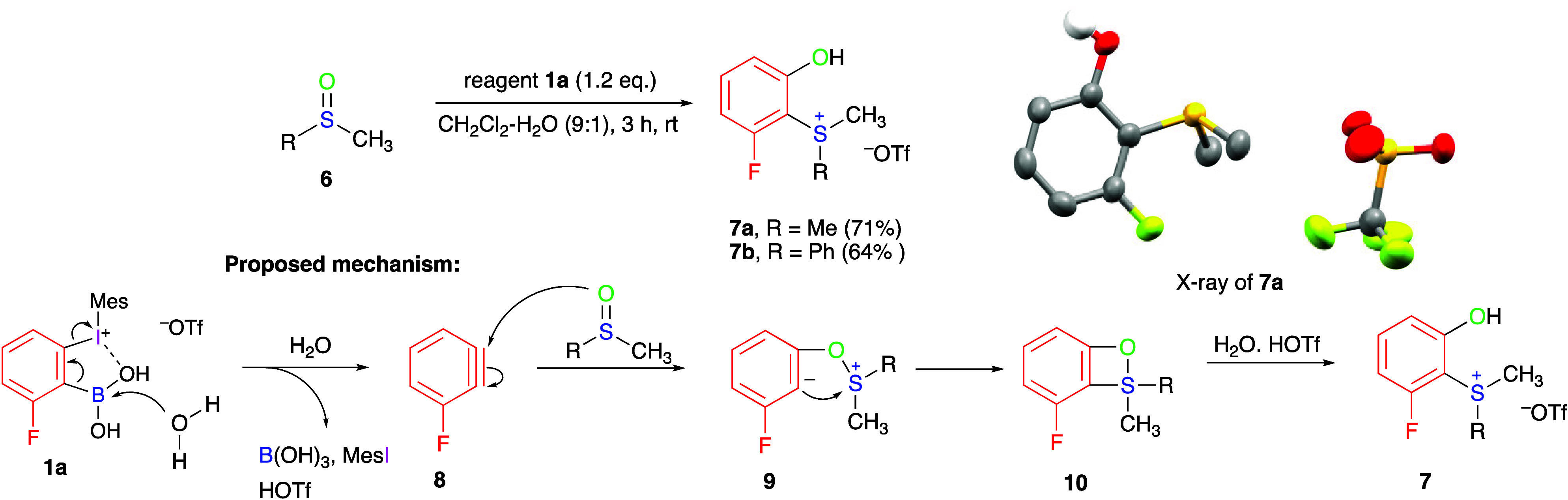
Reactions of Aryne Intermediates with Organic Sulfoxides

Sulfonium salts **3** and **7** prepared by our procedure are potentially important products with many possible applications.^3^ As one of the additional applications, we have found that sulfonium salt **3a** is an efficient methylating reagent in reactions with arylsulfinate anions and the carboxylate anion (see section 8 of the Supporting Information for the preparation of products **11**–**13**).

In summary, we have demonstrated that pseudocyclic arylbenziodoxaborole **1a** is a unique benzyne precursor under neutral aqueous conditions that reacts with organic sulfides, producing the corresponding sulfonium salts. This reaction is compatible with various substituents (alkyl, halogen, CN, NO_2_, CHO, and cyclopropyl) in the aromatic ring or alkyl group of sulfide. Sulfoxides react with reagent **1a** under these conditions, forming *o*-hydroxy-substituted sulfonium salts **7**. The structures of key products were confirmed by X-ray crystallography, and a mechanistic rationalization has been provided.

## Data Availability

The data underlying this study are available in the published article and its Supporting Information.

## Supporting Information Available

Experimental procedures, compound characterization data, and NMR spectra for all new compounds (PDF)
