# Supplementary material for: Pseudocyclic Arylbenziodoxaboroles as Water-Triggered Aryne Precursors in Reactions with Organic Sulfides
Source: Org Lett. 2024 Feb 26;26(9):1891–5. doi: 10.1021/acs.orglett.4c00197 (PMC10928713; doi:10.1021/acs.orglett.4c00197)

Supporting Information

Pseudocyclic Arylbenziodoxaboroles as Water-Triggered Aryne Precursors in Reactions with Organic Sulfides

Akira Yoshimura,[a]* Kim Ngo,[b] Irina A. Mironova,[c] Zachary S. Gardner,[b] Gregory T. Rohde,[d] Nami Ogura,[a] Akiharu Ueki,[a] Mekhman S. Yusubov,[c] Akio Saito,[e] and Viktor V. Zhdankin [b]*

[a] Faculty of Pharmaceutical Sciences, Aomori University, 2-3-1 Kobata, Aomori 030-0943, Japan
[b] Department of Chemistry and Biochemistry, University of Minnesota Duluth, Duluth, Minnesota 55812, USA
[c] Research School of Chemistry and Applied Biomedical Sciences, Tomsk Polytechnic University, Lenina av., 30, 634050 Tomsk, Russia
[d] Marshall School, Duluth, Minnesota 55811, USA
[e] Division of Applied Chemistry, Institute of Engineering, Tokyo University of Agriculture and Technology, 2-24-16 Naka-cho, Koganei, Tokyo 184-8588, Japan

- Emails: ayoshimura@aomori-u.ac.jp (Akira Yoshimura), vzhdanki@d.umn.edu (Viktor V. Zhdankin)

Table of Contents pp

1. General experimental remarks 2
2. Gram-scale preparation of mesityl-2-fluoro-1-phenylboronic acid-6-iodonium triflate **1a** 2
3. Reactions of mesityl-2-fluoro-1-phenylboronic acid-6-iodonium triflate **1a** with sulfides or selenide 3
4. Reactions of mesityl-1-phenyboronic acid-2-iodonium triflate **1b** with thioanisole 19
5. Control experiments 20
6. Reactions of mesityl-2-fluoro-1-phenylboronic acid-6-iodonium triflate **1a** with dimethylsulfoxide or methyl phenyl sulfoxide 21
7. Hammett plot experiments 23
8. Reactions of 3-fluorophenyl-methyl-phenylsulfonium triflate with nucleophiles 24
9. References 26
10. Spectra of products 27

1. General experimental remarks

All reactions were performed under dry argon atmosphere with flame-dried glassware. All commercial reagents were ACS reagent grade and used without further purification. Dichloromethane and acetonitrile were distilled from CaH_2 immediately prior to use. Diethyl ether was distilled from Na/benzophenone. Melting points were determined in an open capillary tube with a Mel-temp II melting point apparatus. Infrared spectra were recorded as a KBr pellet on a Perkin-Elmer 1600 series FT-IR spectrophotometer. ^{1}H NMR, ^{13}C NMR, and ^{19}F NMR spectra were recorded on a Bruker 400 MHz NMR spectrometer. Chemical shifts are reported in parts per million (ppm). ^{1}H and ^{13}C chemical shifts are referenced relative to tetramethylsilane. High resolution mass spectra (HRMS) were obtained on a Bruker MicrOTOF-III high resolution mass-spectrometer using electrospray ionization (ESI). X-ray crystal analysis of compounds **3a** and **7a** was performed by Rigaku RAPID II XRD Image Plate using graphite-monochromated Cu or Mo Kα radiation (λ = 1.54187 or 0.71073 Å) at 125 or 173 K. See the cif files for more detailed crystallography information. Mesityl-2-fluoro-1-phenylboronic acid-6-iodonium triflate **1a** and mesityl-1-phenylboronic acid-2-iodonium triflate **1b** were prepared according to the reported procedures.[1] Compounds 2**a-w** and **6** were purchased from commercial sources.

2. Gram-scale preparation of mesityl-2-fluoro-1-phenylboronic acid-6-iodonium triflate 1a[1]

A solution of 2-fluoro-6-iodophenylboronic acid (1.250 g, 4.7 mmol) in acetic acid (6 mL) was cooled at 0 °C to add NaOCl (6.5 mL) under stirring. The stirring was continued at rt for 4.5 hour. After reaction, the white precipitate was filtered, washed with water, hexane,

and ether, then dried in vacuum to afford the white solid of 1-acetoxy-benziodoxaborole.[1a] The white solid was dissolved in dichloromethane (7.0 mL), cooled to 0 °C and TfOH (1.411g, 9.4 mmol) was added under stirring. The reaction mixture was stirred at 0 °C for 2 hours, then mesitylene (12 mL) was added to the reaction mixture, and stirring was continued for overnight at room temperature. After reaction, the solvent was removed under reduced pressure and received oil compound, which was washed with hexane and diethyl ether several times then dried in vacuum to receive the mesityl-2-fluoro-1-phenylboronic acid-6-iodonium triflate **1a** 1.959 g (78% yield; 2 steps), mp 154-155 °C.[1b] ^{1}H NMR (400 MHz, CD_3CN): δ 7.50-7.43 (m, 1H), 7.42-7.37 (m, 1H), 7.35 (m, 2H), 6.76 (d, J = 8.4 Hz, 1H), 2.54 (s, 6H), 2.45 (s, 3H).

3. Reaction of mesityl-2-fluoro-1-phenylboronic acid-6-iodonium triflate with sulfides or selenide.

Sulfide **2a-v** (0.1 mmol, 1 equiv.) or diphenyl selenide (0.1 mmol, 1 equiv.) was added to a solution of mesityl-2-fluoro-1-phenylboronic acid-iodonium triflate **1a** (0.12 to 0.20 mmol, 1.2 to 2.0 equiv.) in dichloromethane (0.9 mL) and water (0.1 mL). The reaction was stirred at room temperature for 3-24 hours. After reaction, water (5 mL) was added, and the mixture was extracted with dichloromethane. The organic layer was dried over by anhydrous Na_2SO_4 and concentrated under reduced pressure. The crude mixture was washed with diethyl ether several times and then dried in vacuum to give the analytically pure sulfonium triflate **3** or triphenyl selenium triflate **3w**.

3-Fluorophenyl-methyl-phenylsulfonium triflate (3a)

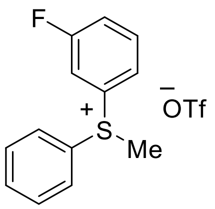

Reaction of thioanisole **2a** (12.4 mg, 0.1 mmol) and **1a** (64.1 mg, 0.12 mmol) according to the general procedure afforded 36.7 mg (100%) of product **3a**, isolated as a white solid: mp 118.8-119.3 °C; IR (KBr) cm^{-1} 3100, 3072, 3025, 2940, 1597, 1480, 1255, 1222, 1168, 1028, 636; ^{1}H NMR (400 MHz, $CDCl_3$): δ 7.92 (d, *J* = 7.2, 2H), 7.85-7.80 (m, 1H), 7.80-7.74 (m, 1H), 7.74-7.66 (m, 3H), 7.52 (dt, *J* = 7.6, 2.2 Hz, 1H), 7.47-7.38 (m, 1H), 3.78 (s, 3H); ^{13}C NMR (100 MHz, CD_3CN): δ 163.5 (d, $^1J_{CF}$ = 250.4 Hz), 135.2, 133.7 (d, $^3J_{CF}$ = 8.5 Hz), 131.8, 130.7, 128.6 (d, $^3J_{CF}$ = 8.0 Hz), 126.8 (d, $^4J_{CF}$ = 3.0 Hz), 126.2, 122.1 (d, $^2J_{CF}$ = 21.1 Hz), 121.7 (q, $^1J_{CF}$ = 318.8 Hz), 117.6 (d, $^2J_{CF}$ = 26.0 Hz), 28.2. ^{19}F NMR (376 MHz, CD_3CN): δ -108.7, -79.3; HRMS (ESI) m/z: [M-OTf]$^+$ Calcd for $C_{13}H_{12}FS$ 219.0638; Found 219.0633.

Crystal structure determination of compound **3a**:

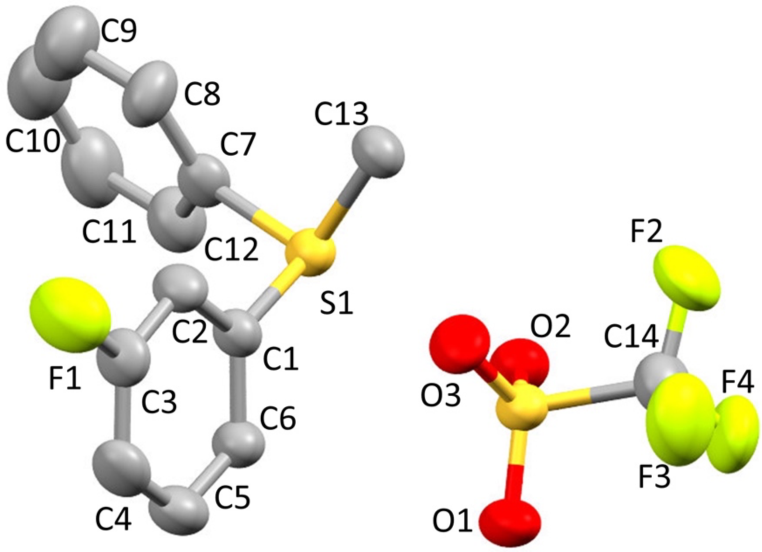

Figure S1: Thermal ellipsoid plot of **3a** drawn 50% probability level. Hydrogen atoms were removed for clarity.

Single crystals of product **3a** suitable for X-ray crystallographic analysis were obtained by slow crystallization from acetonitrile solution. X-ray diffraction data for **3a** were collected on Rigaku RAPID II Image Plate system using graphite-monochromated Cu Kα radiation (λ = 1.54187 Å) at 173 K. The structure was solved by Superflip[2] and refined using SHELXL-2014/7.[3] Crystal data for **3a** $C_{13}H_{12}F_4O_3S_2$, *M* = 368.36 monoclinic, space group P2(1)/c, a = 12.2918(2) Å, b = 9.1218(2) Å, c = 15.3679(11) Å, α = 90 °, β = 111.577(8) °, γ = 90 °, V = 1602.35(15) $Å^3$, Z = 4, 21498 reflections measured (R_{int} = 0.0457), 2832 unique reflections, 1813 I > 2/s(I), 209 parameters, 0 restraints; GooF = 1.229, final R1 = 0.0655, Rw (all) = 0.1005. CCDC 2312444.

Larger scale preparation of compound **3a:**

Thioanisole **2a** (124.2 mg, 1 mmol) was added to a solution of mesityl-2-fluoro-1-phenylboronic acid-iodonium triflate **1a** (640.9 mg, 1.2 mmol) in dichloromethane (9 mL) and water (1 mL). The reaction was stirred at room temperature for 3 hours. After reaction, water (50 mL) was added, and the mixture was extracted with dichloromethane. The organic layer was dried over by anhydrous Na_2SO_4 and concentrated under reduced pressure. The crude mixture was washed with diethyl ether several times and then dried in vacuum to give 327 mg (89%) of product **3a**.

3-Fluorophenyl-methyl-4-methylphenylsulfonium triflate (3b)

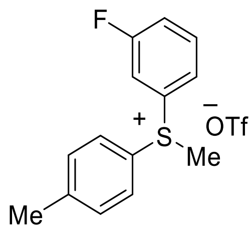

Reaction of methyl *p*-tolyl sulfide **2b** (13.8 mg, 0.1 mmol) and **1a** (64.1 mg, 0.12 mmol) according to the general procedure afforded 35.5 mg (93%) of product **3b**, isolated as a white solid: mp 120.1-120.6 °C; IR (KBr) cm^{-1} 3072, 3037, 2941, 2879, 1597, 1480, 1258, 1222, 1163, 1031, 636; ^{1}H NMR (400 MHz, $CDCl_3$): δ 7.80 (d, J = 7.8 Hz, 2H), 7.74-7.67 (m, 1H), 7.56-7.45 (m, 3H), 7.41 (t, J = 8.0 Hz, 1H), 3.75 (s, 3H), 2.48 (s, 3H); ^{13}C NMR (100 MHz, CD_3CN): δ 163.5 (d, $^{1}J_{CF}$ = 249.0 Hz), 147.0, 133.7 (d, $^{3}J_{CF}$ = 8.2 Hz), 132.4, 130.7, 129.1 (d, $^{3}J_{CF}$ = 9.4 Hz), 126.5 (d, $^{4}J_{CF}$ = 3.4 Hz), 122.4, 121.9 (d, $^{2}J_{CF}$ = 21.3 Hz), 121.7 (q, $^{1}J_{CF}$ = 320.3 Hz), 117.4 (d, $^{2}J_{CF}$ = 26.0 Hz), 28.3, 21.2. ^{19}F NMR (376 MHz, $CDCl_3$): δ -104.9, -78.3; HRMS (ESI) m/z: $[M-OTf]^+$ Calcd for $C_{14}H_{14}FS$ 233.0795; Found 233.0793.

4-Chlorophenyl-3-fluorophenyl-methylsulfonium triflate (3c)

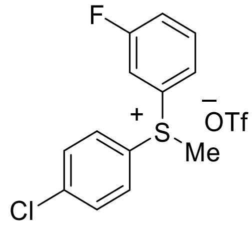

Reaction of 4-chlorothioanisole **2c** (12.9 mg, 0.081 mmol) and **1a** (52.0 mg, 0.097 mmol) according to the general procedure afforded 31.6 mg (97%) of product **3c**, isolated as a white solid: mp 120.1-120.6 °C; IR (KBr) cm^{-1} 3073, 3027, 2941, 2896, 1596, 1483, 1256, 1219, 1164, 1099, 1031, 636; ^{1}H NMR (400 MHz, $CDCl_3$): δ 7.90 (d, J = 8.8 Hz, 2H), 7.82 (d, J = 8.4 Hz, 1H), 7.75 (dd, J = 8.4 Hz, 8.0 Hz, 1H), 7.69 (d, J = 8.8 Hz, 2H), 7.54-7.51 (m, 1H), 7.46 (dt, J = 7.8 Hz, 2.0 Hz, 1H), 3.80 (s, 3H); ^{13}C NMR (100 MHz, CD_3CN): δ

163.5 (d, $^{1}J_{CF}$ = 250.9 Hz), 141.3, 133.7 (d, $^{3}J_{CF}$ = 8.3 Hz), 132.5, 131.9, 128.2 (d, $^{3}J_{CF}$ = 8.1 Hz), 126.9 (d, $^{4}J_{CF}$ = 3.0 Hz), 124.9, 122.3 (d, $^{2}J_{CF}$ = 20 Hz), 121.7 (q, $^{1}J_{CF}$ = 319.2 Hz), 117.6, 28.4. ^{19}F NMR (376 MHz, CD_3CN): δ -108.6, -79.3; HRMS (ESI) m/z: $[M\text{-}OTf]^+$ Calcd for $C_{13}H_{11}Cl^{35}FS$ 253.0249; Found 253.0244.

3-Chlorophenyl-3-fluorophenyl-methylsulfonium triflate (3d)

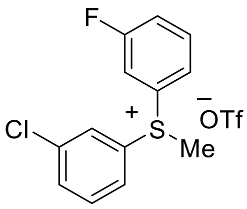

Reaction of 3-chlorothioanisole **2d** (15.9 mg, 0.1 mmol) and **1a** (64.1 mg, 0.12 mmol) according to the general procedure afforded 39.5 mg (98%) of product **3d**, isolated as a white solid: mp 92.9-93.5 °C; IR (KBr) cm^{-1} 3072, 3021, 2937, 1597, 1479, 1251, 1222, 1164, 1085, 1028, 636; ^{1}H NMR (400 MHz, CD_3CN): δ 7.96 (s, 1H), 7.87-7.79 (m, 2H), 7.79-7.67 (m, 4H), 7.58 (t, J = 8.8 Hz, 1H), 3.64 (s, 3H); ^{13}C NMR (100 MHz, CD_3CN): δ 163.5 (d, $^{1}J_{CF}$ = 250.5 Hz), 136.9, 135.2, 133.8 (d, $^{3}J_{CF}$ = 8.3 Hz), 133.2, 130.3, 129.3, 127.9 (d, $^{3}J_{CF}$ = 8.1 Hz), 127.1 (d, $^{4}J_{CF}$ = 3.5 Hz), 122.4 (d, $^{2}J_{CF}$ = 21 Hz), 121.7 (q, $^{1}J_{CF}$ = 318.7 Hz), 116.9, 28.2. ^{19}F NMR (376 MHz, CD_3CN): δ -108.5, -79.3; HRMS (ESI) m/z: $[M\text{-}OTf]^+$ Calcd for $C_{13}H_{11}Cl^{35}FS$ 253.0249; Found 253.0247.

2-Chlorophenyl-3-fluorophenyl-methylsulfonium triflate (3e)

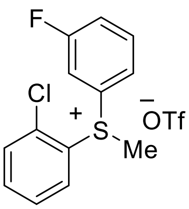

Reaction of 2-chlorothioanisole **2e** (15.9 mg, 0.1 mmol) and **1a** (64.1 mg, 0.12 mmol)

according to the general procedure afforded 30.9 mg (77%) of product **3e**, isolated as a white solid: mp 152.9-153.4 °C; IR (KBr) cm^{-1} 3078, 3050, 3939, 1597, 1482, 1225, 1166, 1145, 1029, 637; ^1H NMR (400 MHz, $CDCl_3$): δ 8.28 (dd, J = 7.8 Hz, 1.4 Hz, 1H), 7.88 (d, J = 8.0 Hz, 1H),7.83-7.70 (m, 3H), 7.65 (d, J = 7.6 Hz, 1H), 7.57 (dt, J = 7.2 Hz, 2.4 Hz, 1H), 7.46 (t, J = 8.2 Hz, 1H), 3.86 (s, 3H); ^{13}C NMR (100 MHz, CD_3CN): δ 163.6 (d, $^1J_{CF}$ = 250.8 Hz), 136.5, 135.9, 133.8 (d, $^3J_{CF}$ = 8.3 Hz), 132.4, 130.8, 130.5, 127.5 (d, $^4J_{CF}$ = 3.6 Hz), 127.0 (d, $^3J_{CF}$ = 8.3 Hz), 124.4, 122.6 (d, $^2J_{CF}$ = 20.9 Hz), 121.7 (q, $^1J_{CF}$ = 318.6 Hz), 118.3 (d, $^2J_{CF}$ = 25.9 Hz), 27.6. ^{19}F NMR (376 MHz, CD_3CN): δ -108.4, -79.3; HRMS (ESI) m/z: $[M-OTf]^+$ Calcd for $C_{13}H_{11}Cl^{35}FS$ 253.0249; Found: 253.0248.

4-Bromophenyl-3-fluorophenyl-methylsulfonium triflate (3f)

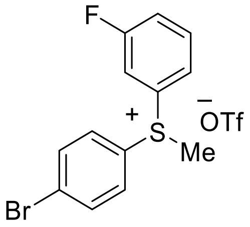

Reaction of 4-bromothioanisole **2f** (20.2 mg, 0.1 mmol) and **1a** (64.1 mg, 0.12 mmol) according to the general procedure afforded 37.4 mg (84%) of product **3f**, isolated as a white solid: mp 112.2-113.3 °C; IR (KBr) cm^{-1} 3072, 3026, 2918, 1596, 1479, 1256, 1220, 1165, 1031, 637; ^1H NMR (400 MHz, $CDCl_3$): δ 7.87-7.77 (m, 5H), 7.74-7.67 (m, 1H), 7.56 (d, J = 7.6 Hz, 1H), 7.44 (t, J = 8.2 Hz, 1H), 3.79 (s, 3H); ^{13}C NMR (100 MHz, CD_3CN): δ 163.5 (d, $^1J_{CF}$ = 250.5 Hz), 134.9, 133.7 (d, $^3J_{CF}$ = 8.3 Hz), 132.4, 129.8, 128.1 (d, $^3J_{CF}$ = 8.2 Hz), 126.9 (d, $^4J_{CF}$ = 3.3 Hz), 125.5, 122.3 (d, $^2J_{CF}$ = 21.1 Hz), 121.7 (q, $^1J_{CF}$ = 318.9 Hz), 117.7 (d, $^2J_{CF}$ = 19.3 Hz), 28.3. ^{19}F NMR (376 MHz, CD_3CN): δ -108.6, -79.3.; HRMS (ESI) m/z: $[M-OTf]^+$ Calcd for $C_{13}H_{11}Br^{79}FS$ 296.9743; Found 296.9747.

4-Cyanophenyl-3-fluorophenyl-methylsulfonium triflate (3g)

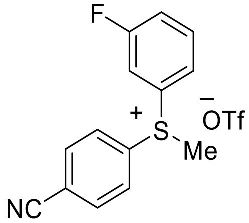

Reaction of 4-(methylthio)benzonitrile **2g** (14.9 mg, 0.1 mmol) and **1a** (64.1 mg, 0.12 mmol) according to the general procedure for 24 hours afforded 31.1 mg (79%) of product **3g**, isolated as a white solid: mp 134.6-135.3 °C; IR (KBr) cm^{-1} 3067, 3036, 3020, 2935, 2238, 1595, 1480, 1253, 1224, 1153, 1028, 636; ^{1}H NMR (400 MHz, $CDCl_3$): δ 8.03 (d, J = 8.0 Hz, 2H), 7.94 (d, J = 8.8 Hz, 2H), 7.80-7.64 (m, 3H), 7.62-7.55 (m, 1H), 3.60 (s, 3H); ^{13}C NMR (100 MHz, CD_3CN): δ 163.6 (d, $^{1}J_{CF}$ = 250.7 Hz), 135.2, 133.9 (d, $^{3}J_{CF}$ = 8.4 Hz), 131.8, 131.3, 127.6 (d, $^{4}J_{CF}$ = 3.4 Hz), 127.2 (d, $^{3}J_{CF}$ = 8.3 Hz), 122.7 (d, $^{2}J_{CF}$ = 21 Hz), 121.7 (q, $^{1}J_{CF}$ = 318.8 Hz), 118.5, 118.3 (d, $^{2}J_{CF}$ = 26.0 Hz), 28.2; HRMS (ESI) m/z: $[M-OTf]^+$ Calcd for $C_{14}H_{11}FNS$ 244.0591; Found: 244.0604.

3-Fluorophenyl-methyl-4-nitrophenylsulfonium triflate (3h)

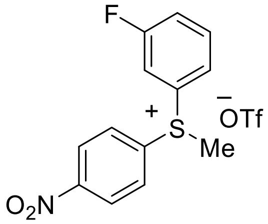

Reaction of 4-nitrothioanisole **2h** (16.9 mg, 0.1 mmol) and **1a** (64.1 mg, 0.12 mmol) according to the general procedure for 6 hours afforded 40.1 mg (97%) of product **3h**, isolated as a white solid: mp 136.5-137.2 °C; IR (KBr) cm^{-1} 3108, 3067, 3031, 2918, 1596, 1524, 1483, 1349, 1281, 1224, 1151, 1031, 857, 641; ^{1}H NMR (400 MHz, CD_3CN): δ 8.48 (d, J = 8.8 Hz, 2H), 8.06 (d, J = 8.8 Hz, 2H), 7.84-7.69 (m, 1H), 7.62 (t, J = 8.4 Hz, 1H),

3.67 (s, 3H); ^{13}C NMR (100 MHz, CD_3CN): δ 163.4 (d, $^1J_{CF}$ = 250.9 Hz), 151.5, 133.7 (d, $^3J_{CF}$ = 8.4 Hz), 133.0, 131.9, 127.5 (d, $^4J_{CF}$ = 3.5 Hz), 126.3 (d, $^3J_{CF}$ = 8.3 Hz), 126.1, 122.1 (d, $^2J_{CF}$ = 21.2 Hz), 121.4 (q, $^1J_{CF}$ = 318.4 Hz), 118.1 (d, $^2J_{CF}$ = 26.1 Hz), 28.2; HRMS (ESI) m/z: [M-OTf]$^+$ Calcd for $C_{13}H_{11}FNO_2S$ 264.0489; Found 264.0492.

4-(3-Fluorophenyl-methylsulfonium)benzaldehyde triflate (3i)

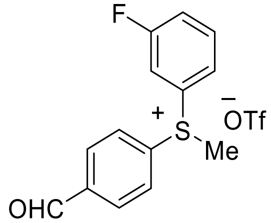

Reaction of 4-(methylthio)benzaldehyde **2i** (15.2 mg, 0.1 mmol) and **1a** (64.1 mg, 0.12 mmol) according to the general procedure afforded 39.4 mg (99%) of product **3i**, isolated as a white solid: mp 104.9-106.2 °C; IR (KBr) cm^{-1} 3067, 3026, 2931, 1699, 1595, 1481, 1255, 1225, 1157, 1028, 636; ^{1}H NMR (400 MHz, $CDCl_3$): δ 10.1 (s, 1H), 8.17 (d, J = 8.4 Hz, 2H), 8.12 (d, J = 8.4 Hz, 2H), 7.83 (d, J = 8.0 Hz, 1H), 7.78-7.70 (m, 1H), 7.58 (d, J = 7.2 Hz, 1H), 7.52-7.45 (m, 1H), 3.87 (s, 3H); ^{13}C NMR (100 MHz, CD_3CN): δ 192.0, 163.6 (d, $^1J_{CF}$ = 250.6 Hz), 140.6, 133.9 (d, $^3J_{CF}$ = 8.4 Hz), 132.0, 131.9, 131.3, 127.5 (d, $^3J_{CF}$ = 8.3 Hz), 127.4 (d, $^4J_{CF}$ = 3.3 Hz), 122.6 (d, $^2J_{CF}$ = 21.2 Hz), 121.7 (q, $^1J_{CF}$ = 318.2 Hz), 118.3, 28.2; HRMS (ESI) m/z: [M-OTf]$^+$ Calcd for $C_{14}H_{12}FOS$ 247.0587; Found 247.0604.

3-Fluorophenyl-ethyl-phenylsulfonium triflate (3j)

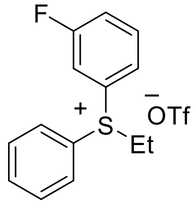

Reaction of ethyl phenyl sulfide **2j** (13.8 mg, 0.1 mmol) and **1a** (64.1 mg, 0.12 mmol) according to the general procedure afforded 38.1 mg (100%) of product **3j**, isolated as a light yellow oil; IR (neat) cm^{-1} 3068, 3010, 2963, 1594, 1480, 1259, 1225, 1159, 1030, 637; ^{1}H NMR (400 MHz, $CDCl_3$): δ 8.06-7.88 (m, 3H), 7.85-7.66 (m, 1H), 7.58 (d, J = 7.6 Hz, 1H), 7.44 (t, J = 8.2 Hz, 1H), 4.42-4.28 (m, 2H), 1.52 (t, J = 7.2 Hz, 3H); ^{13}C NMR (100 MHz, CD_3CN): δ 163.6 (d, $^{1}J_{CF}$ = 250.7 Hz), 135.5, 133.9 (d, $^{3}J_{CF}$ = 8.3 Hz), 132.0, 131.4, 127.5 (d, $^{4}J_{CF}$ = 3.5 Hz), 126.7 (d, $^{3}J_{CF}$ = 8.2 Hz), 124.2, 122.4 (d, $^{2}J_{CF}$ = 21 Hz), 121.8 (q, $^{1}J_{CF}$ = 318.7 Hz), 118.2 (d, $^{2}J_{CF}$ = 25.9 Hz), 40.8, 9.3. ^{19}F NMR (376 MHz, CD_3CN): δ -108.5, -79.3; HRMS (ESI) m/z: $[M-OTf]^+$ Calcd for $C_{14}H_{14}FS$ 233.0795; Found 233.0809.

Cyanomethyl-3-fluorophenyl-phenylsulfonium triflate (3k)

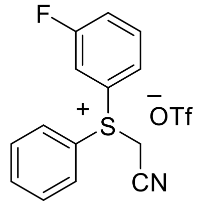

Reaction of 2-(phenylthio)acetonitrile **2k** (14.9 mg, 0.1 mmol) and **1a** (64.1 mg, 0.12 mmol) according to the general procedure afforded 39.0 mg (99%) of product **3k**, isolated as a white amorphous solid; IR (KBr) cm^{-1} 3074, 2959, 2922, 2275, 1595, 1480, 1257, 1228, 1164, 1030, 638; ^{1}H NMR (400 MHz, CD_3CN): δ 7.99-7.93 (m, 2H), 7.89-7.81 (m, 3H), 7.78-7.62 (m, 3H), 5.14 (s, 2H).; ^{13}C NMR (100 MHz, CD_3CN): δ 163.6 (d, $^{1}J_{CF}$ = 251.6 Hz), 136.5, 134.2 (d, $^{3}J_{CF}$ = 8.5 Hz), 132.3, 131.9, 127.9 (d, $^{4}J_{CF}$ = 3.6 Hz), 124.4 (d, $^{3}J_{CF}$ = 8.4 Hz), 123.5 (d, $^{2}J_{CF}$ = 21.2 Hz), 122.1, 121.6 (q, $^{1}J_{CF}$ = 318.2 Hz), 118.7 (d, $^{2}J_{CF}$ = 26.5 Hz), 110.6, 34.0.; ^{19}F NMR (376 MHz, CD_3CN): δ -107.8, -79.4; HRMS (ESI) m/z: $[M-OTf]^+$ Calcd for $C_{14}H_{11}FNS$ 244.0591; Found 244.0612.

3-Fluorophenyl-isopropyl-phenylsulfonium triflate (3l)

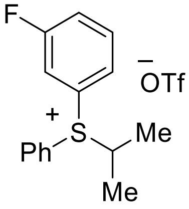

Reaction of isopropyl phenyl sulfide **2l** (15.2 mg, 0.1 mmol) and **1a** (64.1 mg, 0.12 mmol) according to the general procedure afforded 37 mg (93%) of product **3l**, isolated as a colorless oil; IR (neat) cm^{-1} 3068, 2989, 1594, 1474, 1259, 1226, 1172, 1030, 638; ^1H NMR (400 MHz, $CDCl_3$): δ 8.21-8.13 (m, 3H), 7,83-7.70 (m, 5H), 7.46 (dt, J = 8.0 Hz, 2.4 Hz, 1H), 5.45 (sept, J = 6.8 Hz, 1H), 1.53 (d, J = 6.4 Hz, 6H); ^{13}C NMR (100 MHz, CD_3CN): δ 163.6 (d, $^1J_{CF}$ = 251 Hz), 135.7, 134.0 (d, $^3J_{CF}$ = 8.5 Hz), 132.13, 132.07, 128.3 (d, $^4J_{CF}$ = 3.5 Hz), 125.6 (d, $^3J_{CF}$ = 8.0 Hz), 123.4, 122.8 (d, $^2J_{CF}$ = 21.2 Hz), 121.7 (q, $^1J_{CF}$ = 318.9 Hz), 118.9 (d, $^2J_{CF}$ = 25.6 Hz), 51.9, 18.3, 18.2. ^{19}F NMR (376 MHz, CD_3CN): δ -108.1, -79.3; HRMS (ESI) m/z: $[M-OTf]^+$ Calcd for $C_{15}H_{16}FS$ 247.0951; Found 247.0962.

Cyclopropyl-3-fluorophenyl-phenylsulfonium triflate (3m)

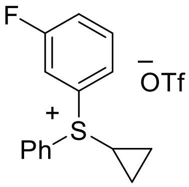

Reaction of cyclopropyl phenyl sulfide **2m** (15 mg, 0.1 mmol) and **1a** (64.1 mg, 0.12 mmol) according to the general procedure afforded 32.2 mg (82%) of product **3m**, isolated as a light yellow oil; IR (neat) cm^{-1} 3120, 3063, 1594, 1479, 1260, 1225, 1157, 1029, 637; ^1H NMR (400 MHz, $CDCl_3$): δ 8.03 (d, J = 6.8 Hz, 2H), 7.94-7.89 (m, 1H), 7.78-7.65 (m, 5H), 7.45-7.38 (m, 1H), 4.03-3.95 (m, 1H), 1.75-1.62 (m, 2H), 1.57-1.44 (m, 2H); ^{13}C NMR (100 MHz, CD_3CN): δ 163.9 (d, $^1J_{CF}$ = 250.9 Hz), 135.5, 134.0 (d, $^3J_{CF}$ = 8.3 Hz), 132.2,

131.1, 128.9 (d, $^3J_{CF}$ = 8.2 Hz), 127.2 (d, $^4J_{CF}$ = 3.4 Hz), 126.6, 122.4 (d, $^2J_{CF}$ = 21.3 Hz), 122.1 (q, $^1J_{CF}$ = 319.2 Hz), 118.2, 23.7, 8.4, 8.2. ^{19}F NMR (376 MHz, $CDCl_3$): δ -105.2, -78.3; HRMS (ESI) m/z: $[M-OTf]^+$ Calcd for $C_{15}H_{14}FS$ 245.0795; Found 245.0807.

3-Fluorophenyl-diphenylsulfonium triflate (3n)

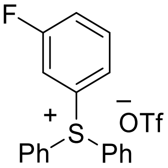

Reaction of diphenyl sulfide **2n** (18.6 mg, 0.1 mmol) and **1a** (64.1 mg, 0.12 mmol) according to the general procedure for 24 hours afforded 42.1 mg (98%) of product **3n**, isolated as a white solid: mp 111.3-112.5 °C; IR (KBr) cm^{-1} 3094, 3067, 3032, 1592, 1477, 1263, 1225, 1150, 1032, 637; ^{1}H NMR (400 MHz, $CDCl_3$): δ 7.85-7.69 (m, 1H), 7.66 (d, J = 8.0 Hz, 1H), 7.47 (dt, J = 8.0 Hz, 2.4 Hz, 1H), 7.35 (dt, J = 7.2 Hz, 2.0 Hz, 1H); ^{13}C NMR (100 MHz, $CDCl_3$): δ 163.3 (d, $^1J_{CF}$ = 255 Hz), 135.0, 133.5 (d, $^3J_{CF}$ = 8.1 Hz), 131.8, 131.3, 127.3 (d, $^4J_{CF}$ = 3.6 Hz), 126.3 (d, $^3J_{CF}$ = 7.9 Hz), 123.8, 122.1 (d, $^2J_{CF}$ = 21 Hz), 120.9 (q, $^1J_{CF}$ = 318.8 Hz), 118.1 (d, $^2J_{CF}$ = 25.7 Hz). ^{19}F NMR (376 MHz, $CDCl_3$): δ -104.8, -78.2; HRMS (ESI) m/z: $[M-OTf]^+$ Calcd for $C_{18}H_{14}FS$ 281.0795; Found 281.0809.

Dibutyl-3-fluorophenylsulfonium triflate (3o)

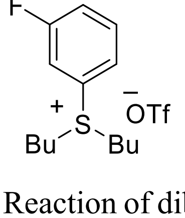

Reaction of dibutyl sulfide **2o** (14.6 mg, 0.1 mmol) and **1a** (64.1 mg, 0.12 mmol) according to the general procedure afforded 36.9 mg (95%) of product **3o**, isolated as a light yellow oil; IR (neat) cm^{-1} 3100, 2967, 2938, 2878, 1594, 1483, 1259, 1225, 1159, 1030, 638; ^{1}H

NMR (400 MHz, $CDCl_3$): δ 7.92 (d, J = 8.4 Hz, 1H), 7.81-7.74 (m, 1H), 7.70-7.64 (m, 1H), 7.52 (dt, J = 8.0 Hz, 2.4 Hz, 1H), 4.02-3.80 (m, 4H), 1.75-1.44 (m, 8H), 0.93 (t, J = 7.2 Hz, 3H); ^{13}C NMR (100 MHz, CD_3CN): δ 163.7 (d, $^1J_{CF}$ = 250.2 Hz), 133.7 (d, $^3J_{CF}$ = 8.4 Hz), 128.8 (d, $^4J_{CF}$ = 3.3 Hz), 123.5 (d, $^3J_{CF}$ = 8.2 Hz), 122.9 (d, $^2J_{CF}$ = 21.1 Hz), 121.7 (q, $^1J_{CF}$ = 318.8 Hz), 118.9 (d, $^2J_{CF}$ = 25.2 Hz), 44.3, 26.6, 21.5, 13.1. ^{19}F NMR (376 MHz, CD_3CN): δ -108.6, -79.2; HRMS (ESI) m/z: $[M-OTf]^+$ Calcd for $C_{14}H_{22}FS$ 241.1421; Found 241.1427.

3-Fluorophenyl-dioctylsulfonium triflate (3p)

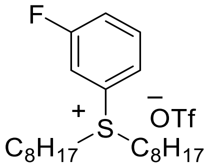

Reaction of dioctyl sulfide **2p** (25.9 mg, 0.1 mmol) and **1a** (64.1 mg, 0.12 mmol) according to the general procedure afforded 50.1 mg (100%) of product **3p**, isolated as a light yellow oil; IR (neat) cm^{-1} 3062, 2930, 2859, 1594, 1482, 1257, 1225, 1160, 1031, 638; ^1H NMR (400 MHz, $CDCl_3$): δ 7.92 (d, J = 7.6 Hz, 1H), 7.81-7.74 (m, 1H), 7.72-7.64 (m, 1H), 7.52 (t, J = 8.2 Hz, 1H), 4.00-3.81 (m, 4H), 1.73-1.14 (m, 24H), 0.86 (t, J = 6.2 Hz, 3H); ^{13}C NMR (100 MHz, CD_3CN): δ 163.7 (d, $^1J_{CF}$ = 250.2 Hz), 133.7 (d, $^3J_{CF}$ = 8.4 Hz), 128.8 (d, $^4J_{CF}$ = 3.3 Hz), 123.5 (d, $^3J_{CF}$ = 8.3 Hz), 122.9 (d, $^2J_{CF}$ = 21.2 Hz), 121.7 (q, $^1J_{CF}$ = 318.9 Hz), 118.9 (d, $^2J_{CF}$ = 25.3 Hz), 44.6, 31.9, 29.1, 28.9, 28.1, 24.6, 22.9, 13.9. ^{19}F NMR (376 MHz, CD_3CN): δ -108.6, -79.2; HRMS (ESI) m/z: $[M-OTf]^+$ Calcd for $C_{22}H_{38}FS$ 353.2673; Found 353.2683.

1-(3-Fluorophenyl)-tetrahydro-1*H*-1-thiophenium triflate (3q)

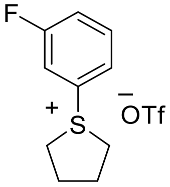

Reaction of tetrahydrothiophene **2q** (8.8 mg, 0.1 mmol) and **1a** (64.1 mg, 0.12 mmol) according to the general procedure afforded 25 mg (75%) of product **3q**, isolated as a white solid: mp 72.2-73.0 °C; IR (KBr) cm^{-1} 3076, 3031, 3004, 2975, 2957, 2891, 1596, 1484, 1260, 1222, 1167, 1029, 636; ^1H NMR (400 MHz, CD_3CN): δ 7.85-7.58 (m, 3H), 7.51 (t, J = 8.4 Hz, 1H), 4.00-3.88 (m, 2H), 3.80-3.63 (m, 2H), 2.58-2.29 (m, 4H); ^{13}C NMR (100 MHz, CD_3CN): δ 163.4 (d, $^1J_{CF}$ = 249.6 Hz), 133.4 (d, $^3J_{CF}$ = 8.3 Hz), 128.7 (d, $^3J_{CF}$ = 8.2 Hz), 126.9 (d, $^4J_{CF}$ = 3.5 Hz), 121.6 (d, $^2J_{CF}$ = 21.2 Hz), 121.7 (q, $^1J_{CF}$ = 318.5 Hz), 117.6, 49.0, 29.4. ^{19}F NMR (376 MHz, CD_3CN): δ -108.4, -79.3; HRMS (ESI) m/z: $[M-OTf]^+$ Calcd for $C_{10}H_{12}FS$ 183.0638; Found 183.0652.

1-(3-Fluorophenyl)-hexahydrothiopyrylium triflate (3r)

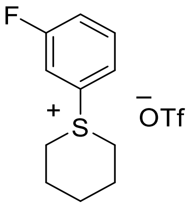

Reaction of tetrahydro-2*H*-thiopyran **2r** (10.2 mg, 0.1 mmol) and **1a** (64.1 mg, 0.12 mmol) according to the general procedure afforded 27.2 mg (79%) of product **3r**, isolated as a white solid: mp 76.5-77.9 °C; IR (KBr) cm^{-1} 3086, 3074, 3007, 2958, 2913, 1596, 1482, 1261, 1225, 1154, 1029, 637; ^1H NMR (400 MHz, $CDCl_3$): δ 7.98 (d, J = 8.4 Hz, 1H), 7.81-7.66 (m, 2H), 7.45 (t, J = 8.4 Hz, 1H), 4.06 (t, J = 11.4 Hz, 1H), 3.84-3.72 (m, 2H), 2.48-2.34 (m, 2H), 2.12-1.92 (m, 4H); ^{13}C NMR (100 MHz, CD_3CN): δ 163.5 (d, $^1J_{CF}$ = 249.6 Hz), 133.5 (d, $^3J_{CF}$ = 8.4 Hz), 126.9 (d, $^4J_{CF}$ = 3.4 Hz), 126.0 (d, $^3J_{CF}$ = 8.2 Hz), 122.0 (d, $^2J_{CF}$ = 21.0 Hz), 121.7 (q, $^1J_{CF}$ = 318.8 Hz), 117.6 (d, $^2J_{CF}$ = 25.8 Hz), 41.2, 22.8, 22.7.

^{19}F NMR (376 MHz, CD_3CN): δ -109.2, -79.3; HRMS (ESI) m/z: $[M\text{-}OTf]^+$ Calcd for $C_{11}H_{14}FS$ 197.0795; Found 197.0812.

1-(3-Fluorophenyl)-1,4-dithian-1-ium triflate (3s)

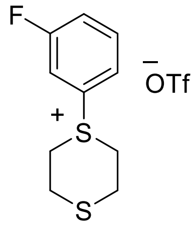

Reaction of 1,4-dithiane **2s** (12.0 mg, 0.1 mmol) and **1a** (64.1 mg, 0.12 mmol) according to the general procedure for 24 hours afforded 18.8 mg (52%) of product **3s**, isolated as a white solid: mp 98.0-99.2 °C; IR (KBr) cm^{-1} 3064, 3001, 2944, 1588, 1478, 1265, 1223, 1143, 1028, 637; ^1H NMR (400 MHz, $CDCl_3$): δ 7.86 (d, J = 8.0 Hz, 2H), 7.76-7.68 (m, 2H), 7.48 (t, J = 8.6 Hz, 1H), 4.33-4.25 (m, 2H), 3.86-3.75 (m, 2H), 3.60-3.49 (m, 2H), 3.24-3.15 (m, 2H); ^{13}C NMR (100 MHz, CD_3CN): δ 163.5 (d, $^1J_{CF}$ = 249.9 Hz), 133.6 (d, $^3J_{CF}$ = 8.5 Hz), 127.3 (d, $^4J_{CF}$ = 3.4 Hz), 124.9 (d, $^3J_{CF}$ = 8.3 Hz), 122.5 (d, $^2J_{CF}$ = 21.1 Hz), 121.7 (q, $^1J_{CF}$ = 318.7 Hz), 118.2, 41.9, 25.1. ^{19}F NMR (376 MHz, CD_3CN): δ -108.9, -79.3; HRMS (ESI) m/z: $[M\text{-}OTf]^+$ Calcd for $C_{10}H_{12}FS_2$ 215.0359; Found 215.0371.

4-(3-Fluorophenyl)-1,4-oxathian-4-ium triflate (3t)

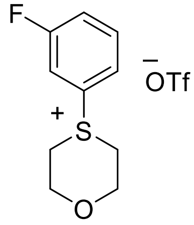

Reaction of 1,4-oxathiane **2t** (10.4 mg, 0.1 mmol) and **1a** (64.1 mg, 0.12 mmol) according to the general procedure for 24 hours afforded 23 mg (66%) of product **3t**, isolated as a

white solid: mp 121.2-123.1 °C; IR (KBr) cm^{-1} 3080, 3066, 3015, 2954, 2887, 1587, 1478, 1264, 1223, 1162, 1152, 1032, 642; ^{1}H NMR (400 MHz, $CDCl_3$): δ 7.83 (d, J = 8.0 Hz, 1H), 7.76-7.63 (m, 2H), 7.47 (t, J = 8.0 Hz, 1H), 4.41 (d, J = 14.0 Hz, 2H), 4.24-4.13 (m, 2H), 4.12-4.02 (m, 2H), 3.68-3.56 (m, 2H); ^{13}C NMR (100 MHz, CD_3CN): δ 163.6 (d, $^{1}J_{CF}$ = 249.9 Hz), 133.6 (d, $^{3}J_{CF}$ = 8.3 Hz), 127.0 (d, $^{4}J_{CF}$ = 3.5 Hz), 124.8 (d, $^{3}J_{CF}$ = 8.3 Hz), 122.1 (d, $^{2}J_{CF}$ = 21 Hz), 121.7 (q, $^{1}J_{CF}$ = 318.8 Hz), 117.6, 63.7, 38.5. ^{19}F NMR (376 MHz, CD_3CN): δ -108.9, -79.3; HRMS (ESI) m/z: [M-OTf]$^{+}$ Calcd for $C_{10}H_{12}FSO$ 199.0587; Found 199.0604.

5-(3-Fluorophenyl)-5*H*-dibenzo[*b,d*]thiophen-5-ium triflate (3u)

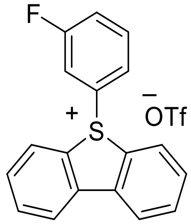

Reaction of dibenzothiophene **2u** (18.4 mg, 0.1 mmol) and **1a** (106.8 mg, 0.2 mmol) according to the general procedure afforded 33.5 mg (80%) of product **3u**, isolated as a white solid: mp 169.8-170.5 °C; IR (KBr) cm^{-1} 3099, 3073, 3038, 1595, 1480, 1255, 1222, 1155, 1029, 637; ^{1}H NMR (400 MHz, CD_3CN): δ 8.35 (d, J = 8.0 Hz, 2H), 8.12 (d, J = 8.0 Hz, 2H), 7.97 (d, J = 8.0 Hz, 2H), 7.74 (d, J = 8.0 Hz, 2H), 7.67-7.59 (m, 1H), 7.54-7.45 (m, 2H), 7.40-7.34 (m, 1H); ^{13}C NMR (100 MHz, CD_3CN): δ 163.5 (d, $^{1}J_{CF}$ = 251.6 Hz), 140.2, 135.3, 134.1 (d, $^{3}J_{CF}$ = 7.5 Hz), 132.4, 131.9 (d, $^{4}J_{CF}$ = 2.1 Hz), 129.1 (d, $^{3}J_{CF}$ = 7.3 Hz), 128.7, 127.4, 125.3, 123.0 (d, $^{2}J_{CF}$ = 21.1 Hz), 121.5 (q, $^{1}J_{CF}$ = 319.3 Hz), 118.1. ^{19}F NMR (376 MHz, CD_3CN): δ -107.9, -79.3; HRMS (ESI) m/z: [M-OTf]$^{+}$ Calcd for $C_{18}H_{12}FS$ 279.0638; Found 279.0649.

4-Fluoro-1-phenyl-2,3-dihydro-1*H*-benzo[*b*]thiophen-1-ium triflate (3v)

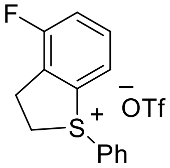

Reaction of 2-chloroethyl phenyl sulfide **2v** (17.3 mg, 0.1 mmol) and **1a** (64.1 mg, 0.12 mmol) according to the general procedure afforded 16.1 mg (42%) of product **3v**, isolated as a whitish oil; IR (neat) cm^{-1} 3063, 2959, 2940, 2850, 1594, 1479, 1260, 1225, 1157, 1030, 638; ^{1}H NMR (400 MHz, $CDCl_3$): δ 7.92-7.86 (m, 2H), 7.84-7.70 (m, 5H), 7.57 (dd, J = 16.0 Hz, 8.8 Hz, 1H), 7.50-7.43 (m, 2H), 6.77 (dd, J = 8.8 Hz, 2.4 Hz, 1H), 6.55 (dd, J = 16.0 Hz, 2.4 Hz, 1H); ^{13}C NMR (100 MHz, $CDCl_3$): δ 163.1 (d, $^{1}J_{CF}$ = 254.6 Hz), 139.0, 135.0, 133.4 (d, $^{3}J_{CF}$ = 7.9 Hz), 131.8, 130.8, 127.1 (d, $^{3}J_{CF}$ = 8.0 Hz), 126.4 (d, $^{4}J_{CF}$ = 3.6 Hz), 124.6, 122.8, 122.0 (d, $^{2}J_{CF}$ = 21 Hz), 121.0 (q, $^{1}J_{CF}$ = 318.4 Hz), 117.5 (d, $^{2}J_{CF}$ = 25.6 Hz).; ^{19}F NMR (376 MHz, $CDCl_3$): δ -104.9, -78.3; HRMS (ESI) m/z: [M-OTf]$^{+}$ Calcd for $C_{14}H_{12}FS$ 231.0638; Found 231.0642.

3-Fluorophenyl-diphenylselenonium triflate (3w)

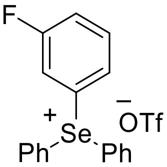

Reaction of diphenyl sulfide **2w** (23.3 mg, 0.1 mmol) and **1a** (64.1 mg, 0.12 mmol) according to the general procedure afforded 47 mg (98%) of product **3w**, isolated as a dark yellow oil; IR (neat) cm^{-1} 3100, 3067, 1592, 1477, 1258, 1224, 1155, 1030, 636; ^{1}H NMR (400 MHz, $CDCl_3$): δ 7.78-7.70 (m, 2H), 7.70-7.55 (m, 10H), 7.41 (t, J = 8.2 Hz, 1H), 7.25-7.22 (m, 1H).; ^{13}C NMR (100 MHz, CD_3CN): δ 163.7 (d, $^{1}J_{CF}$ = 251.3 Hz), 134.4, 133.9 (d, $^{3}J_{CF}$ = 8.0 Hz), 132.2, 132.0, 128.5 (d, $^{3}J_{CF}$ = 7.6 Hz), 128.1 (d, $^{4}J_{CF}$ = 3.3 Hz), 127.0,

121.5 (d, $^2J_{CF}$ = 21.2 Hz), 121.7 (q, $^1J_{CF}$ = 318.9 Hz), 119.1 (d, $^2J_{CF}$ = 25.6 Hz).; ^{19}F NMR (376 MHz, CD_3CN): δ -108.3, -79.2; HRMS (ESI) m/z: [M-OTf]$^+$ Calcd for $C_{18}H_{14}FSe$ 329.0239; Found 329.0250.

4. Reaction of mesityl-1-phenyboronic acid-2-iodonium triflate 1b with thioanisole 2a.

Methyl-diphenylsulfonium triflate (4)[4]

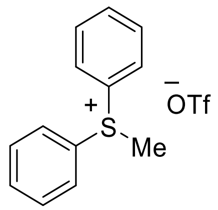

Reaction of thioanisole **2a** (12.4 mg, 0.1 mmol) was added to a solution of mesityl-1-phenyboronic acid-2-iodonium triflate **1b** (61.9 mg, 0.12 mmol) in dichloromethane (0.9 mL) and sat. $NaHCO_3$ (0.1 mL). The reaction was stirred at room temperature for 3 hours. After reaction, water (5 mL) was added, and the mixture was extracted with dichloromethane. The organic layer was dried over by anhydrous Na_2SO_4 and concentrated under reduced pressure. The crude mixture was washed with diethyl ether several times and then dried in vacuum to give the analytically pure sulfonium triflate **4**. Isolated as a white solid: mp 95.1-96.8 °C (lit.[4] mp 95-96.5); IR (KBr) cm^{-1} 3098, 3077, 3024, 2938, 1587, 1484, 1255, 1222, 1158, 1029, 635; ^{1}H NMR (400 MHz, $CDCl_3$): δ 7.92 (d, J = 7.2 Hz, 4H), 7.77-7.60 (m, 6H), 3.73 (s, 3H); ^{13}C NMR (100 MHz, CD_3CN): δ 134.9, 131.8, 130.5, 126.8, 121.7 (q, $^1J_{CF}$ = 318.8 Hz), 28.1. ^{19}F NMR (376 MHz, CD_3CN): δ -79.3; HRMS (ESI) m/z: [M-OTf]$^+$ Calcd for $C_{13}H_{13}S$ 201.0732; Found 201.0731.

5. *Control experiments*

Reaction in CD_2Cl_2:

Diphenyl sulfide **2n** (0.1 mmol, 18.6 mg) was added to a solution of mesityle-2-fluoro-1-phenylboronic acid-iodonium triflate **1a** (0.12 mmol, 64.1 mg) in deuterated dichloromethane (0.9 mL) and water (0.1 mL). The reaction was stirred at room temperature for 24 hours. After reaction, water (5 mL) was added, and the mixture was extracted with dichloromethane. The organic layer was dried over by anhydrous Na_2SO_4 and concentrated under reduced pressure. The crude mixture was washed with diethyl ether several times and then dried in vacuum to give 41.4 mg (96%) of the 3-fluorophenyl-diphenylsulfonium triflate **(3n),** isolated as a white solid.

Reaction with D_2O:

3-Fluorophenyl-2-*d*-diphenylsulfonium triflate (5)

Diphenyl sulfide **2n** (0.1 mmol, 18.6 mg) was added to a solution of mesityl-2-fluoro-1-phenylboronic acid-iodonium triflate **1a** (0.12 mmol, 64.1 mg) in dichloromethane (0.9 mL) and deuterated water (0.1 mL). The reaction was stirred at room temperature for 24 hours. After reaction, water (5 mL) was added, and the mixture was extracted with dichloromethane. The organic layer was dried over by anhydrous Na_2SO_4 and concentrated under reduced pressure. The crude mixture was washed with diethyl ether several times and then dried in vacuum to give 40.6 mg (94%) of the deuterated product **5** (>99% D), isolated as a white solid: mp 113.0-113.5 °C; IR (KBr) cm^{-1} 3163, 3074, 3025, 1584, 1456, 1269, 1151, 1030, 635; ^{1}H NMR (400 MHz, $CDCl_3$): δ 7.85-7.71 (m, 11H), 7.65 (d, J = 8.0 Hz, 1H), 7.49 (t, J = 8.0 Hz, 1H).; ^{13}C NMR (100 MHz, CD_3CN): δ 163.2 (d, $^1J_{CF}$ = 254.9 Hz), 135.0, 133.5 (d, $^3J_{CF}$ = 8.2 Hz), 131.8, 131.3, 127.3 (d, $^4J_{CF}$ = 3.5 Hz), 126.2 (d, $^3J_{CF}$ = 7.6 Hz), 123.8, 122.1 (d, $^2J_{CF}$ = 20.7 Hz), 120.9 (q, $^1J_{CF}$ = 318.8 Hz), 117.9 (d, $^2J_{CF}$ = 25.1 Hz); ^{19}F NMR (376 MHz, $CDCl_3$): δ -105.0, -78.2.; HRMS (ESI) m/z: $[M-OTf]^+$ Calcd for $C_{18}H_{13}DFS$ 282.0858; Found 282.0875.

6. Reaction of mesityle-2-fluoro-1-phenylboronic acid-6-iodonium triflate with dimethylsulfoxide or methyl phenyl sulfoxide

Sulfoxide **6** (0.1 mmol, 1 equiv.) was added to a solution of mesityle-2-fluoro-1-phenylboronic acid-iodonium triflate **1a** (0.12 mmol, 1.2 equiv.) in dichloromethane (0.9 mL) and water (0.1 mL). The reaction was stirred at room temperature for 3 hours. After reaction, the mixture was concentrated under reduced pressure. The crude mixture was washed with diethyl ether several times and then dried in vacuum to give the analytically pure sulfonium triflate **7**.

2-Fluoro-6-hydroxyphenyl-dimethylsulfonium triflate (7a)

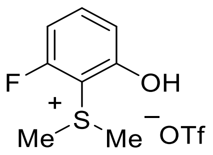

Reaction of dimethylsulfoxide **6a** (7.8 mg, 0.1 mmol) and **1a** (64.1 mg, 0.12 mmol) according to the general procedure afforded 22.6 mg (71%) of product **7a**, isolated as a white solid: mp 64.0-65.1 °C ; IR (neat) cm^{-1} 3450, 3125, 2913, 1604, 1478, 1279, 1175, 644; ^{1}H NMR (400 MHz, $CDCl_3$): δ 9.64-9.34 (brs, 1H), 7.63 (td, J = 8.4 Hz, 6.4 Hz, 1H), 7.00 (d, J = 8.8 Hz, 1H), 6.95-6.89 (m, 1H), 3.24 (s, 6H); ^{13}C NMR (100 MHz, CD_3CN): δ 163.1 (d, $^{1}J_{CF}$ = 252.7 Hz), 160.9, 138.6 (d, $^{3}J_{CF}$ = 11.2 Hz), 121.6 (q, $^{1}J_{CF}$ = 318.2 Hz), 114.3 (d, $^{4}J_{CF}$ = 3.3 Hz), 108.5 (d, $^{2}J_{CF}$ = 21.0 Hz), 97.3 (d, $^{2}J_{CF}$ = 15.7 Hz), 26.5 (d, $^{4}J_{CF}$ = 1.5 Hz).; ^{19}F NMR (376 MHz, $CDCl_3$): δ -107.0, -79.4; HRMS (ESI) m/z: $[M-OTf]^{+}$ Calcd for $C_8H_{10}FOS$ 173.0431; Found: 173.0435.

Crystal structure determination of compound **7a**:

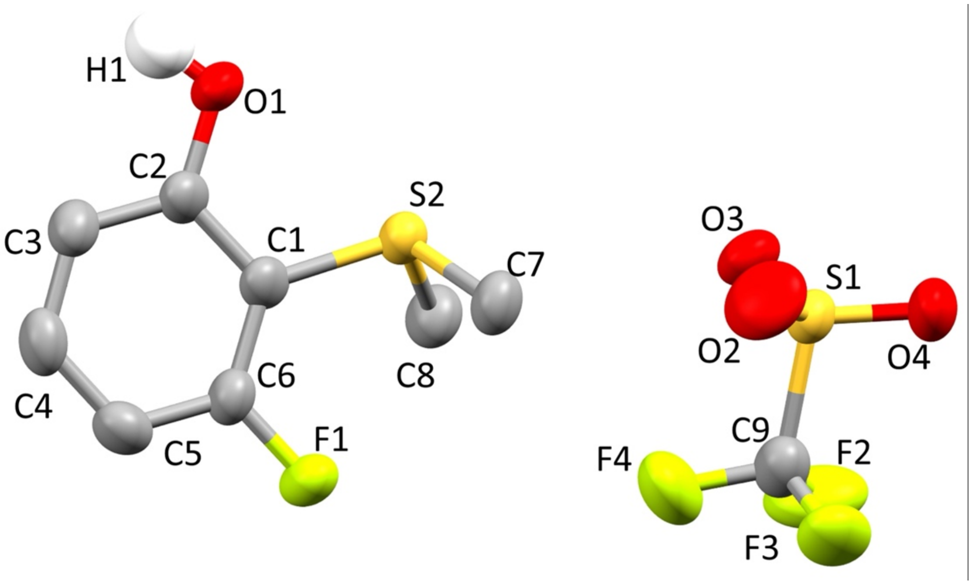

Figure S2: Thermal ellipsoid plot of **7a** drawn 50% probability level. Non-oxygen atom hydrogen atoms were removed for clarity.

Single crystals of product **7a** suitable for X-ray crystallographic analysis were obtained by slow crystallization from acetonitrile solution. X-ray diffraction data for **7a** were collected on Rigaku RAPID II Image Plate system using graphite-monochromated Cu Kα radiation (λ = 1.54187 Å) at 173 K. The structure was solved by Superflip[2] and refined using SHELXL-2014/7.[3] The hydrogen atom (H1) was located on a difference map and constrained geometrically (AFIX 147). Crystal data for **7a** $C_9H_{10}F_4O_4S_2$, M = 322.29, triclinic, space group P-1, a = 8.0986(2) Å, b = 8.1925(2) Å, c = 11.1915(8) Å, α = 101.113(7) °, β = 101.677(7) °, γ = 113.360(8) °, V = 636.04(7) Å^3, Z = 2, 5509 reflections measured, 2196 unique reflections (R_{int} = 0.0567), 1414 I > 2/s(I), 176 parameters, 0 restraints; GooF = 1.170, final R1 = 0.0626, wR (all) = 0.0975. CCDC 2312445.

2-Fluoro-6-hydroxyphenyl-methyl-phenylsulfonium triflate (7b)

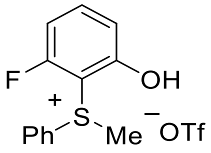

Reaction of methyl phenyl sulfoxide **6b** (14 mg, 0.1 mmol) and **1a** (64.1 mg, 0.12 mmol) according to the general procedure afforded 24.4 mg (64%) of product **7b**, isolated as a light yellow oil ; IR (neat) cm^{-1} 3063, 2938,2875, 2825, 1608, 1469, 1235, 1161, 1029, 637; ^{1}H NMR (400 MHz, $CDCl_3$): δ 7.89 (d, J = 7.6 Hz, 2H), 7.76-7.61 (m, 3H), 7.59-7.49 (m, 1H), 7.20 (d, J = 8.4 Hz, 1H), 6.75 (t, J = 9.2 Hz, 1H), 3.77 (s, 3H).; ^{13}C NMR (100 MHz, CD_3CN): δ 163.2 (d, $^{1}J_{CF}$ = 252.9 Hz), 161.4, 138.9 (d, $^{3}J_{CF}$ = 11.6 Hz), 134.0, 131.3, 129.3, 126.2, 121.6 (q, $^{1}J_{CF}$ = 318.5 Hz), 114.7 (d, $^{4}J_{CF}$ = 3.1 Hz), 108.0 (d, $^{2}J_{CF}$ = 21 Hz), 98.1 (d, $^{2}J_{CF}$ = 15.6 Hz), 25.6 (d, $^{4}J_{CF}$ = 3.4 Hz).; ^{19}F NMR (376 MHz, CD_3CN): δ -107.9, -79.3; HRMS (ESI) m/z: $[M\text{-}OTf]^+$ Calcd for $C_{13}H_{12}FOS$ 235.0587; Found 235.0604.

7. Hammett plot experiments

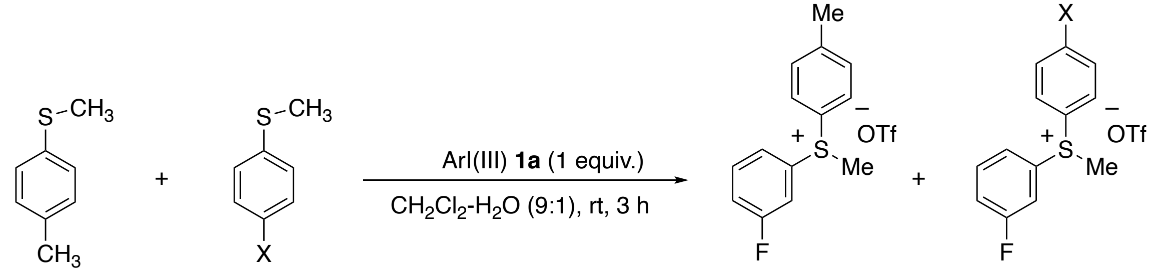

2b (5 equiv.) **2a, 2c, 2h, 2i** (5 equiv.) **3b** **3a, 3c, 3h, 3i**

p-X	σ_p	k_X/k_H
Me	-0.17	1.1
H	0	1.0
Cl	0.23	0.792
CHO	0.42	0.55
NO_2	0.78	0.275

ρ = -0.65 (r = 0.98)

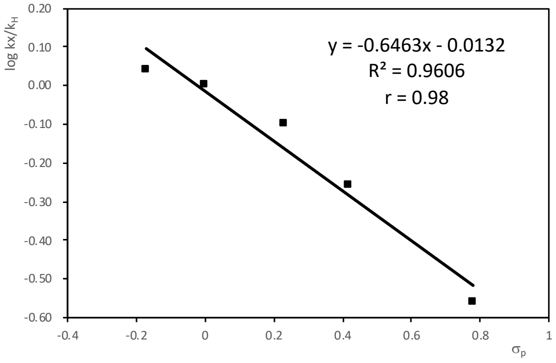

Methyl *p*-tolyl sulfide **2b** (0.5 mmol) and appropriate thioanisole **2** (0.5 mmol) were added to a solution of mesityle-2-fluoro-1-phenylboronic acid-iodonium triflate **1a** (0.1 mmol) in

dichloromethane (0.9 mL) and water (0.1 mL). The reaction mixture was stirred at room temperature for 3 hours. After reaction, the mixture was concentrated under reduced pressure. The crude mixture was checked by ^{1}H NMR. The ^{1}H NMR spectra of the *para*-methyl compound **3b** and the control compound **3** was used to measure the ratio of the reaction rates. Each measurement experiment was performed twice, and the average value was used. The values of σ_p are based on literature values.[5]

8. Reactions of 3-fluorophenyl-methyl-phenylsulfonium triflate with nucleophiles

Methyl phenyl sulfone (11)[6]

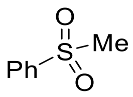

3-Fluorophenyl-methyl-phenylsulfonium triflate **3a** (0.18 mmol, 66.3 mg) was added to a solution of sodium benzenesulfonate (0.15 mmol, 24.6 mg) in acetonitrile (1.5 mL). The reaction was refluxed for 20 hours. After reaction, water (5 mL) was added, and the mixture was extracted with ethyl acetate. The organic layer was dried over anhydrous Na_2SO_4 and concentrated under reduced pressure. Purification by preparative TLC (hexane-ethyl acetate = 2:1) afforded the analytically pure methyl phenyl sulfone **11**; 16.5 mg (71%) isolated as a white solid; ^{1}H NMR (400 MHz, $CDCl_3$): δ 7.98-7.94 (m, 2H), 7.70-7.64 (m, 1H), 7.62-7.55 (m, 2H), 3.06 (s, 3H).

1-Methyl-4-(methylsulfonyl)benzene (12)[7]

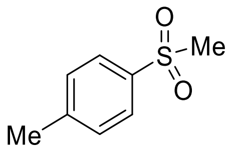

3-Fluorophenyl-methyl-phenylsulfonium triflate **3a** (0.18 mmol, 66.3 mg) was added to a solution of sodium *p*-toluenesulfinate (0.15 mmol, 26.7 mg) in acetonitrile (1.5 mL). The

reaction was refluxed for 20 hours. After reaction, water (5 mL) was added, and the mixture was extracted with ethyl acetate. The organic layer was dried over anhydrous Na_2SO_4 and concentrated under reduced pressure. Purification by preparative TLC (hexane-ethyl acetate = 2:1) afforded the analytically pure 1-methyl-4-(methylsulfonyl)benzene **12**; 13.8 mg (54%) isolated as a white solid; ^{1}H NMR (400 MHz, $CDCl_3$): δ 7.83 (d, *J* = 8.4 Hz, 2H), 7.37 (d, *J* = 8.0 Hz, 2H), 3.04 (s, 3H), 2.46 (s, 3H); ^{13}C NMR (100 MHz, $CDCl_3$): δ 144.7, 137.8, 130.0, 127.4, 44.6, 21.6.

Methyl benzoate (13)[8]

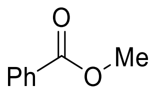

3-Fluorophenyl-methyl-phenylsulfonium triflate **3a** (0.15 mmol, 55.3 mg) was added to a solution of benzoic acid (0.18 mmol, 22 mg) and potassium carbonate (0.18 mmol, 25 mg) in acetonitrile (1.5 mL). The reaction was refluxed for 24 hours. After reaction, water (5 mL) was added, and the mixture was extracted with dichloromethane. The organic layer was dried over anhydrous Na_2SO_4 and concentrated under reduced pressure. Purification by preparative TLC (hexane-ethyl acetate = 99:1) afforded the analytically pure methyl benzoate **13**; 14 mg (69%) isolated as a colorless oil; ^{1}H NMR (400 MHz, $CDCl_3$): δ 8.04 (d, *J* = 6.8 Hz, 2H), 7.61-7.52 (m, 1H), 7.48-7.38 (m, 2H), 3.92 (s, 3H); ^{13}C NMR (100 MHz, $CDCl_3$): δ 167.1, 132.9, 130.2, 129.6, 128.4, 52.1.

9. References

1. (a) V. N. Nemykin, A. V. Maskaev, M. R. Geraskina, M. S. Yusubov and V. V. Zhdankin, *Inorg. Chem.* , 2011, **50**, 11263-11272. (b) A. Yoshimura, J. M. Fuchs, K. R. Middleton, A. V. Maskaev, G. T. Rohde, A. Saito, P. S. Postnikov, M. S. Yusubov, V. N. Nemykin and V. V. Zhdankin, *Chem. - Eur. J.*, 2017, **23**, 16738-16742.
2. L. Palatinus and G. Chapuis, *J. Appl. Crystallogr.*, 2007, **40**, 786-790.
3. G. M. Sheldrick, *Acta Crystallogr., Sect. A: Found. Crystallogr.*, 2008, **64**, 112-122.
4. P. Wyatt, A. Hudson, J. Charmant, A. G. Orpen and H. Phetmung, *Org. Biomol. Chem.*,

2006, **4**, 2218-2232.

5. C. Hansch, A. Leo and R. W. Taft, *Chem. Rev.*, 1991, **91**, 165-195.
6. N. Kennedy, G. Lu, P. Liu and T. Cohen, *J. Org. Chem.*, 2015, **80**, 8571-8582.
7. A. Shavnya, S. B. Coffey, A. C. Smith and V. Mascitti, *Org. Lett.*, 2013, **15**, 6226-6229.
8. Q. Xia, X. Liu, Y. Zhang, C. Chen and W. Chen, *Org. Lett.*, 2013, **15**, 3326-332

10. NMR Spectra of Products

^{1}H NMR (400 MHz, CD_3CN); **1a**

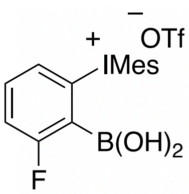

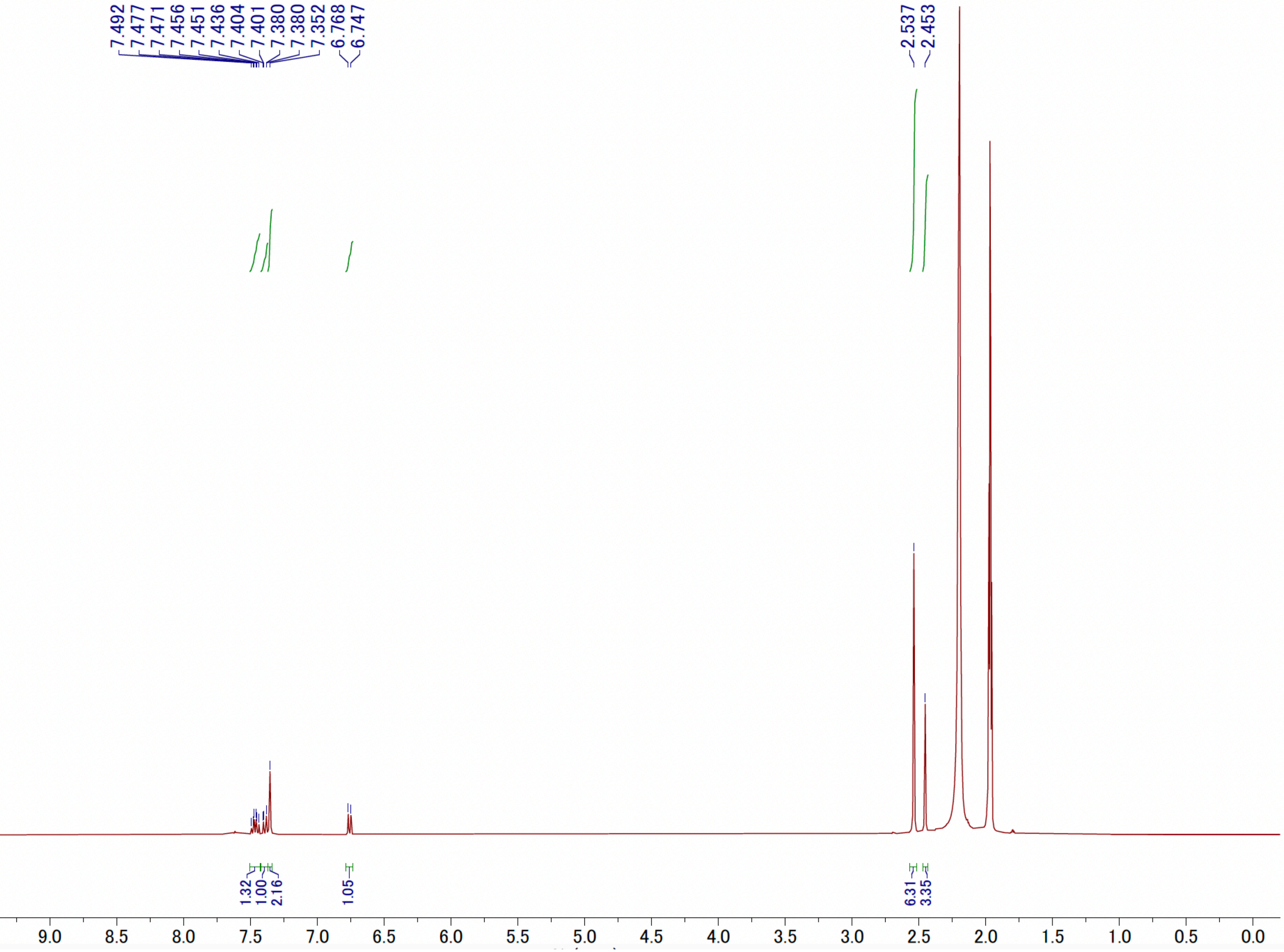

^{1}H NMR (400 MHz, CDCl$_3$) ; **3a**

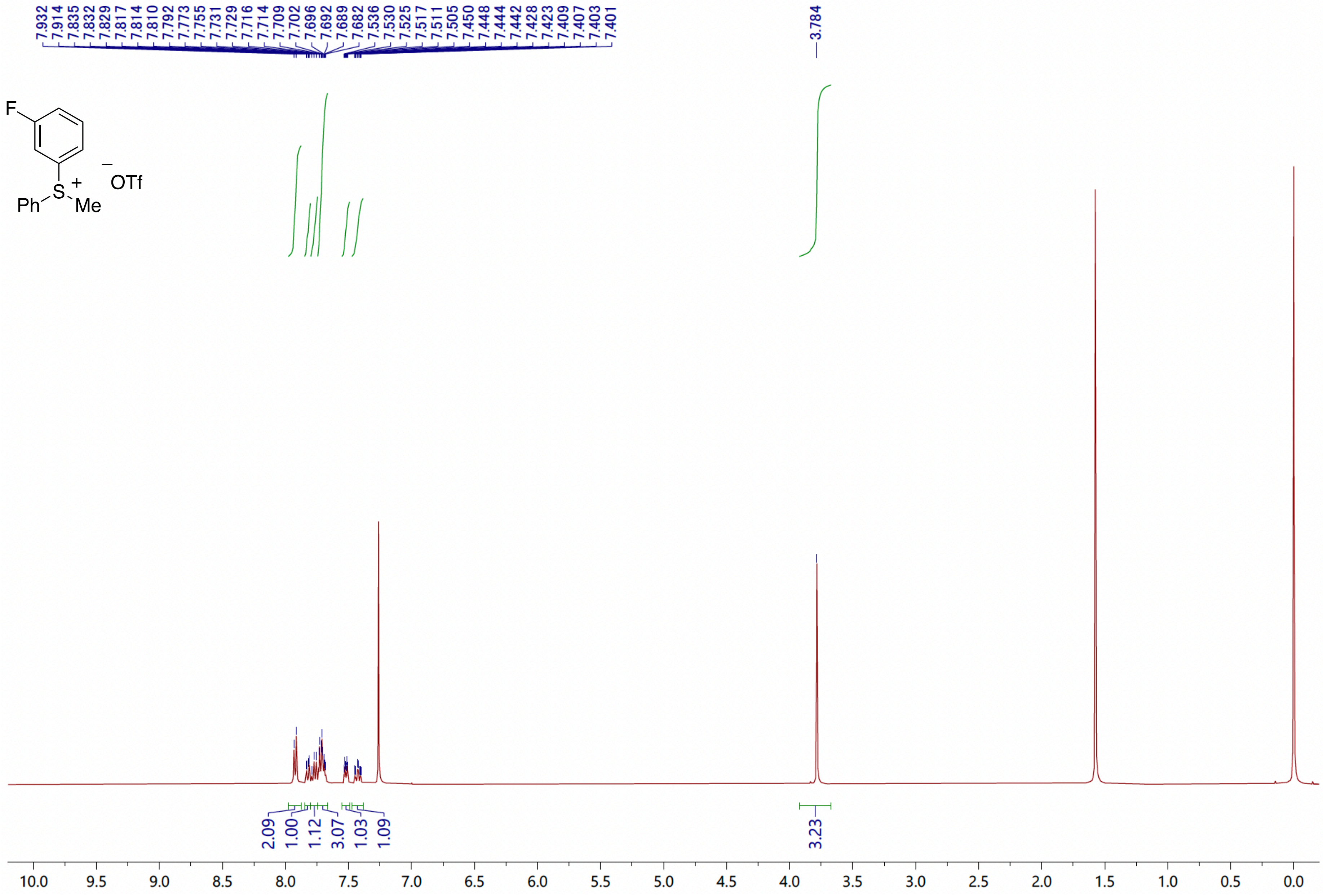

^{13}C NMR (100 MHz, CD_3CN) ; **3a**

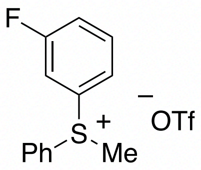

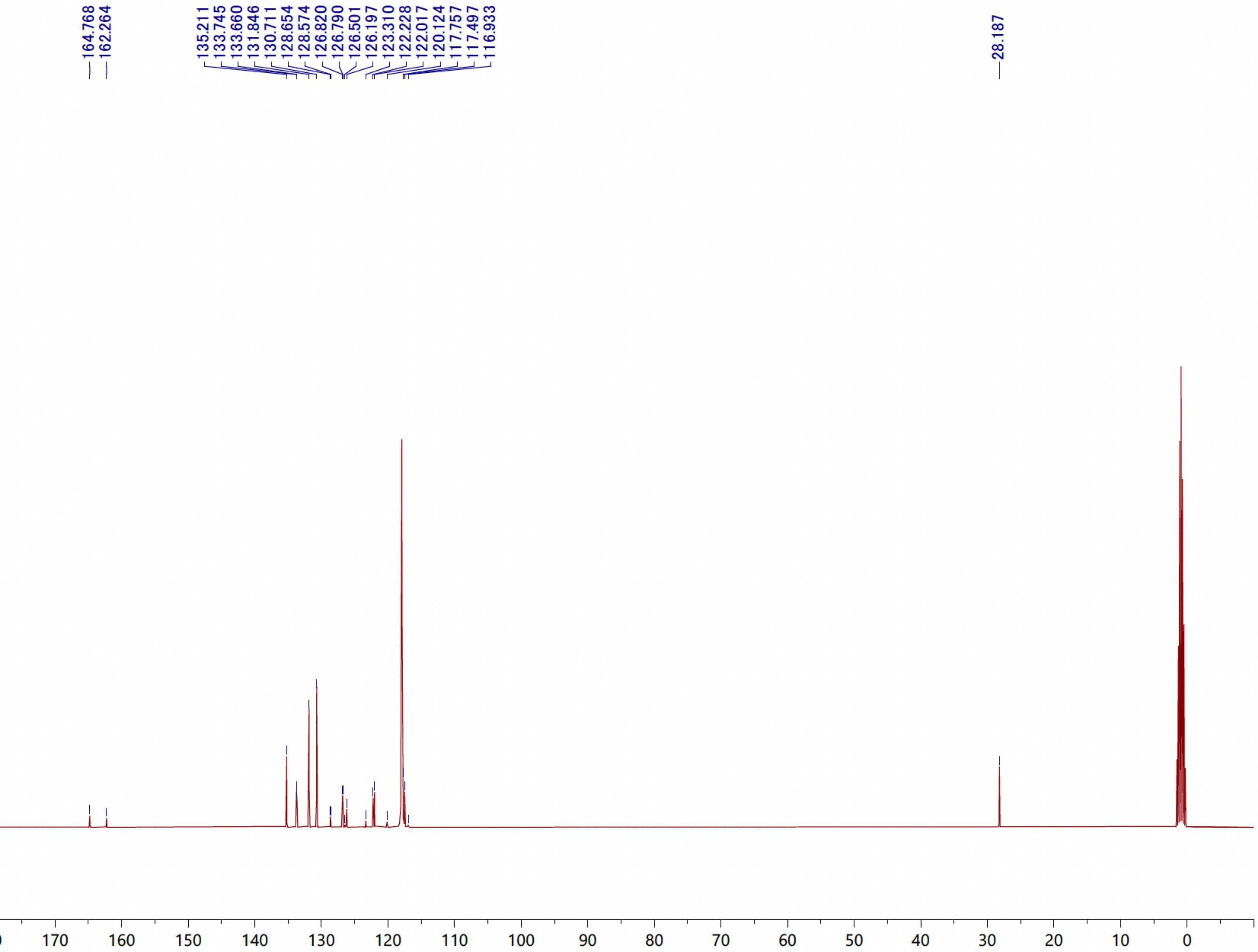

^{19}F NMR (376 MHz, CD_3CN) ; **3a**

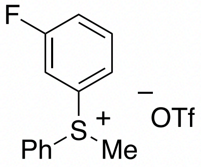

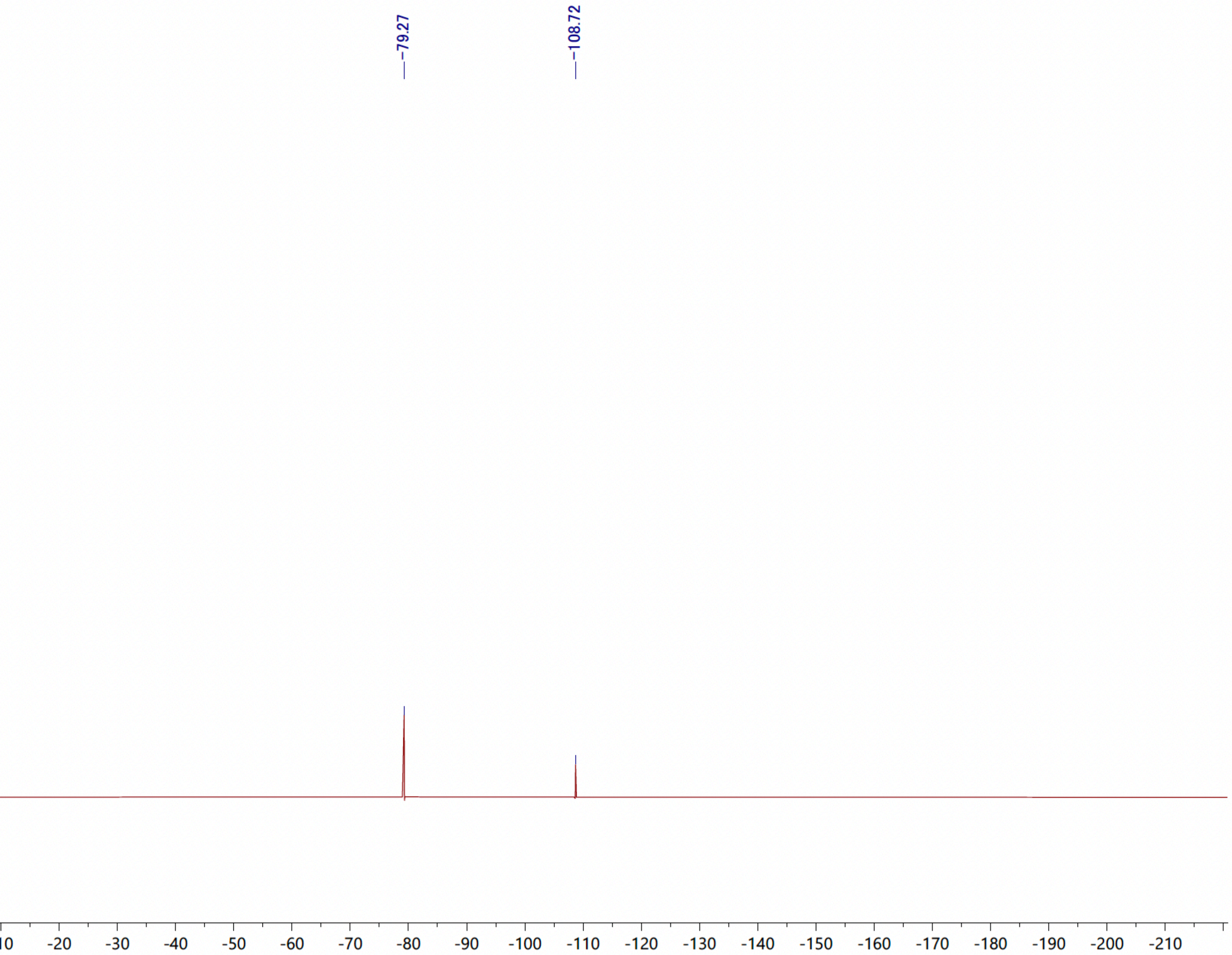

^{1}H NMR (400 MHz, $CDCl_3$) ; **3b**

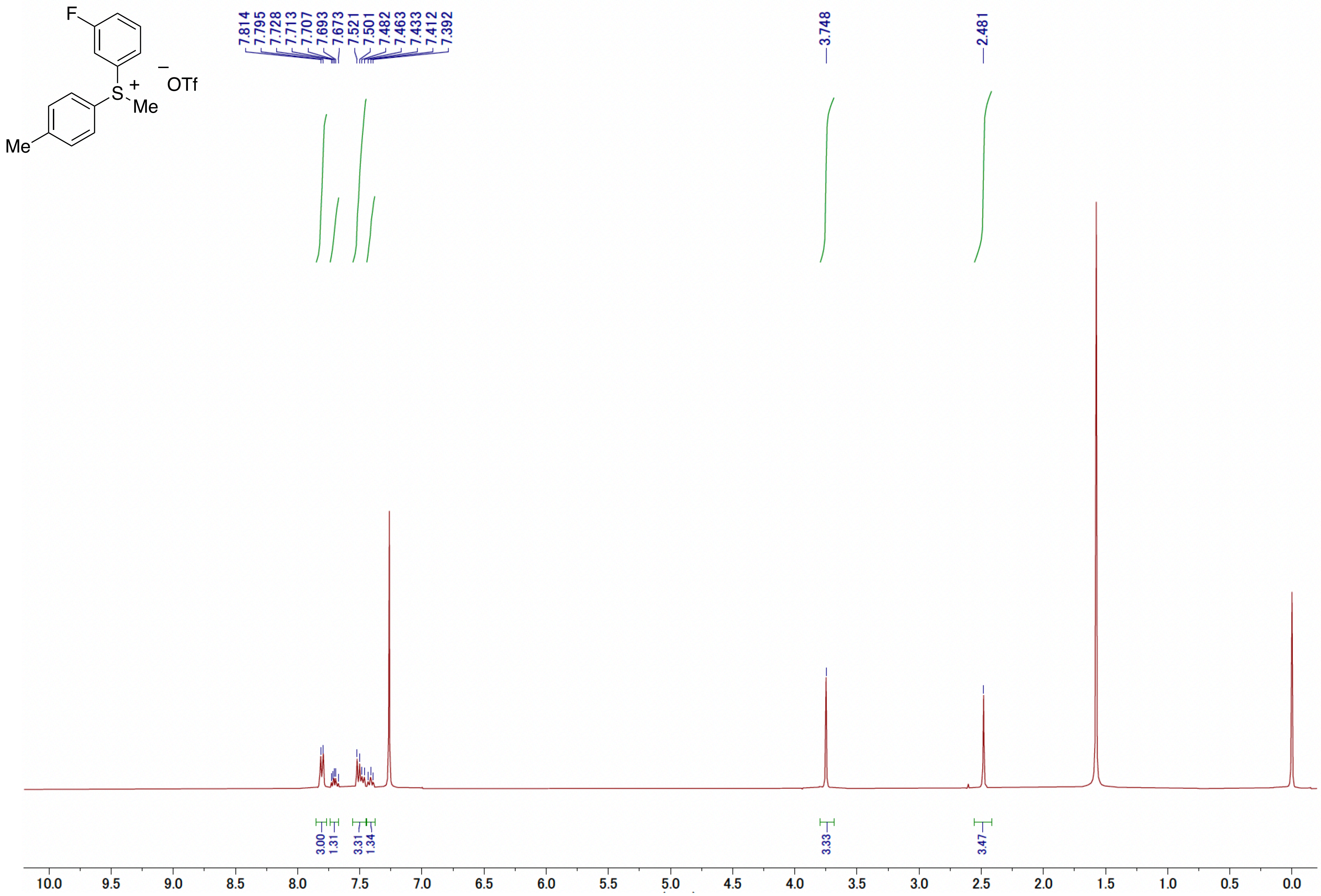

^{13}C NMR (100 MHz, CD_3CN) ; **3b**

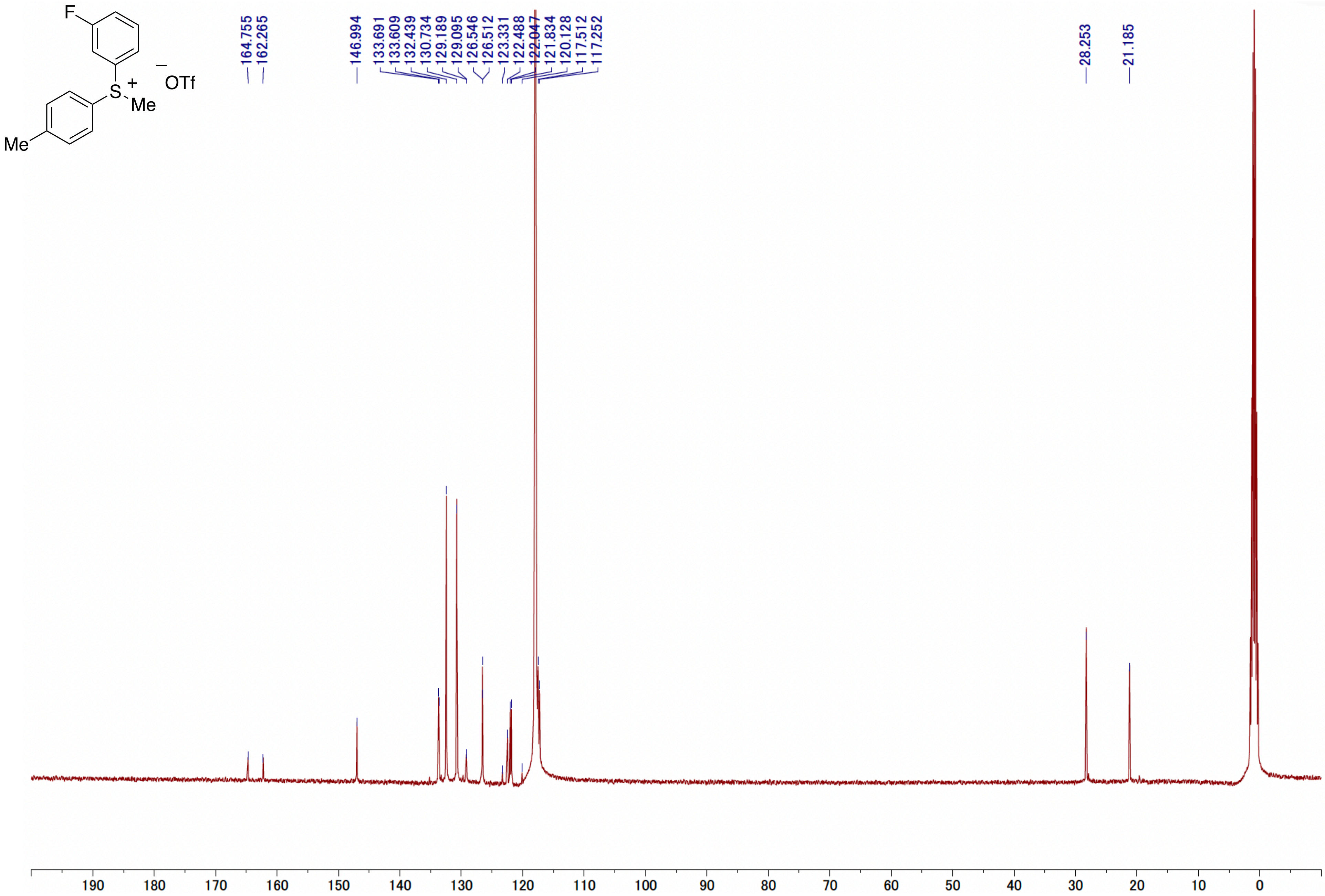

^{19}F NMR (376 MHz, $CDCl_3$) ; **3b**

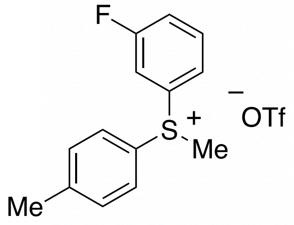

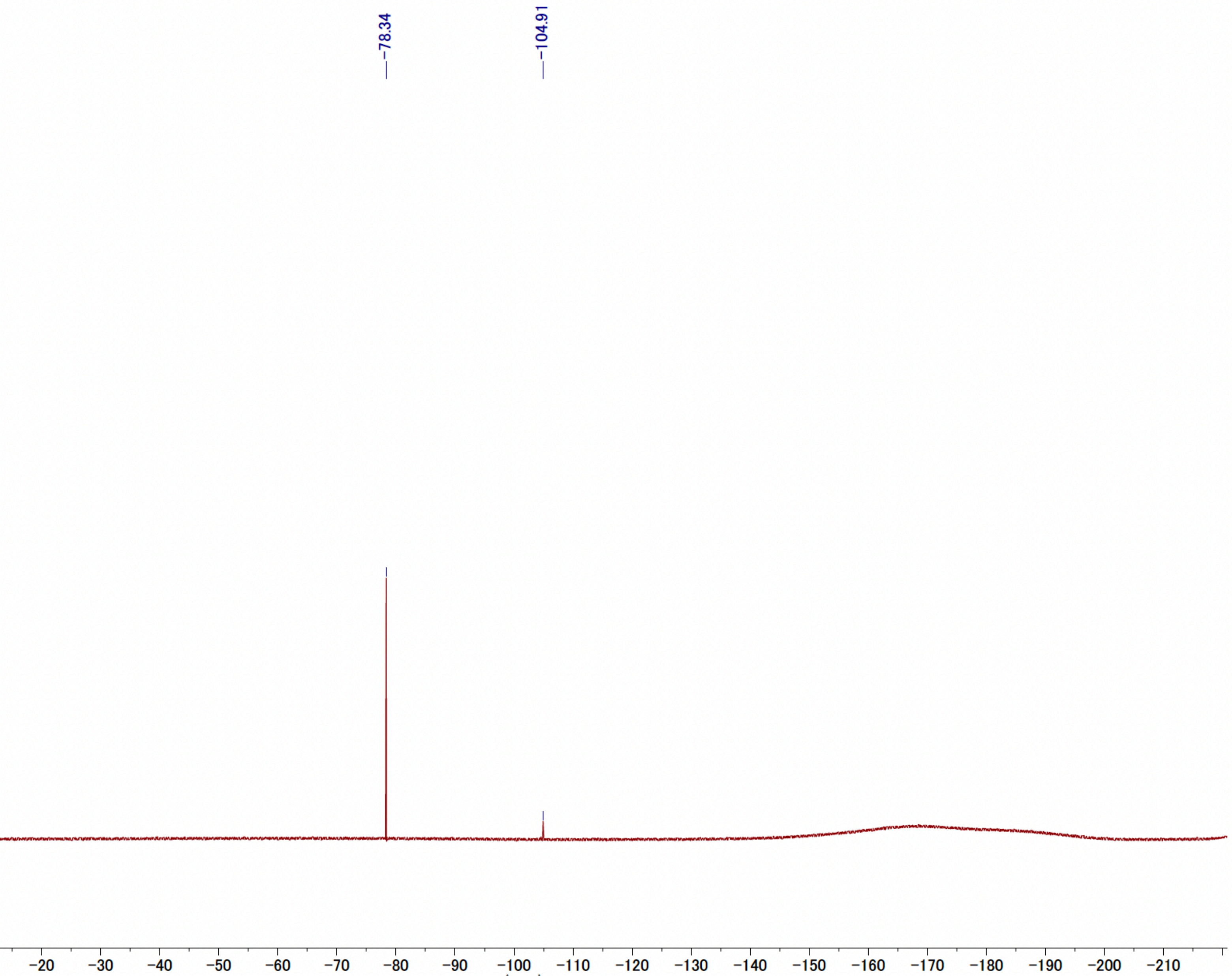

^{1}H NMR (400 MHz, $CDCl_3$) ; **3c**

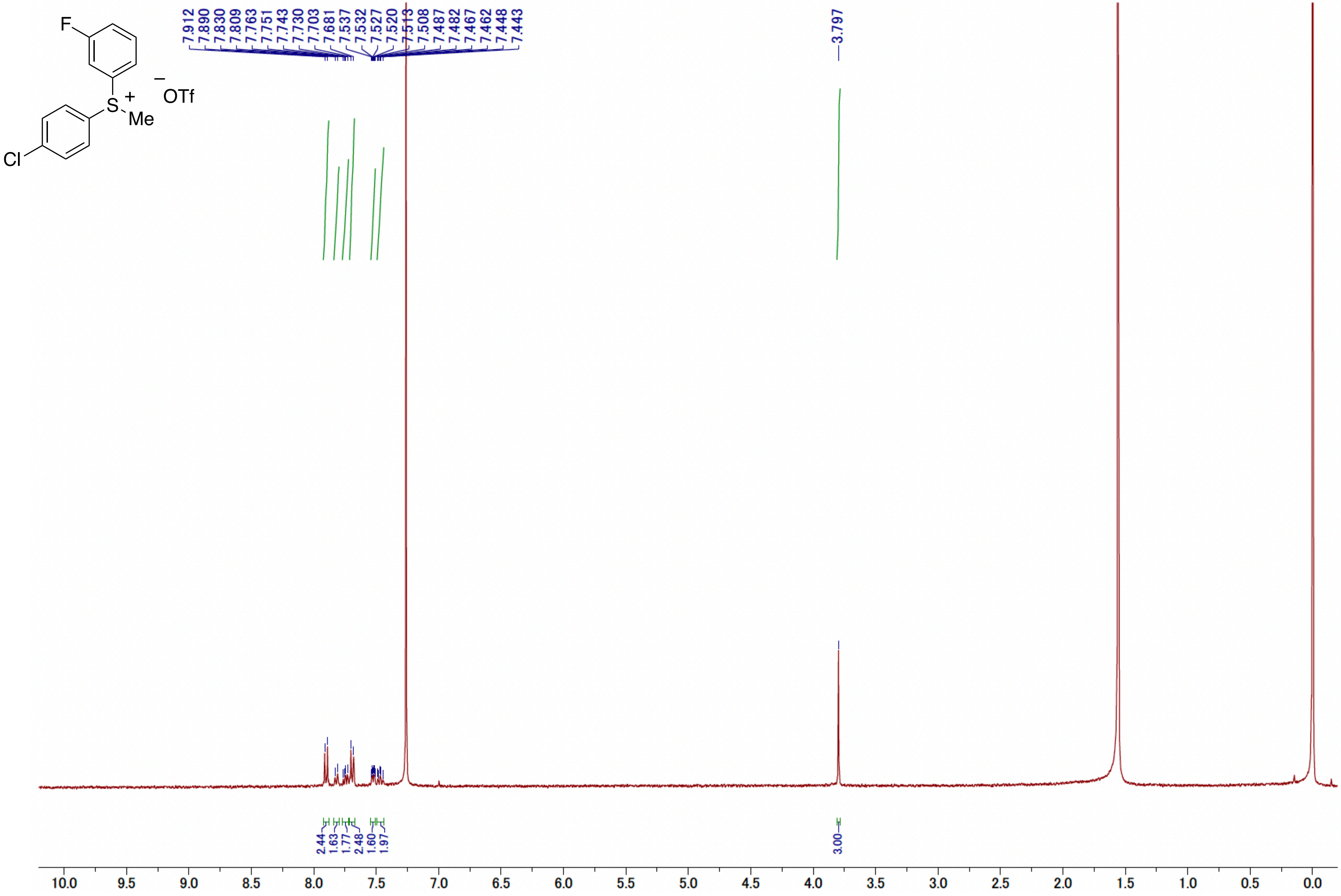

^{13}C NMR (100 MHz, CD_3CN) ; **3c**

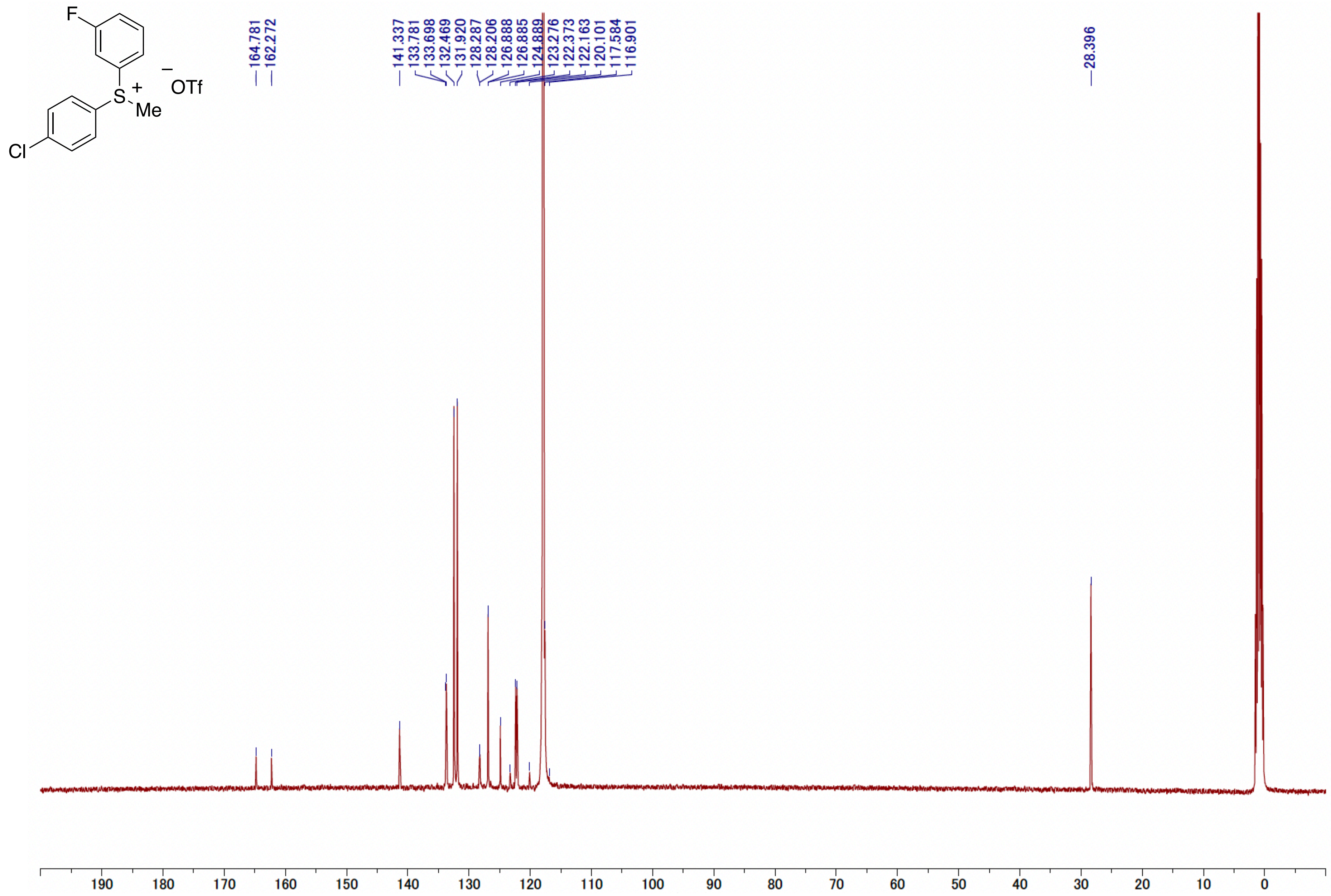

^{19}F NMR (376 MHz, CD_3CN) ; **3c**

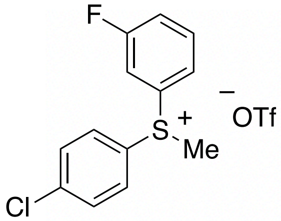

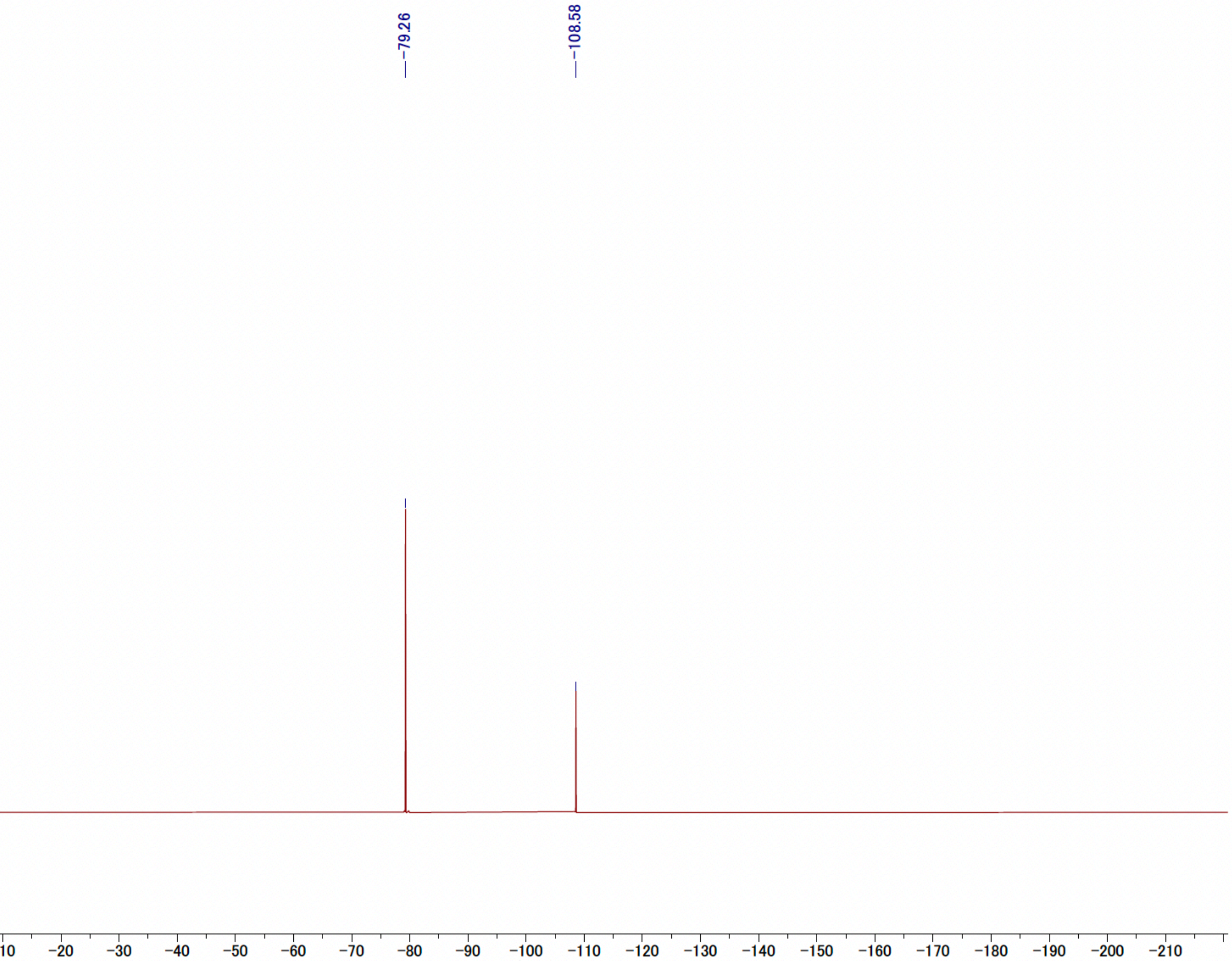

^{1}H NMR (400 MHz, CD_3CN) ; **3d**

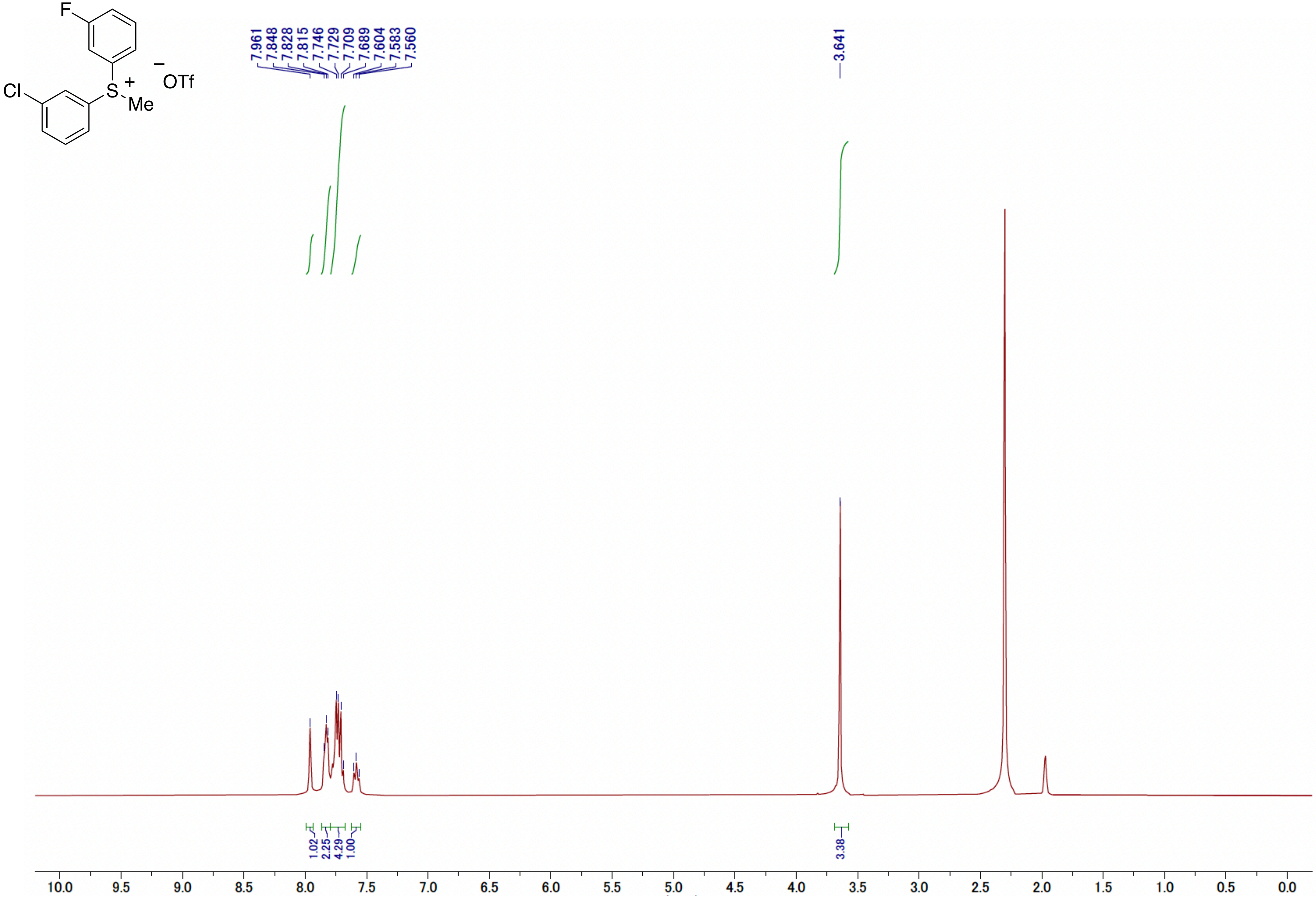

^{13}C NMR (100 MHz, CD_3CN) ; **3d**

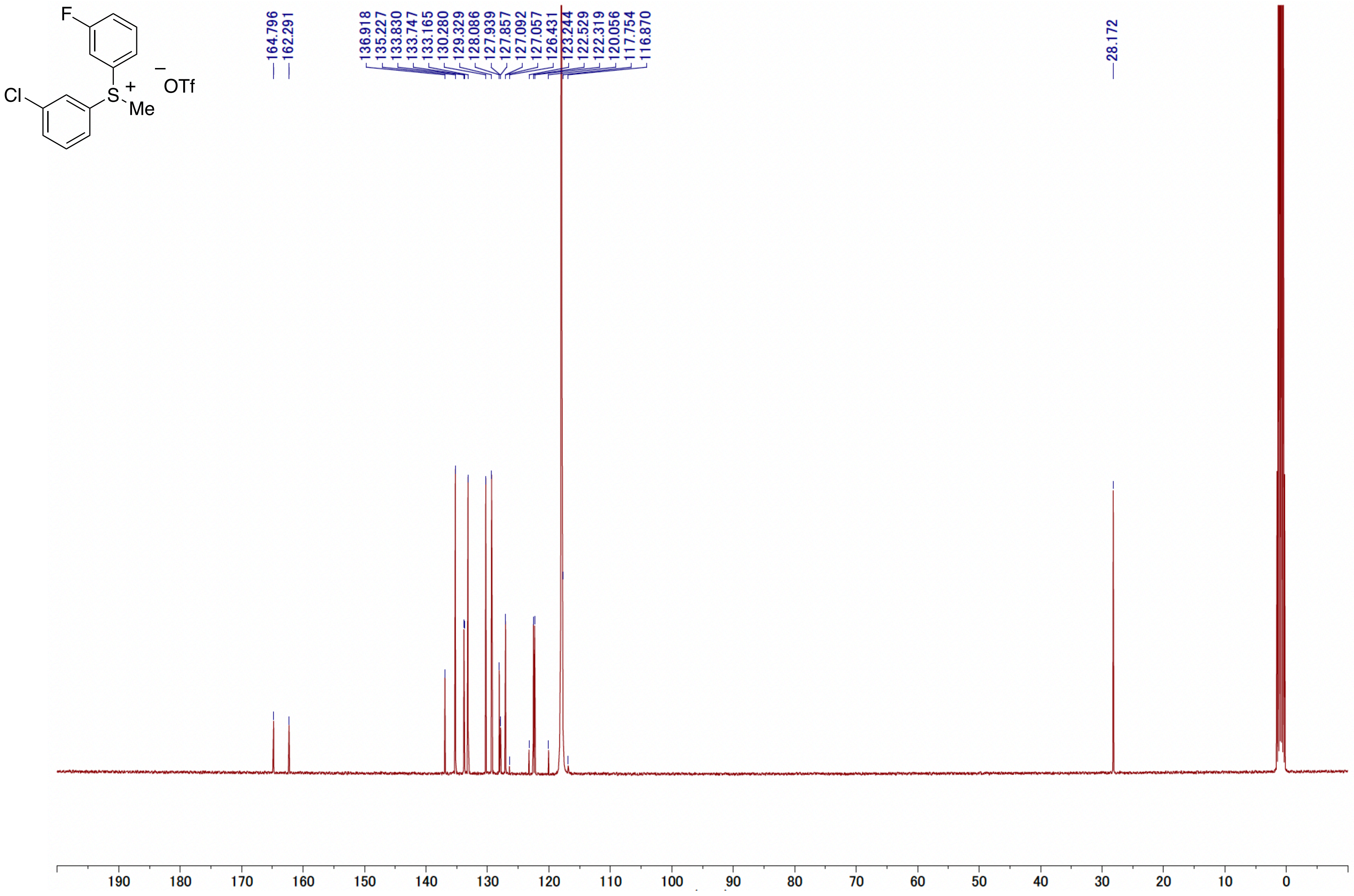

^{19}F NMR (376 MHz, CD_3CN) ; **3d**

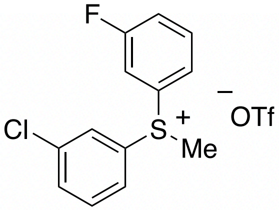

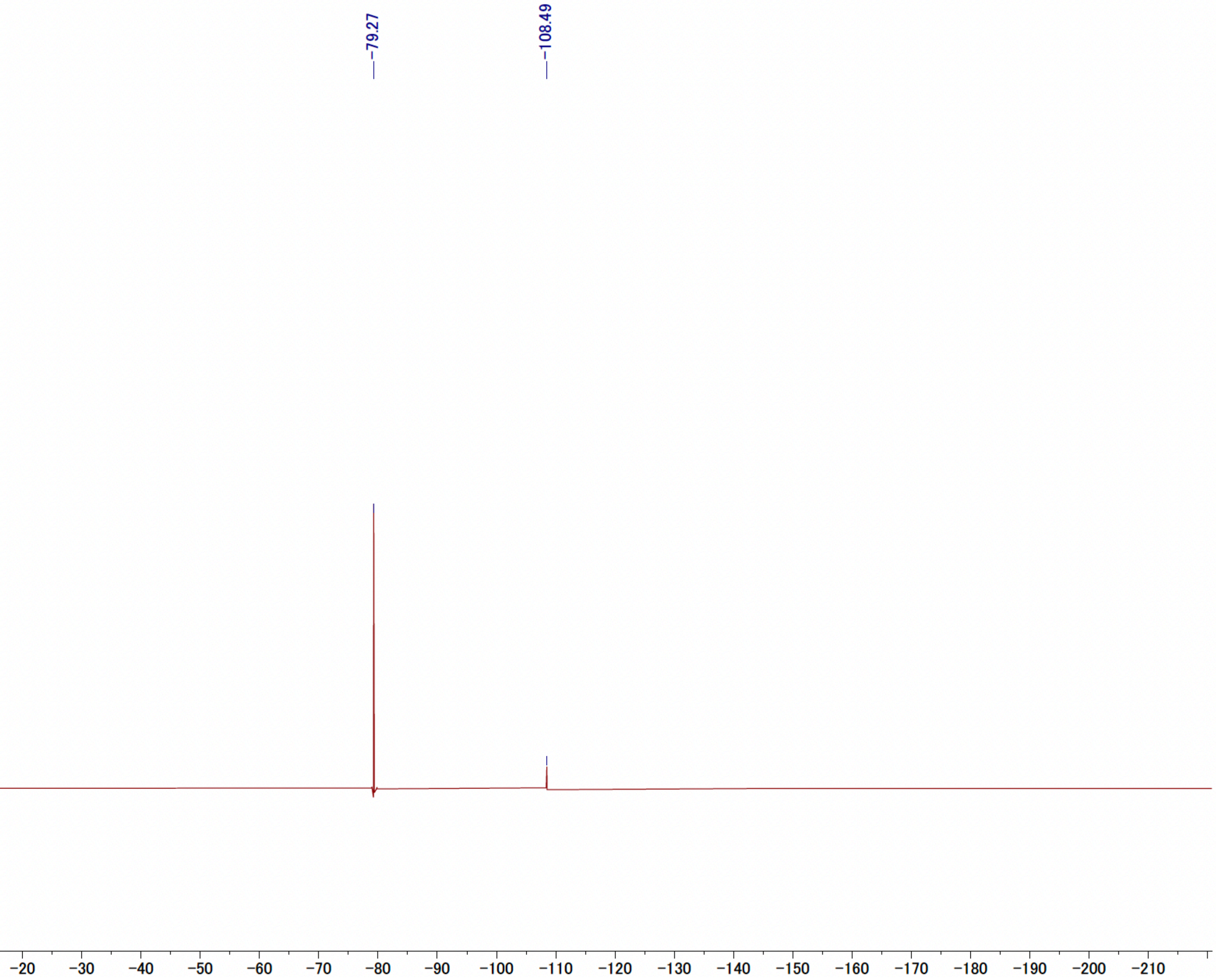

^{1}H NMR (400 MHz, $CDCl_3$) ; **3e**

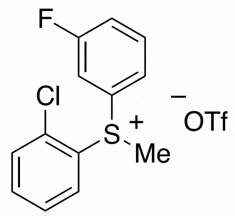

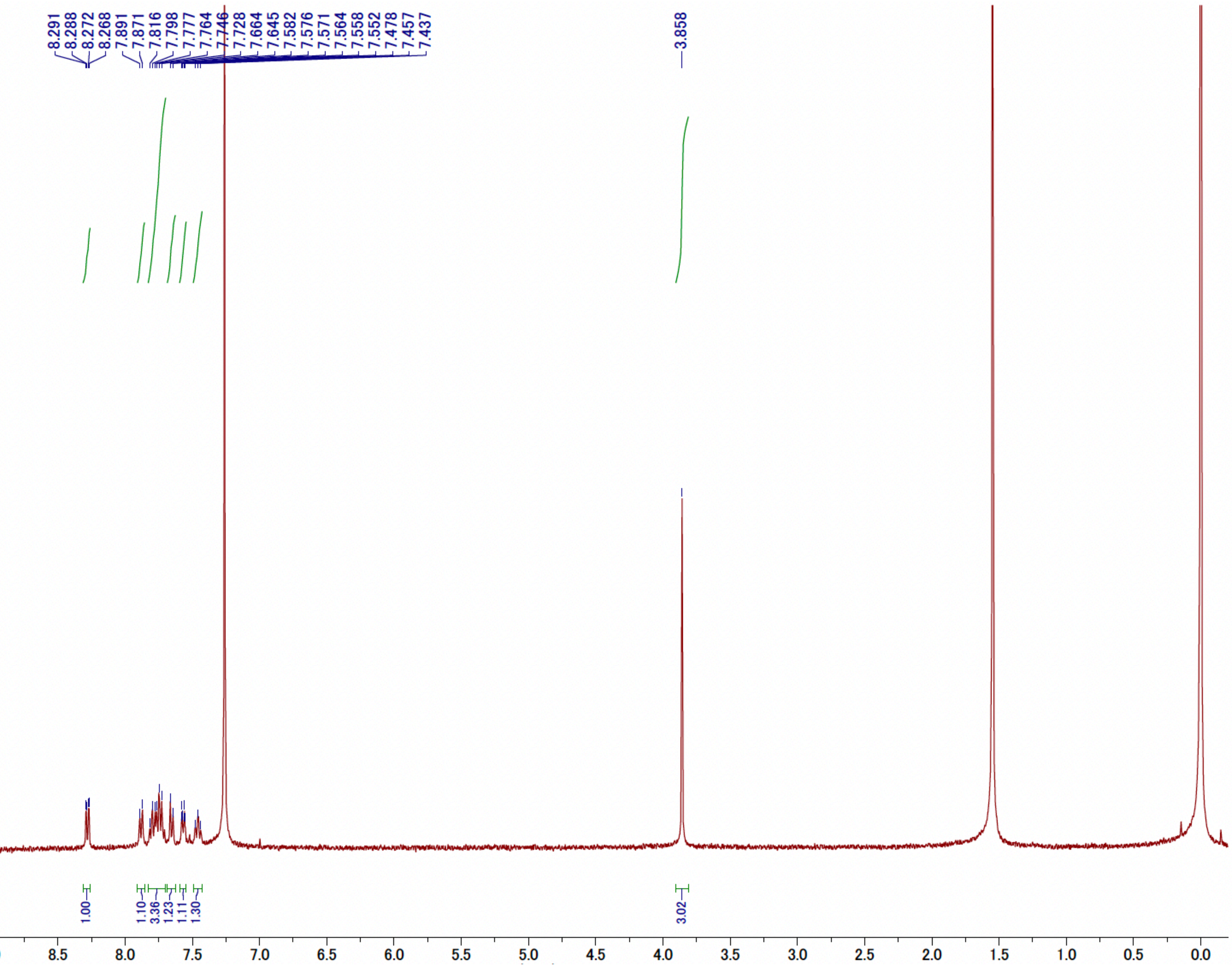

^{13}C NMR (100 MHz, CD_3CN) ; **3e**

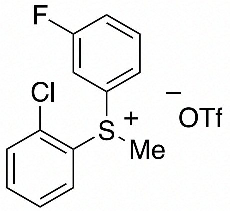

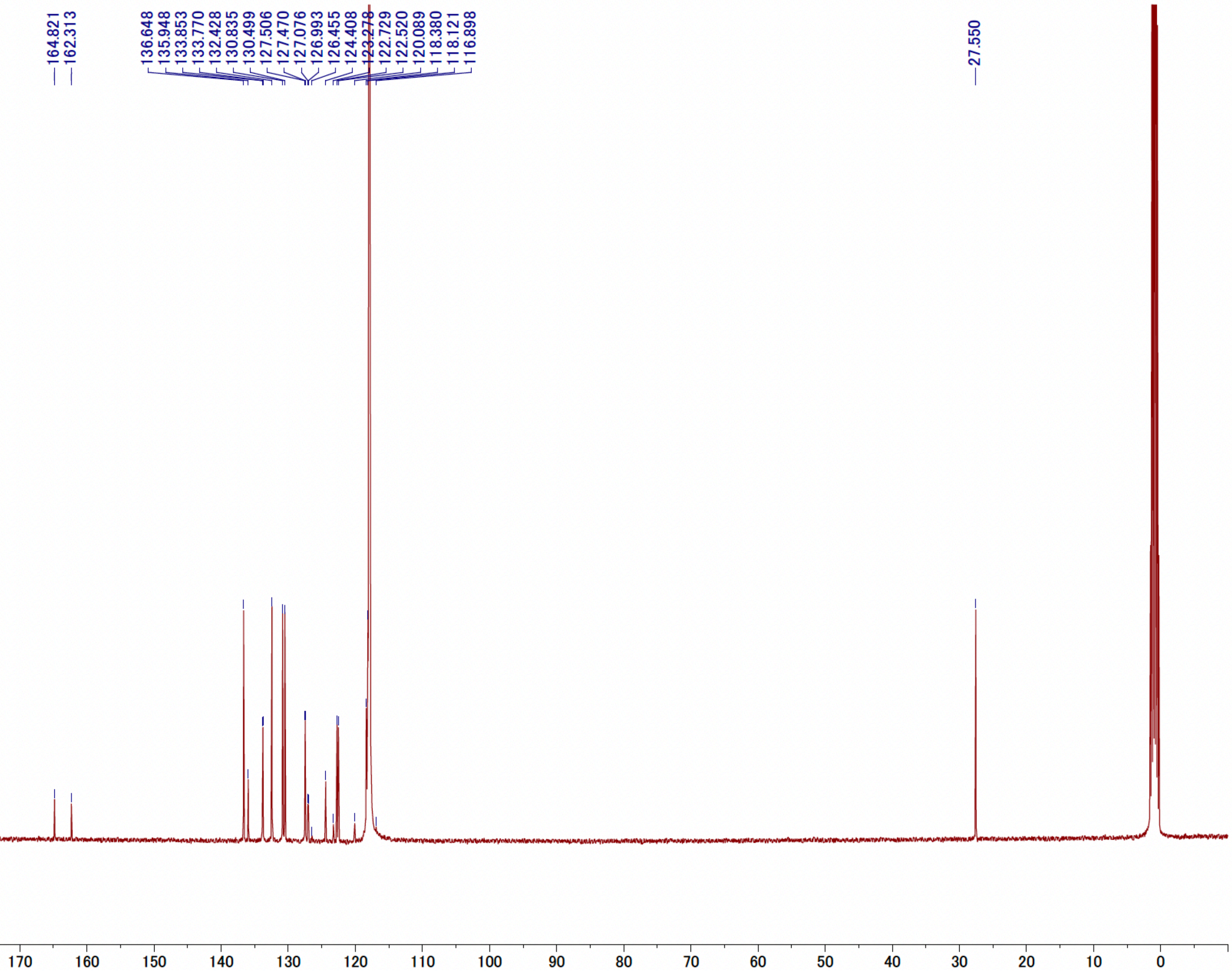

^{19}F NMR (376 MHz, CD_3CN) ; **3e**

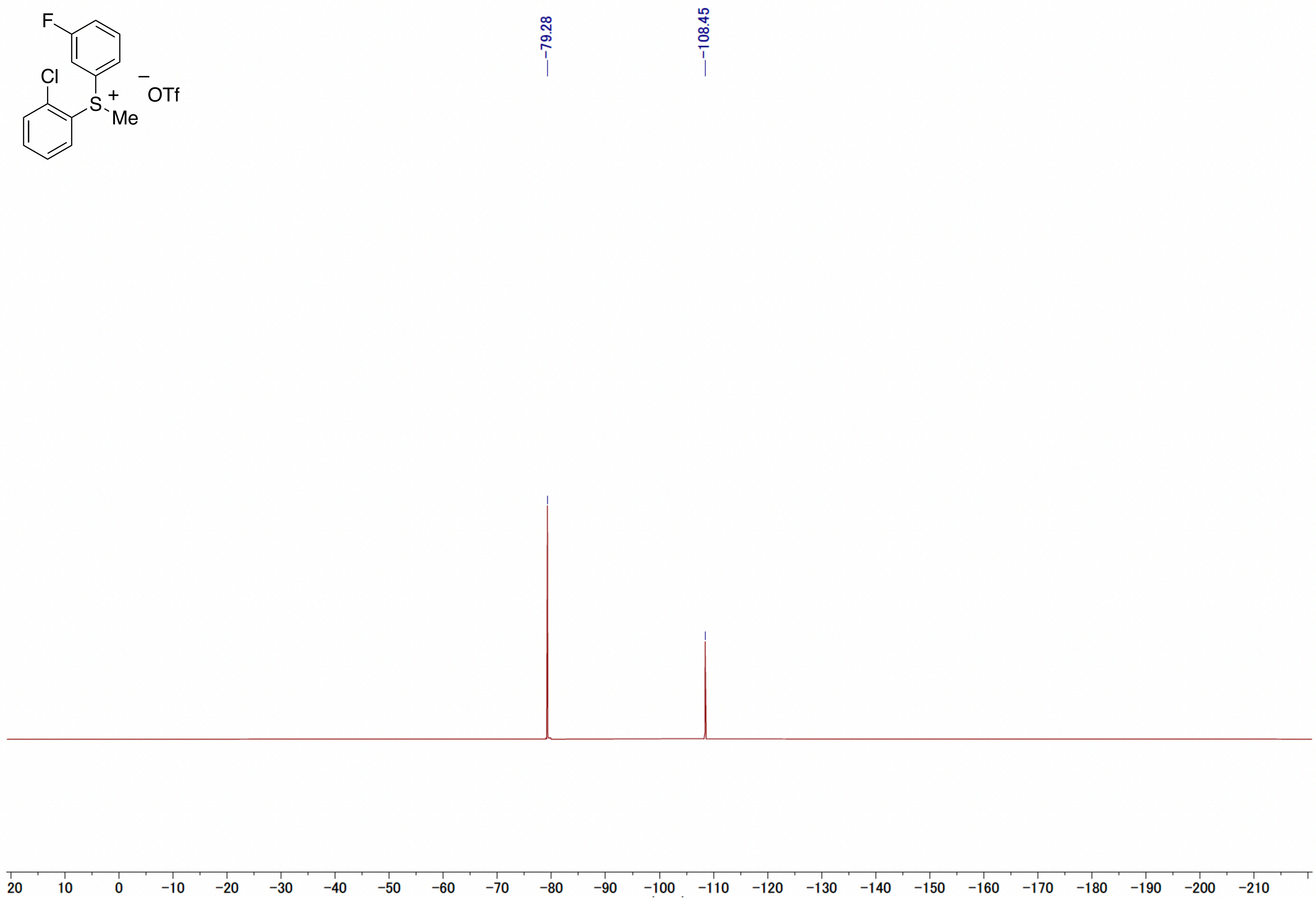

^{1}H NMR (400 MHz, $CDCl_3$) ; **3f**

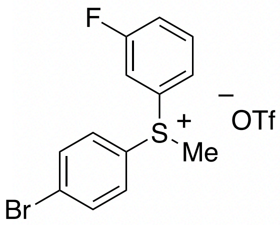

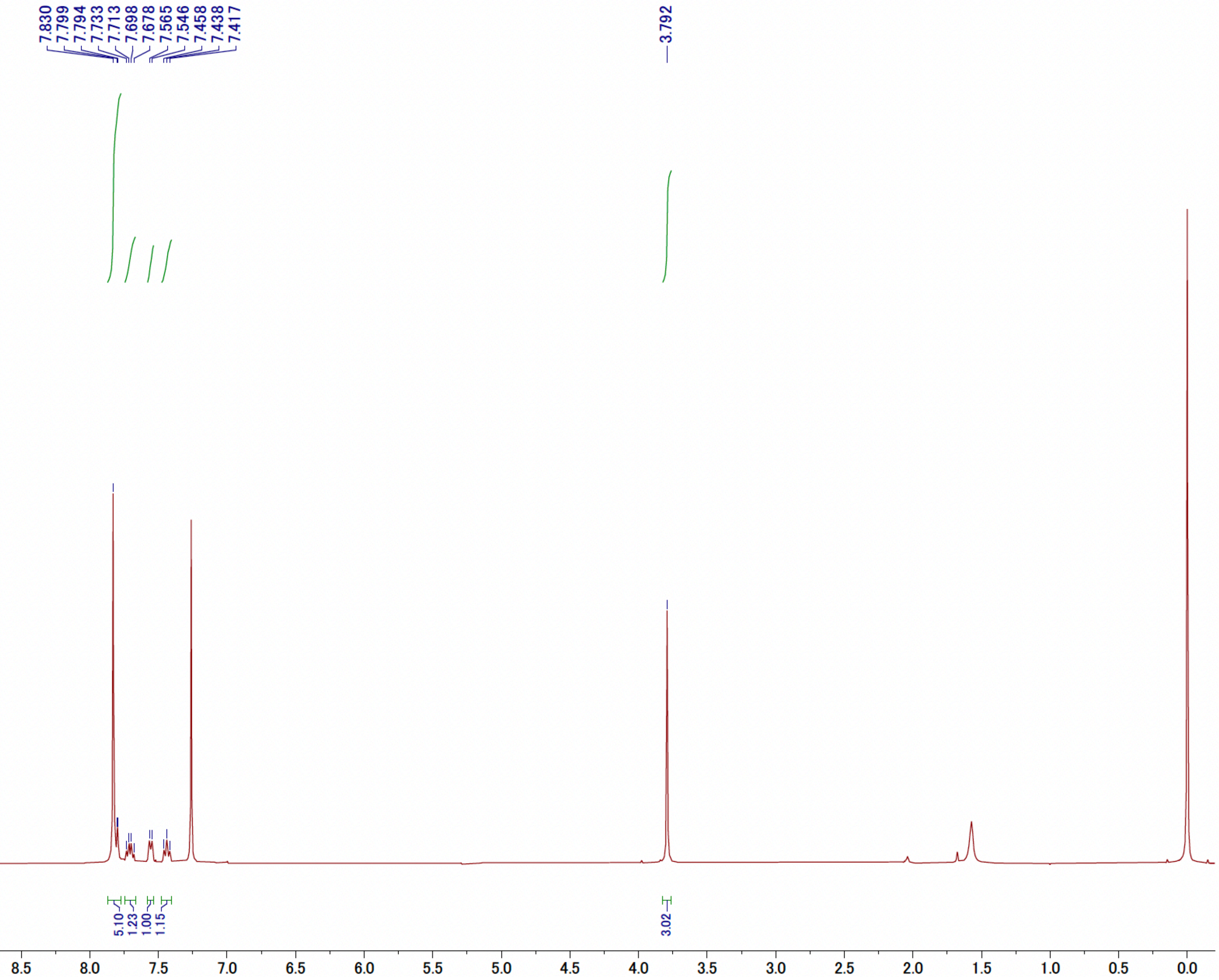

^{13}C NMR (100 MHz, CD_3CN) ; **3f**

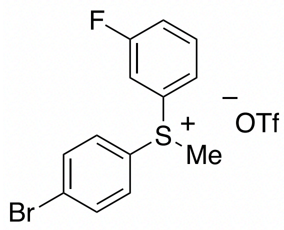

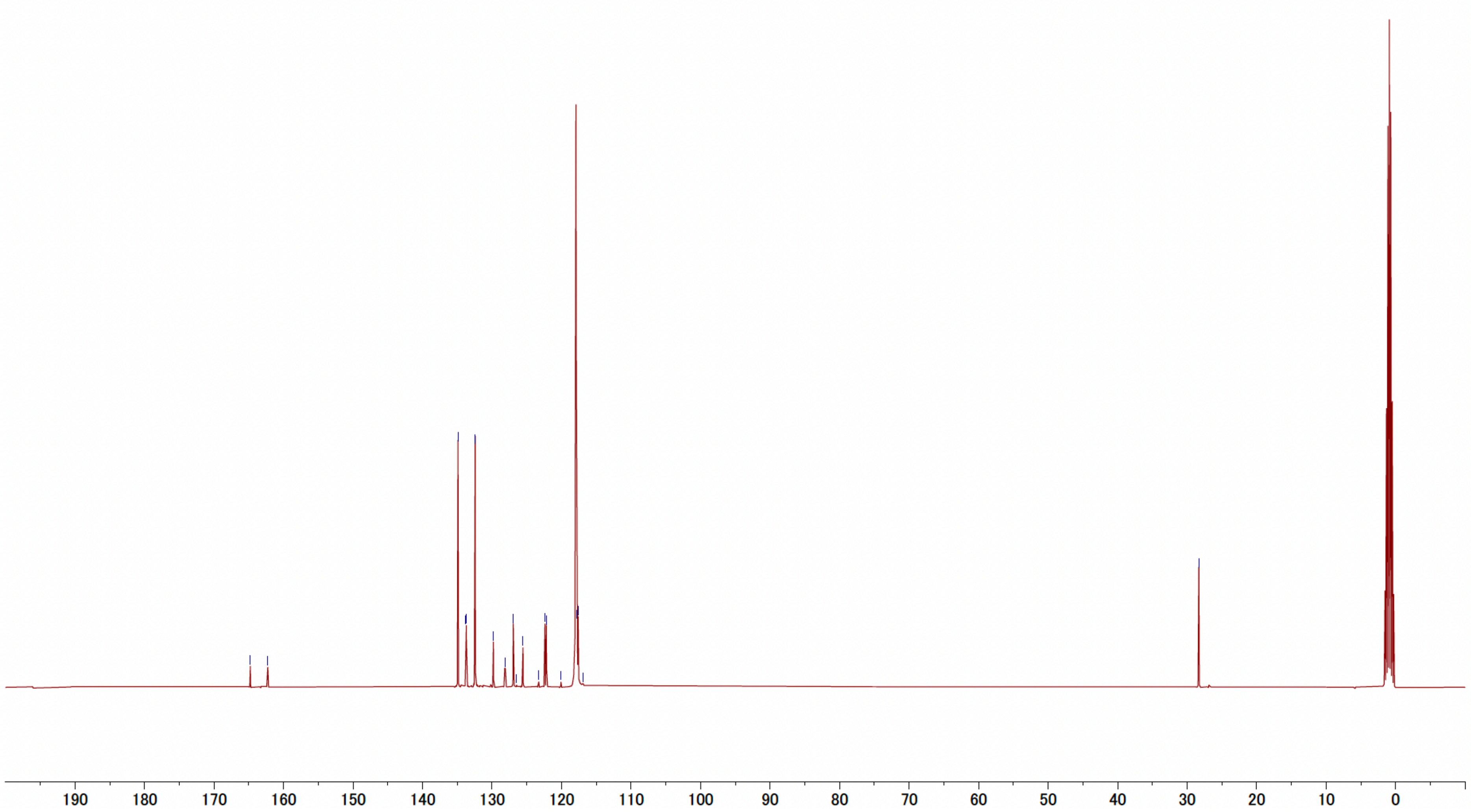

^{19}F NMR (376 MHz, CD_3CN) ; **3f**

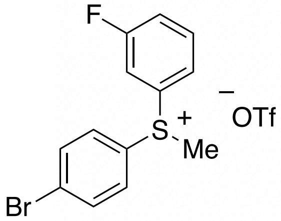

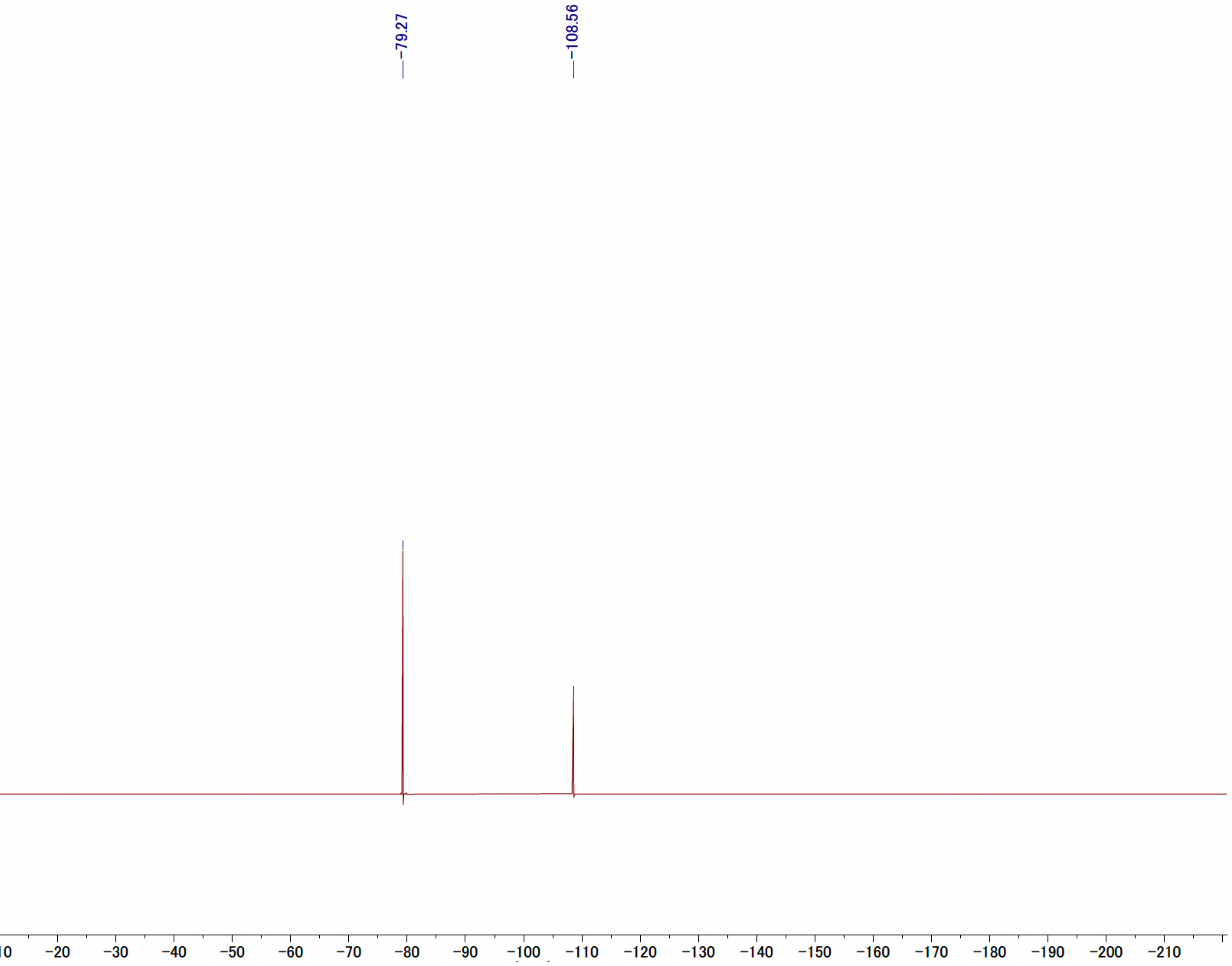

^{1}H NMR (400 MHz, $CDCl_3$) ; **3g**

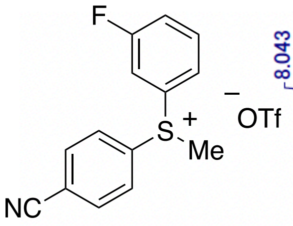

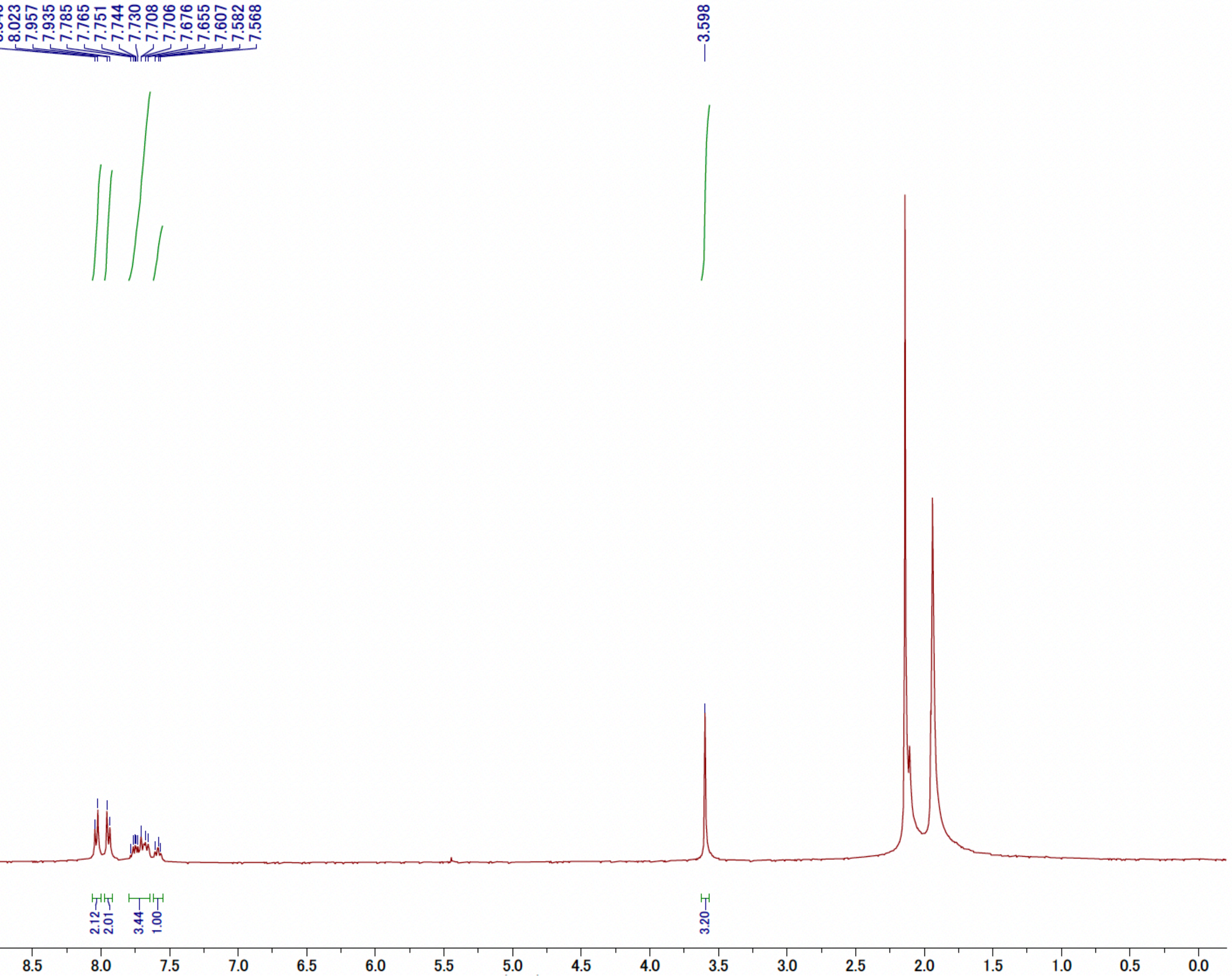

^{13}C NMR (100 MHz, CD_3CN) ; **3g**

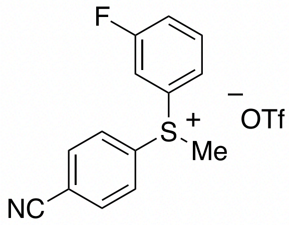

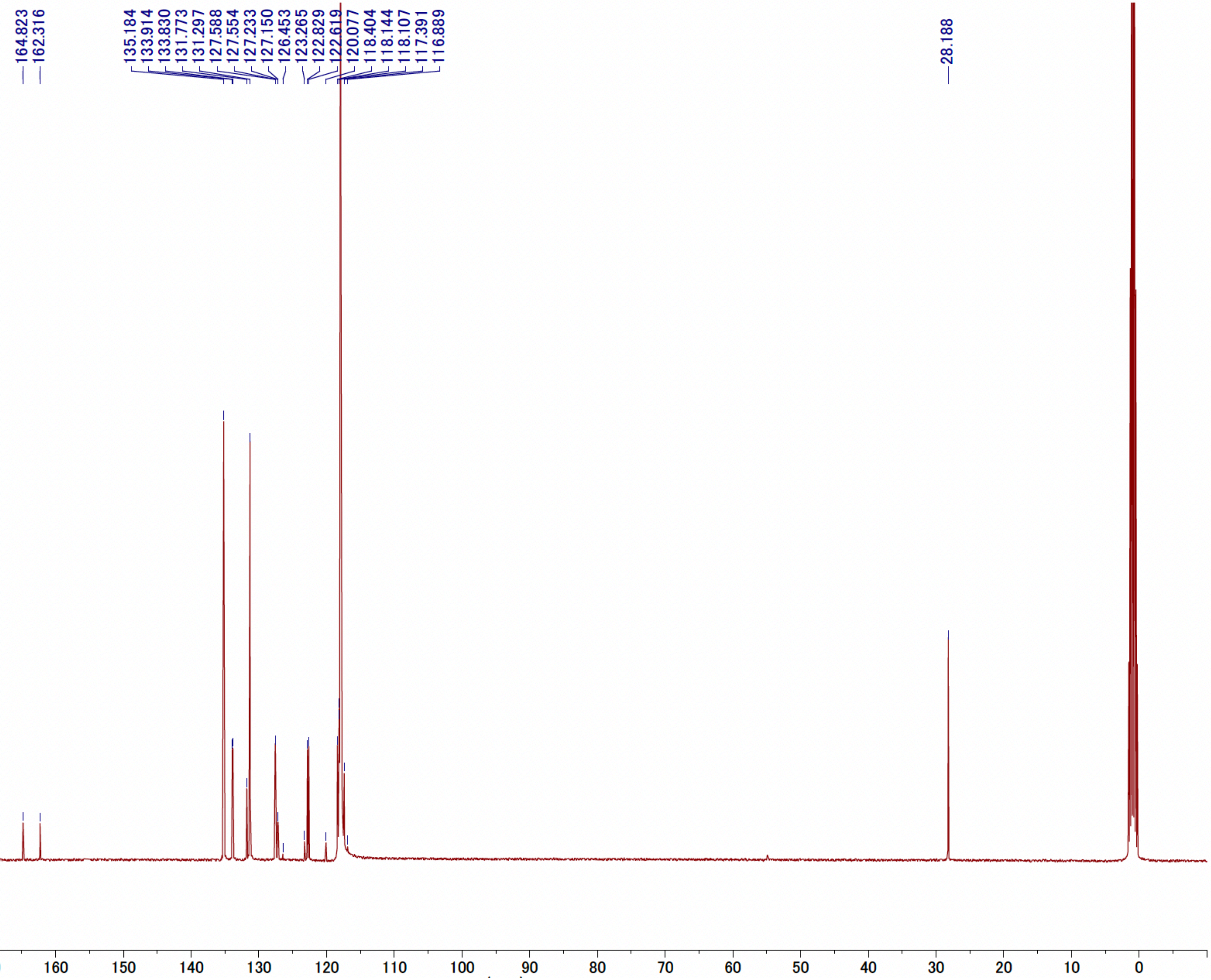

^{1}H NMR (400 MHz, CD_3CN) ; **3h**

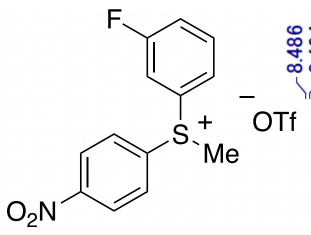

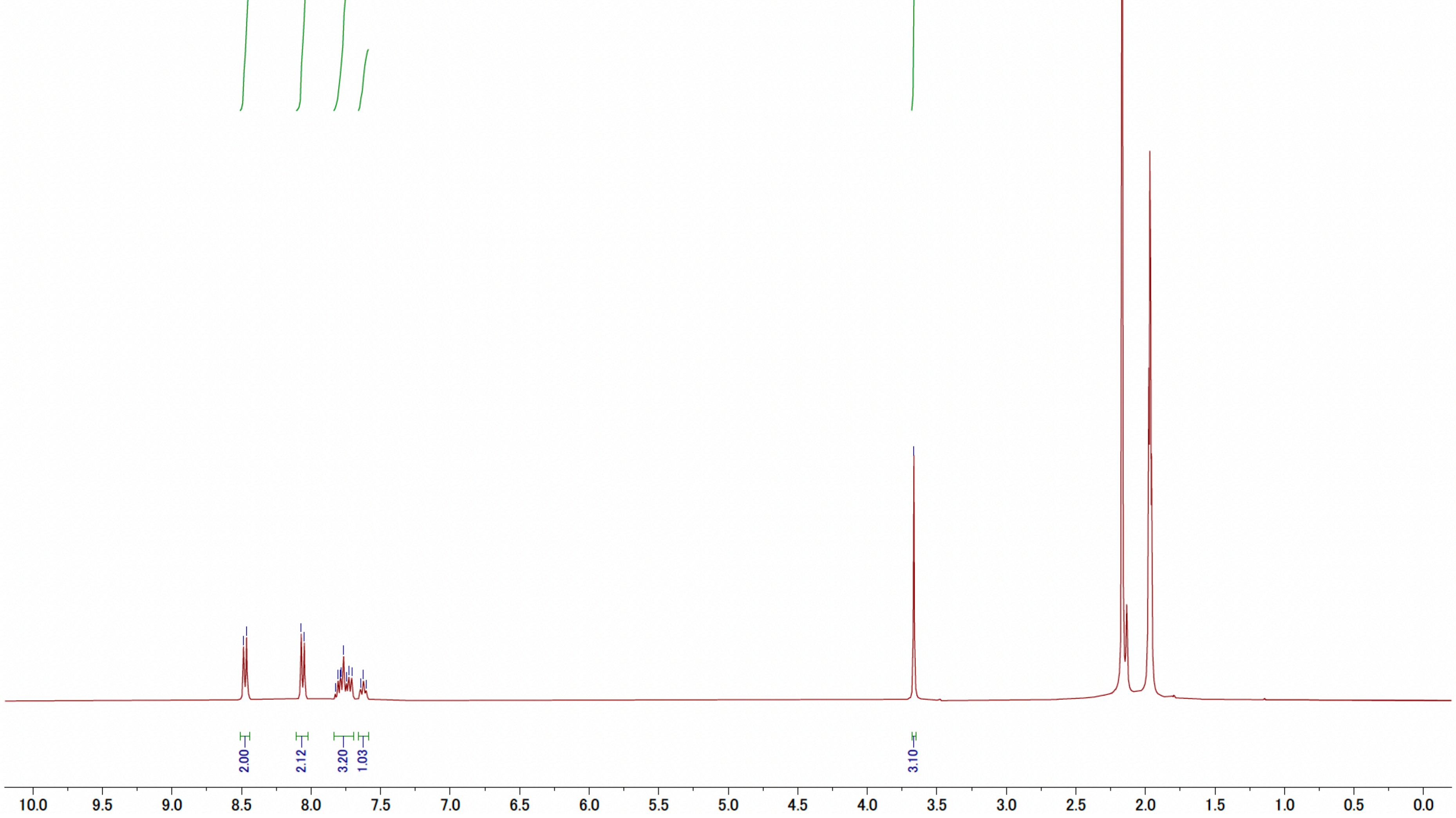

^{13}C NMR (100 MHz, CD_3CN) ; **3h**

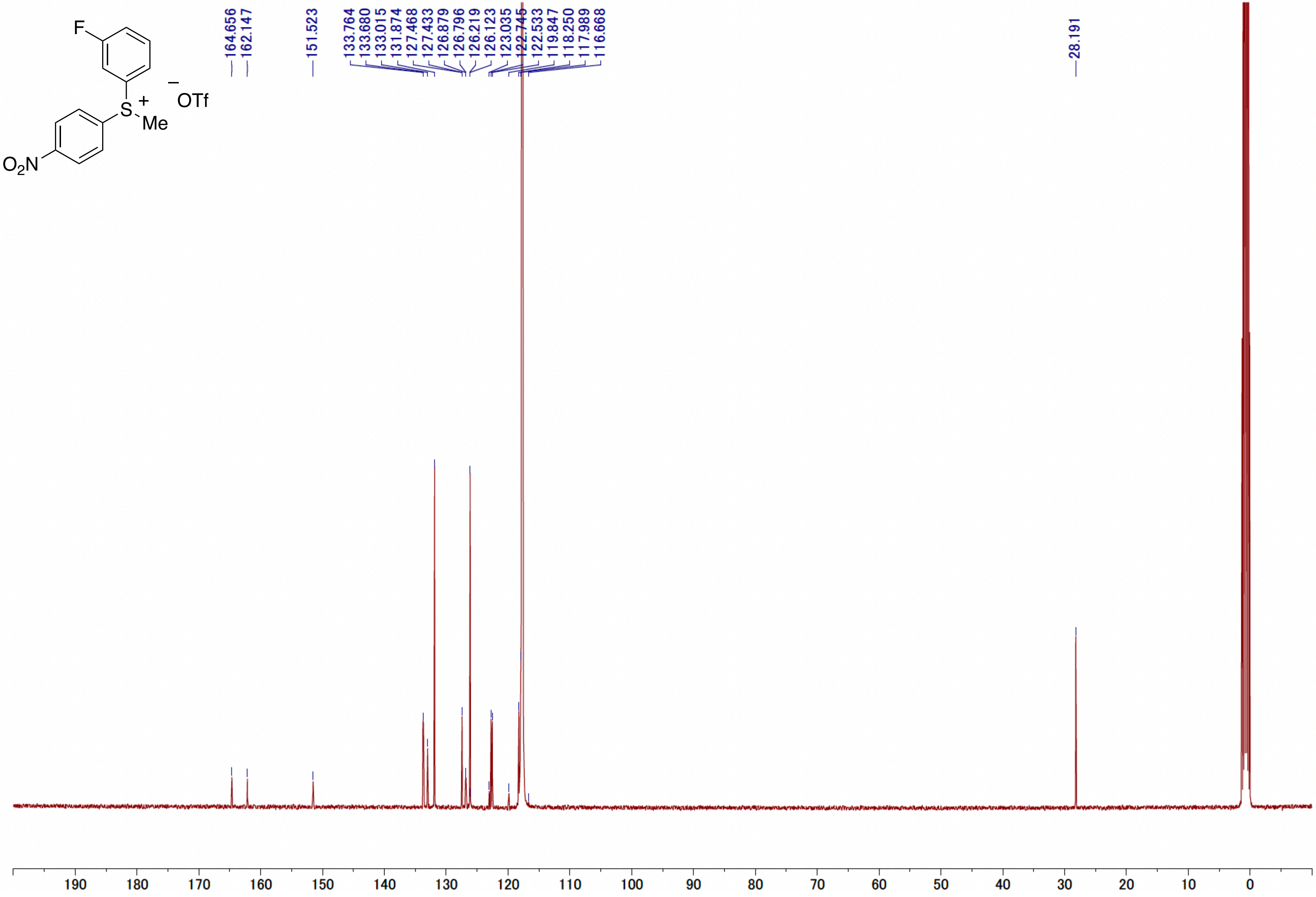

^{1}H NMR (400 MHz, $CDCl_3$) ; **3i**

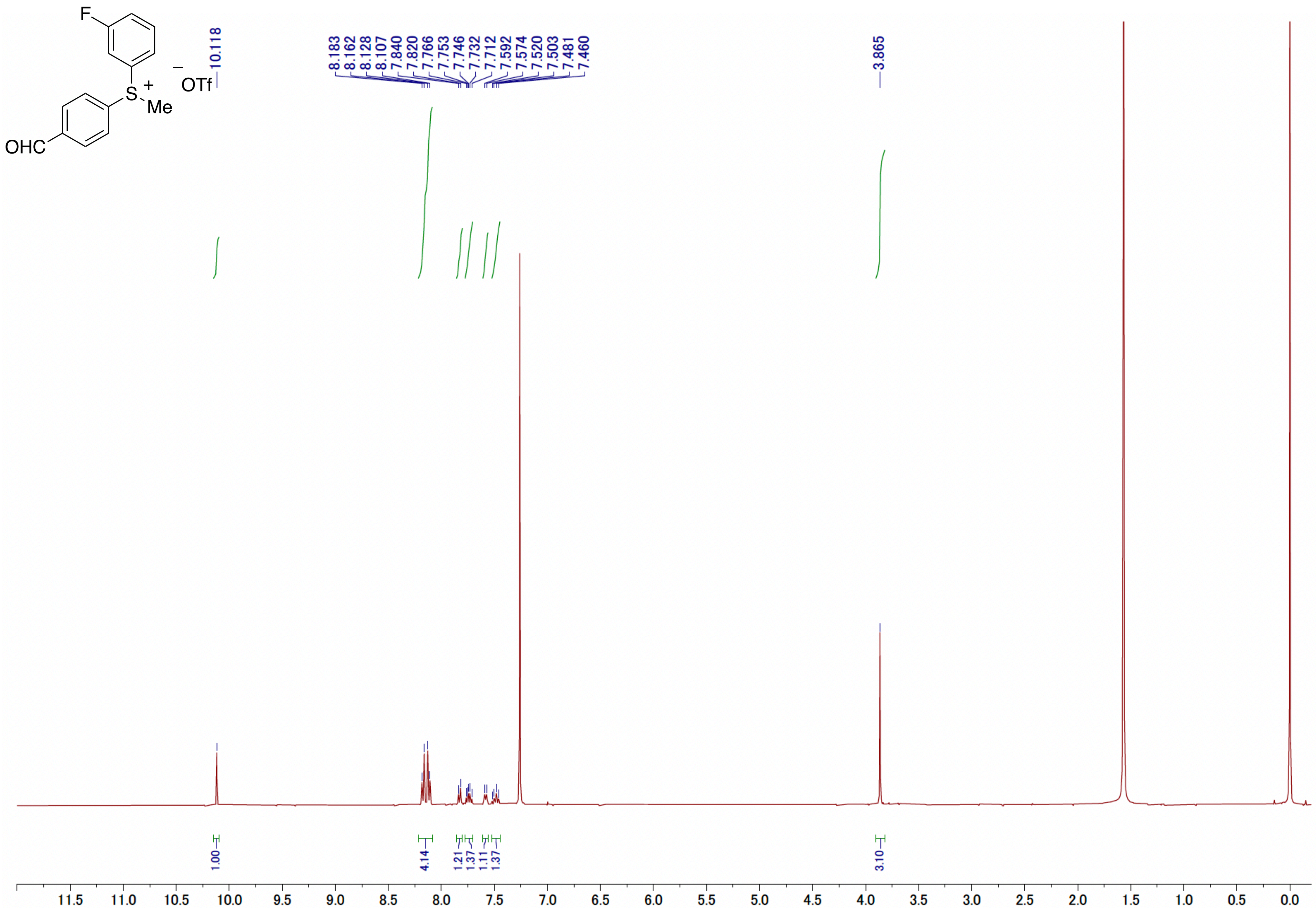

^{13}C NMR (100 MHz, CD_3CN) ; **3i**

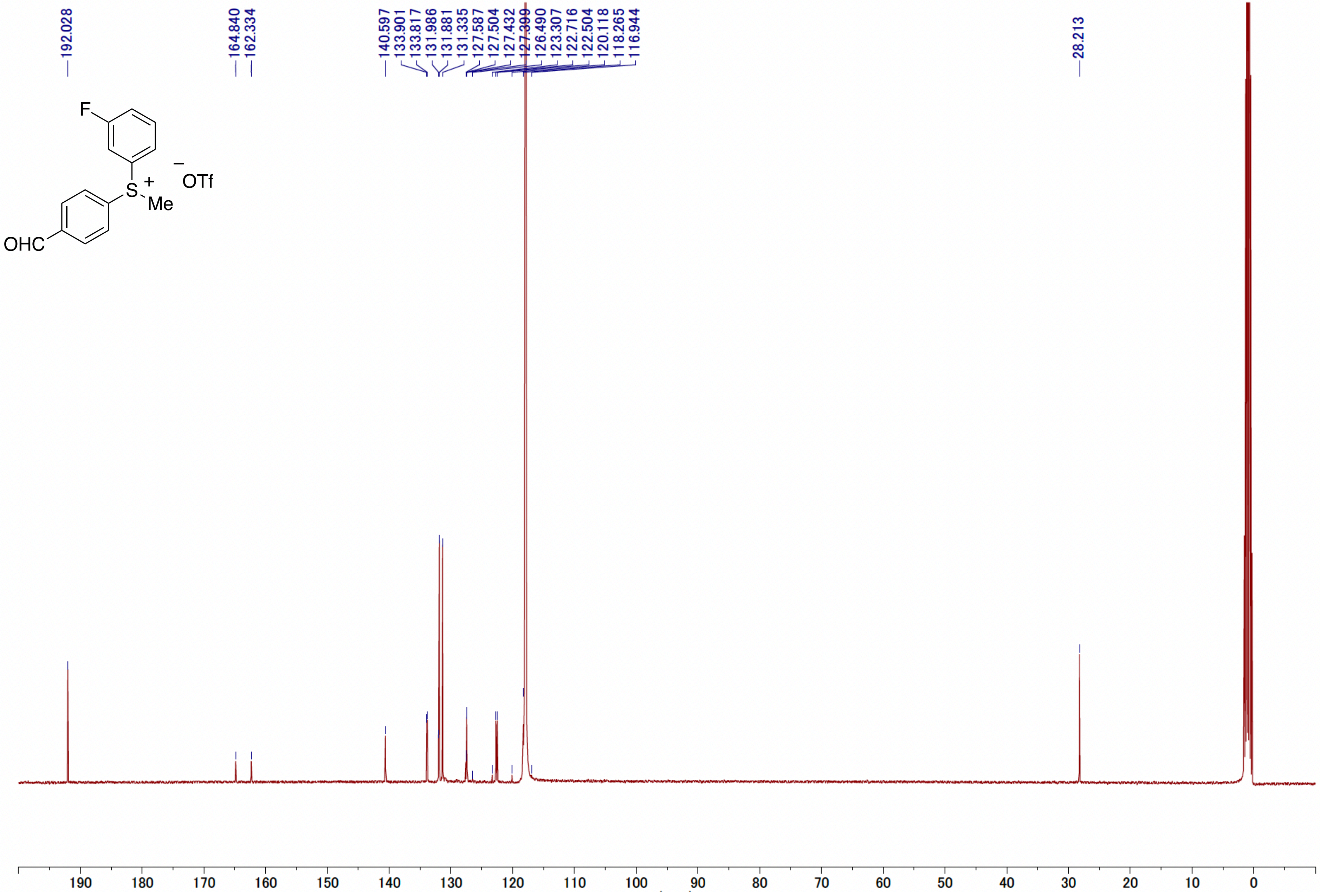

^{1}H NMR (400 MHz, $CDCl_3$) ; **3j**

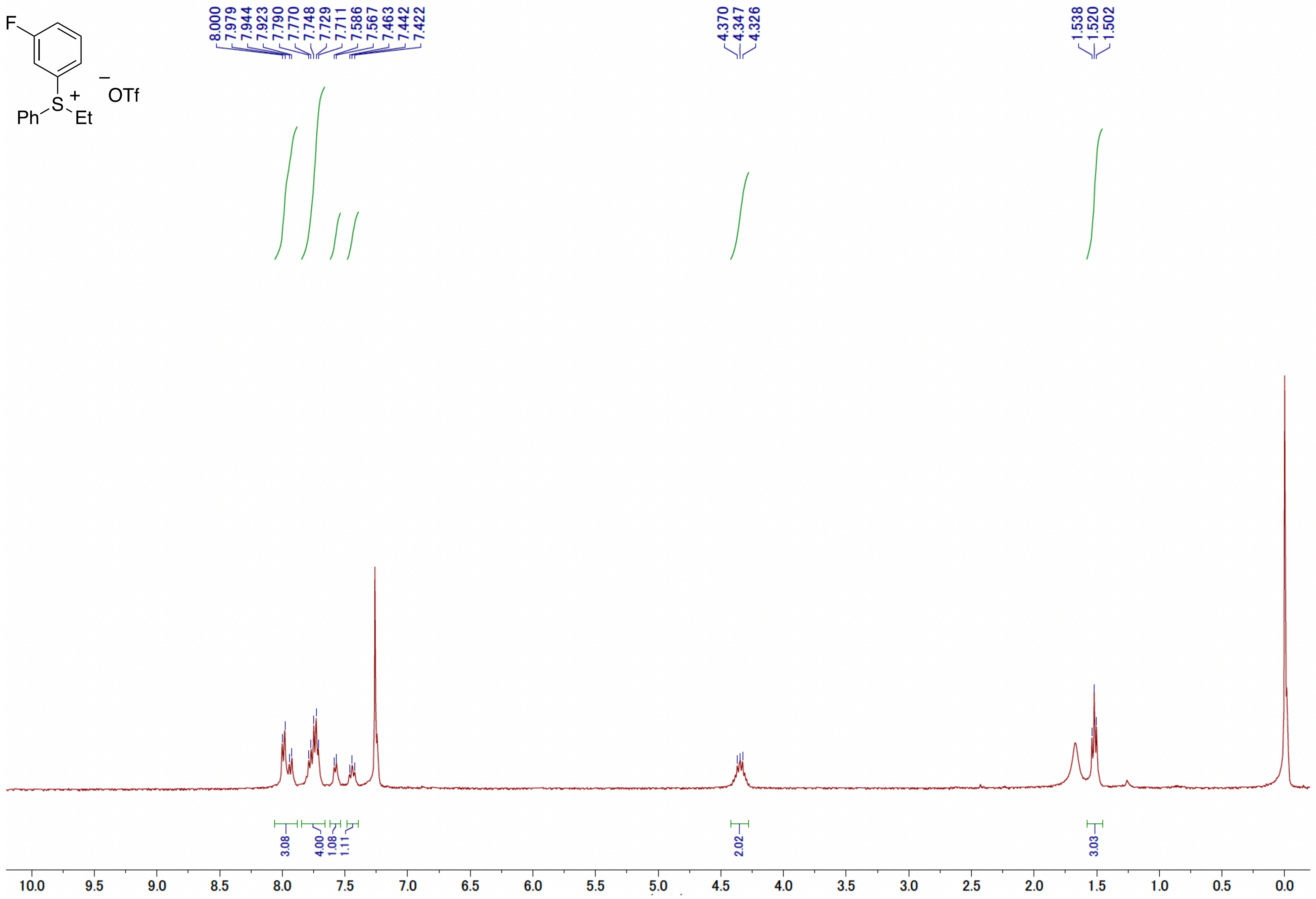

^{13}C NMR (100 MHz, CD_3CN) ; **3j**

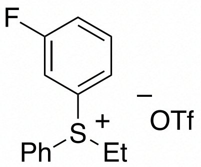

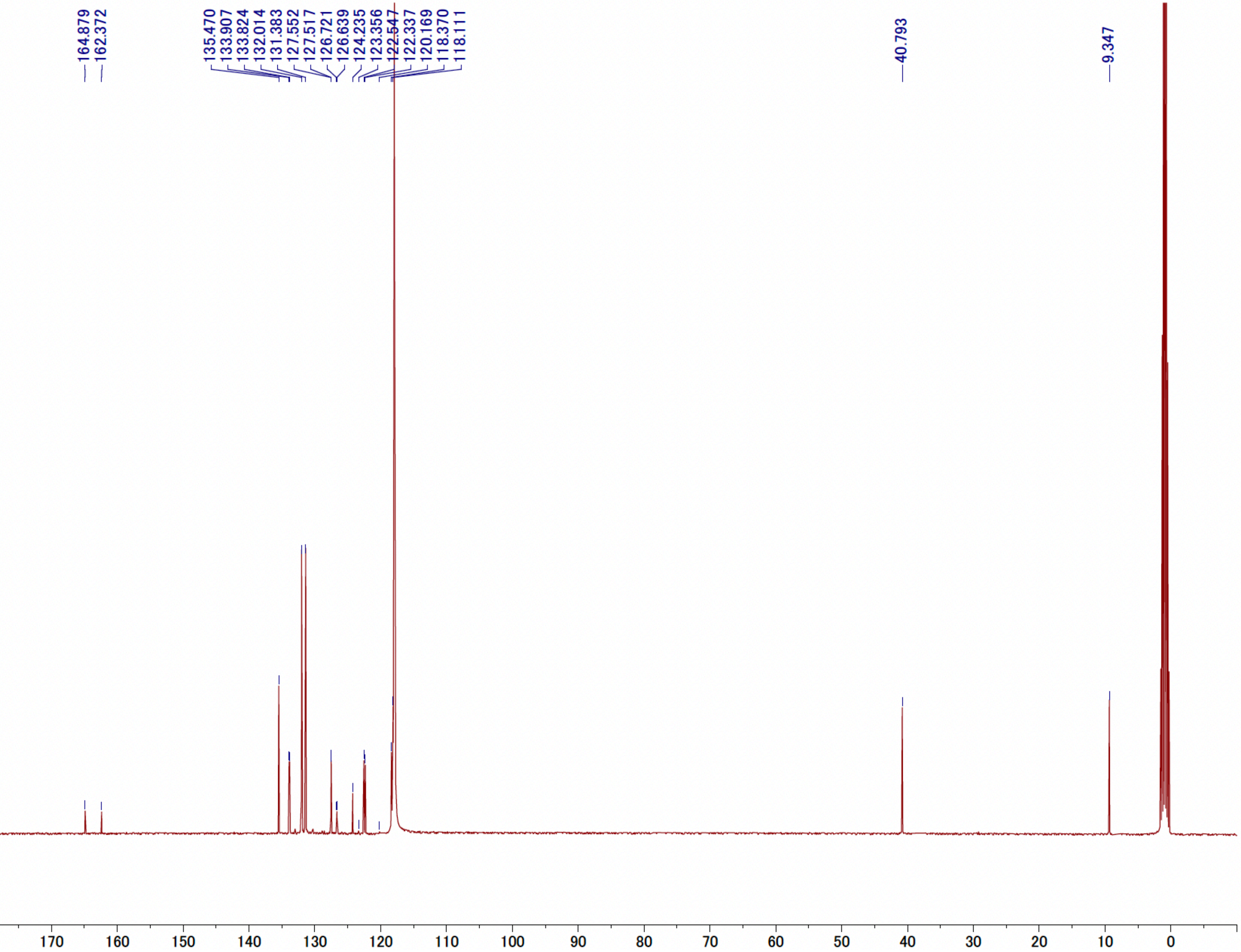

^{19}F NMR (376 MHz, CD_3CN) ; **3j**

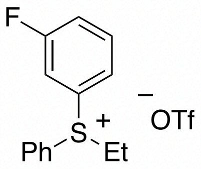

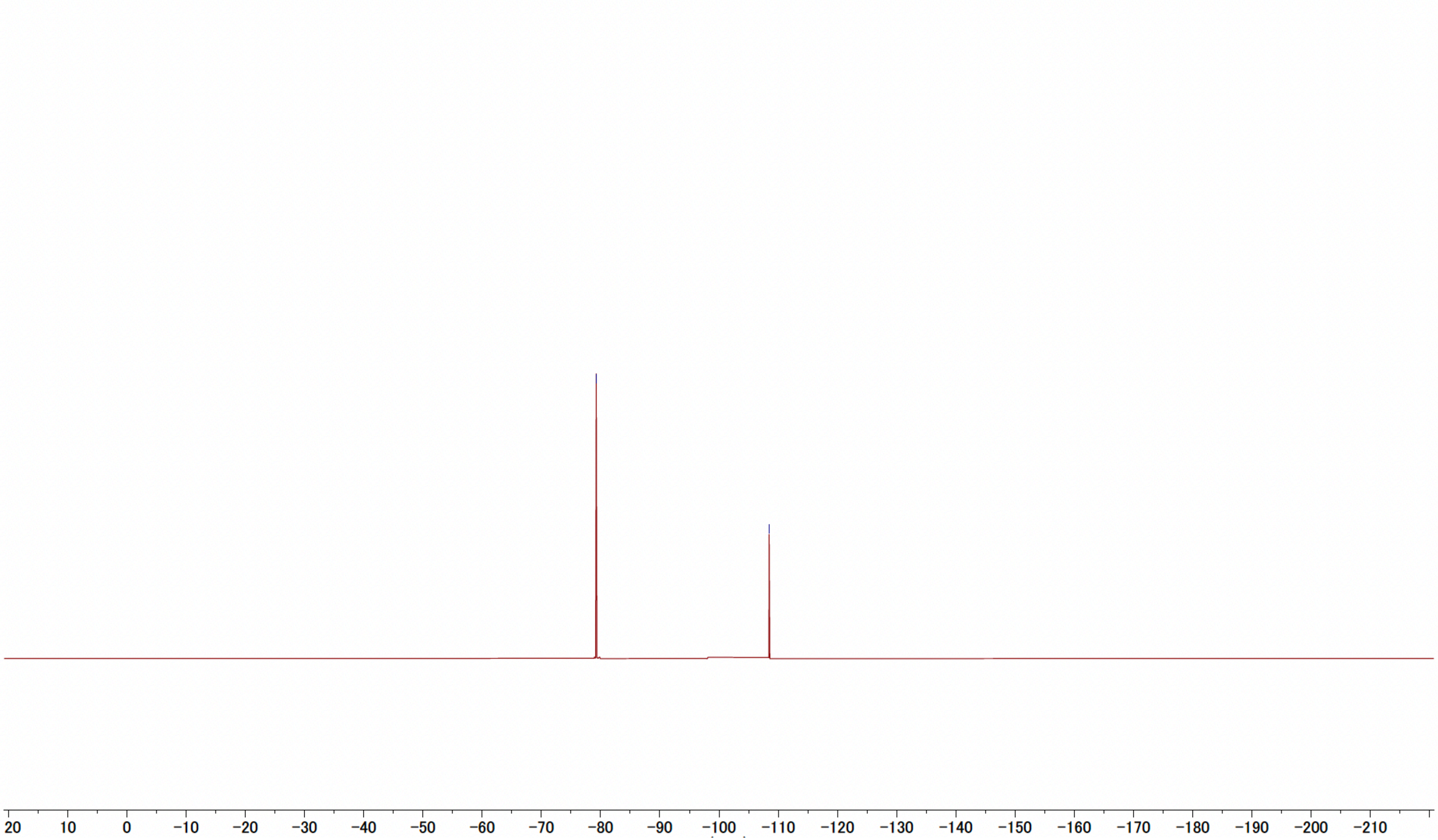

^{1}H NMR (400 MHz, CD_3CN) ; **3k**

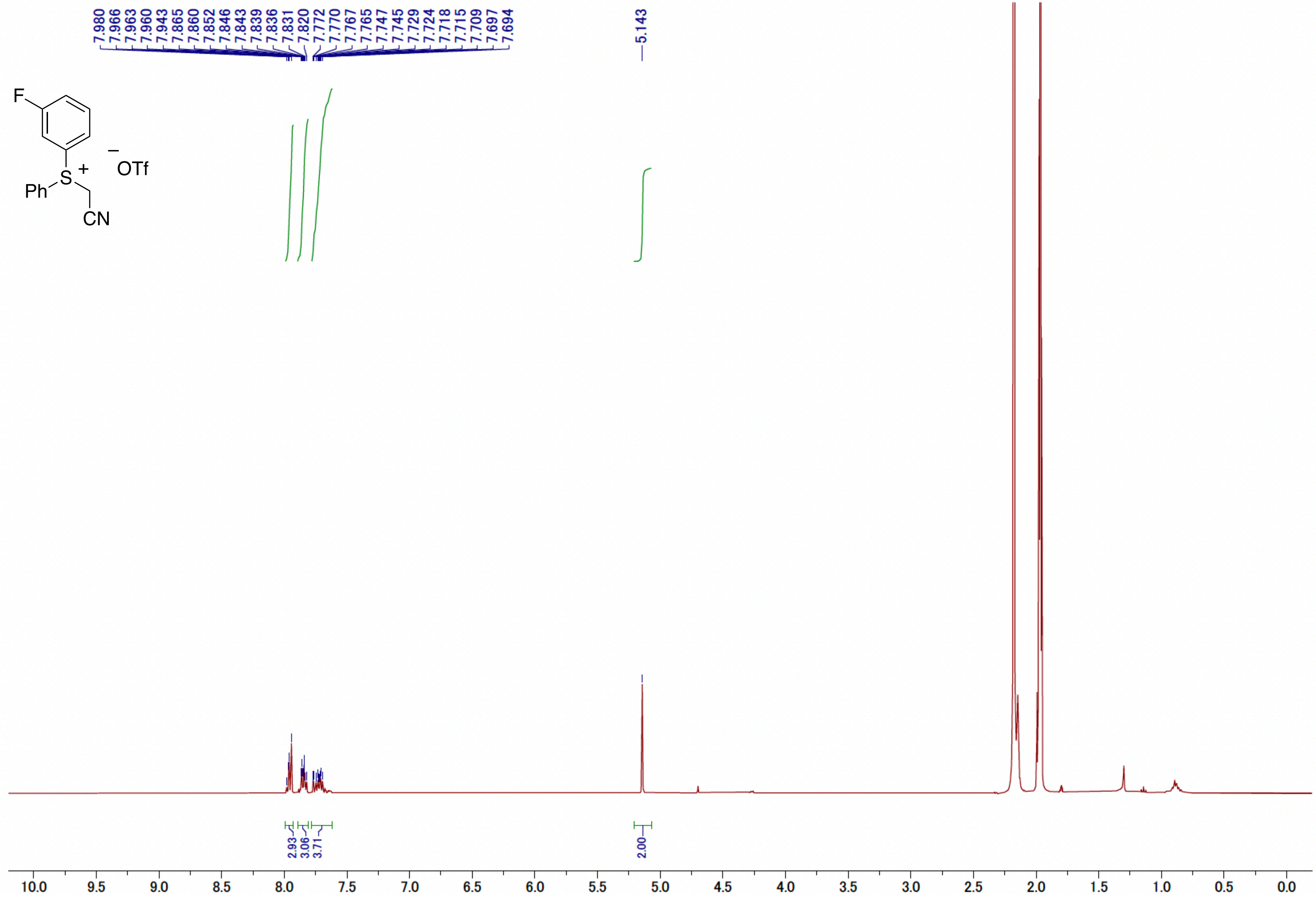

^{13}C NMR (100 MHz, CD_3CN) ; **3k**

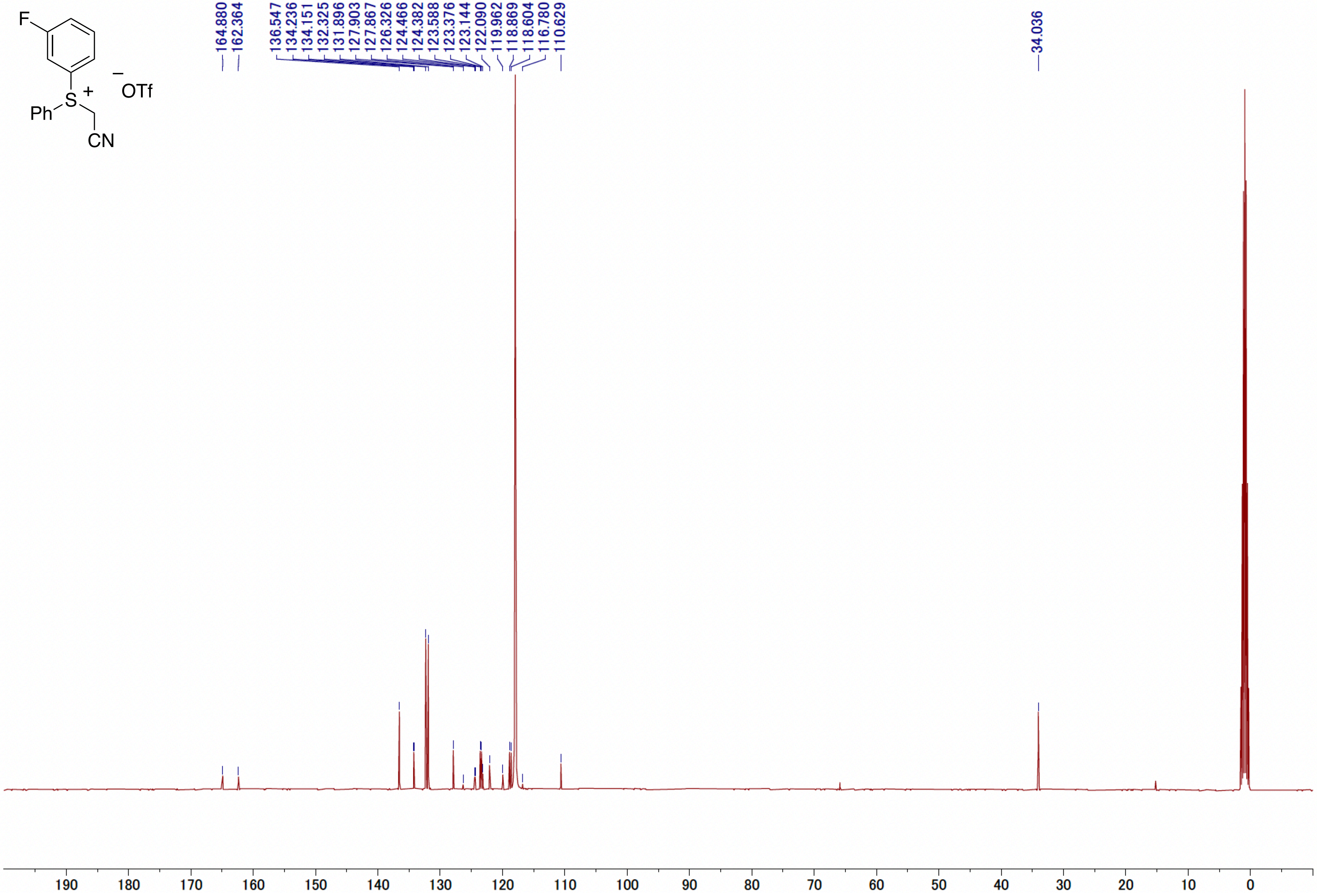

^{19}F NMR (376 MHz, CD_3CN) ; **3k**

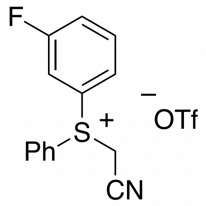

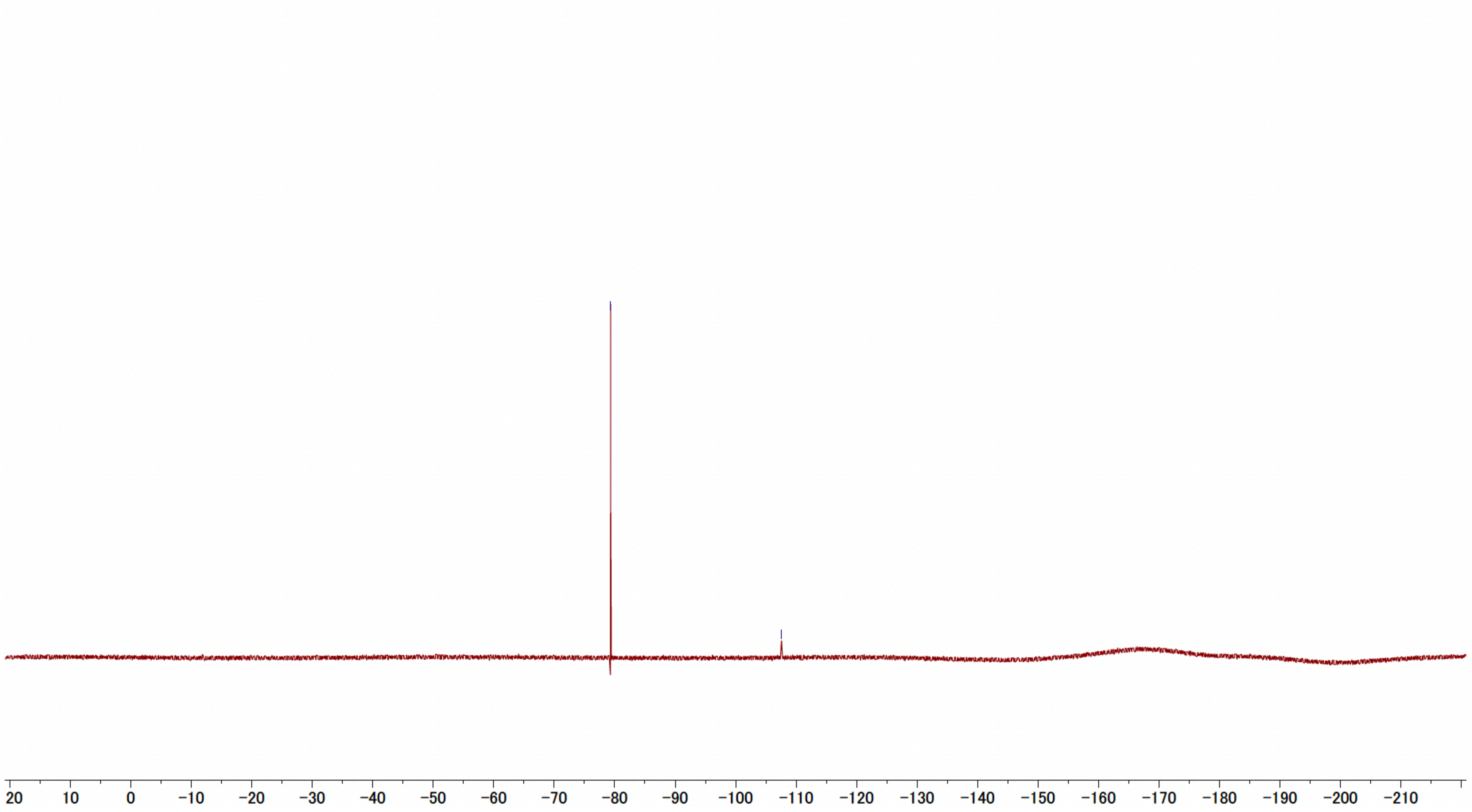

^1H NMR (400 MHz, $CDCl_3$) ; **3l**

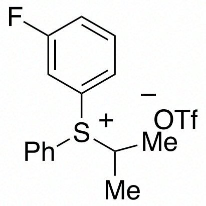

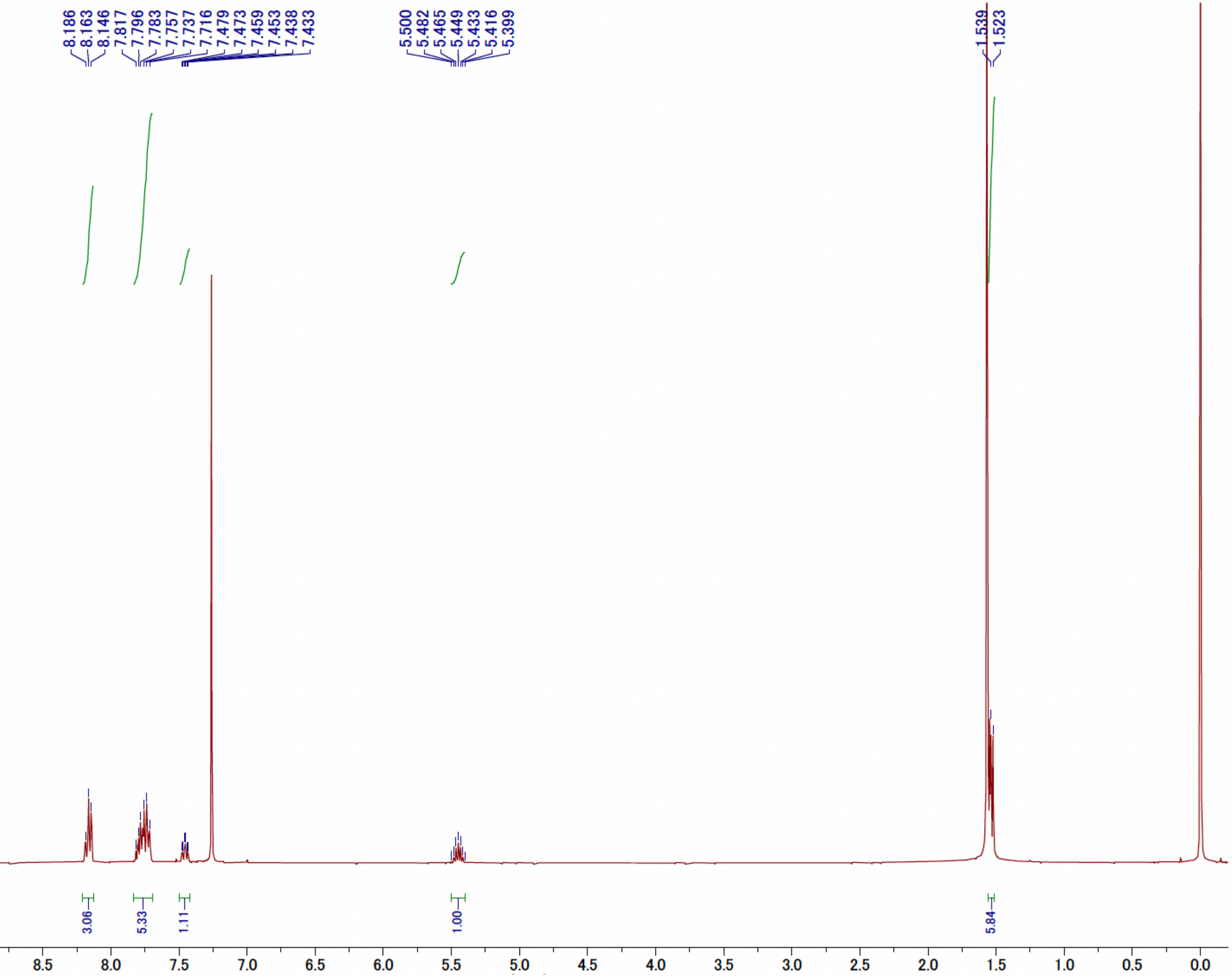

^{13}C NMR (100 MHz, CD_3CN) ; **3l**

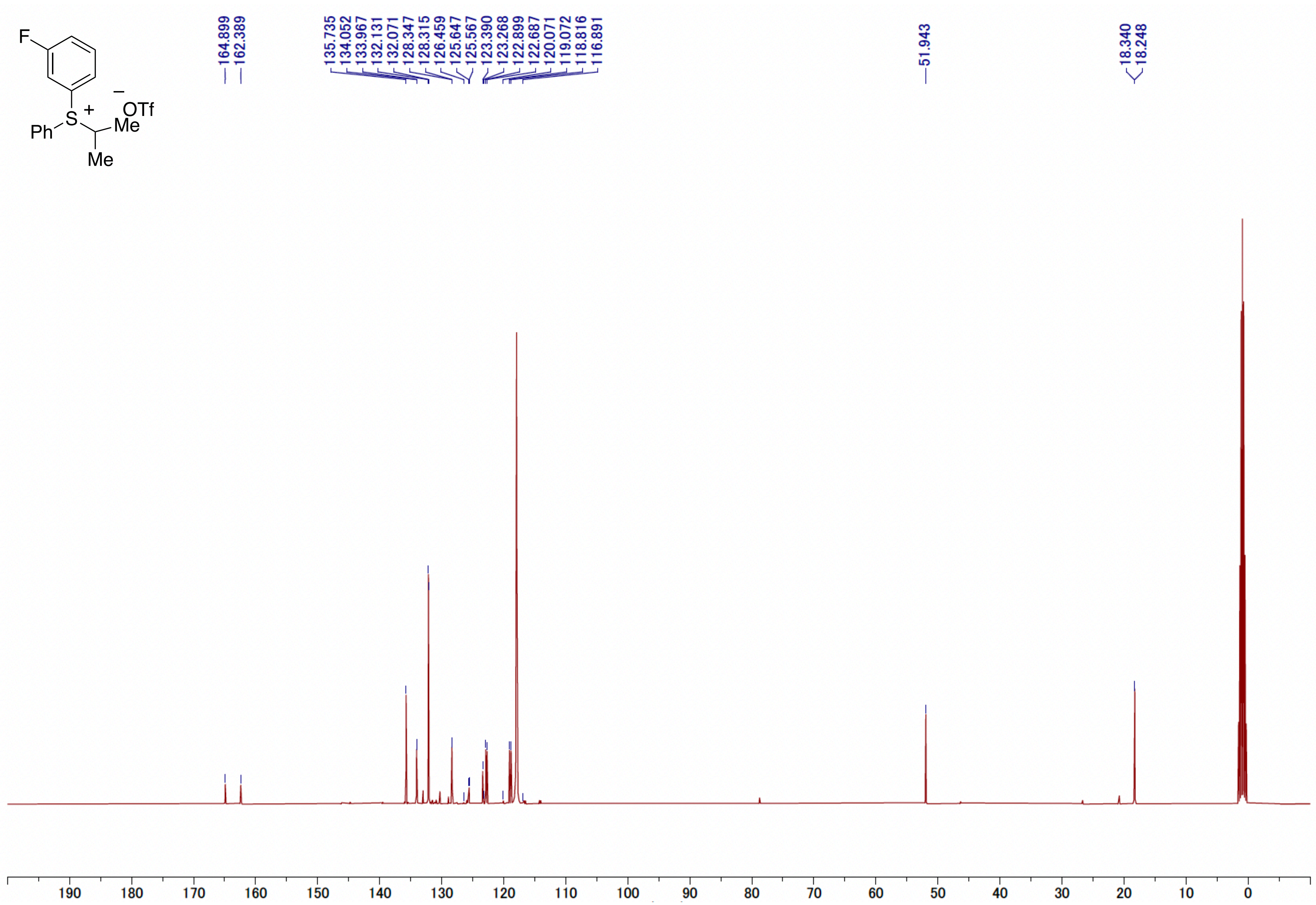

^{19}F NMR (376 MHz, CD_3CN) ; **3l**

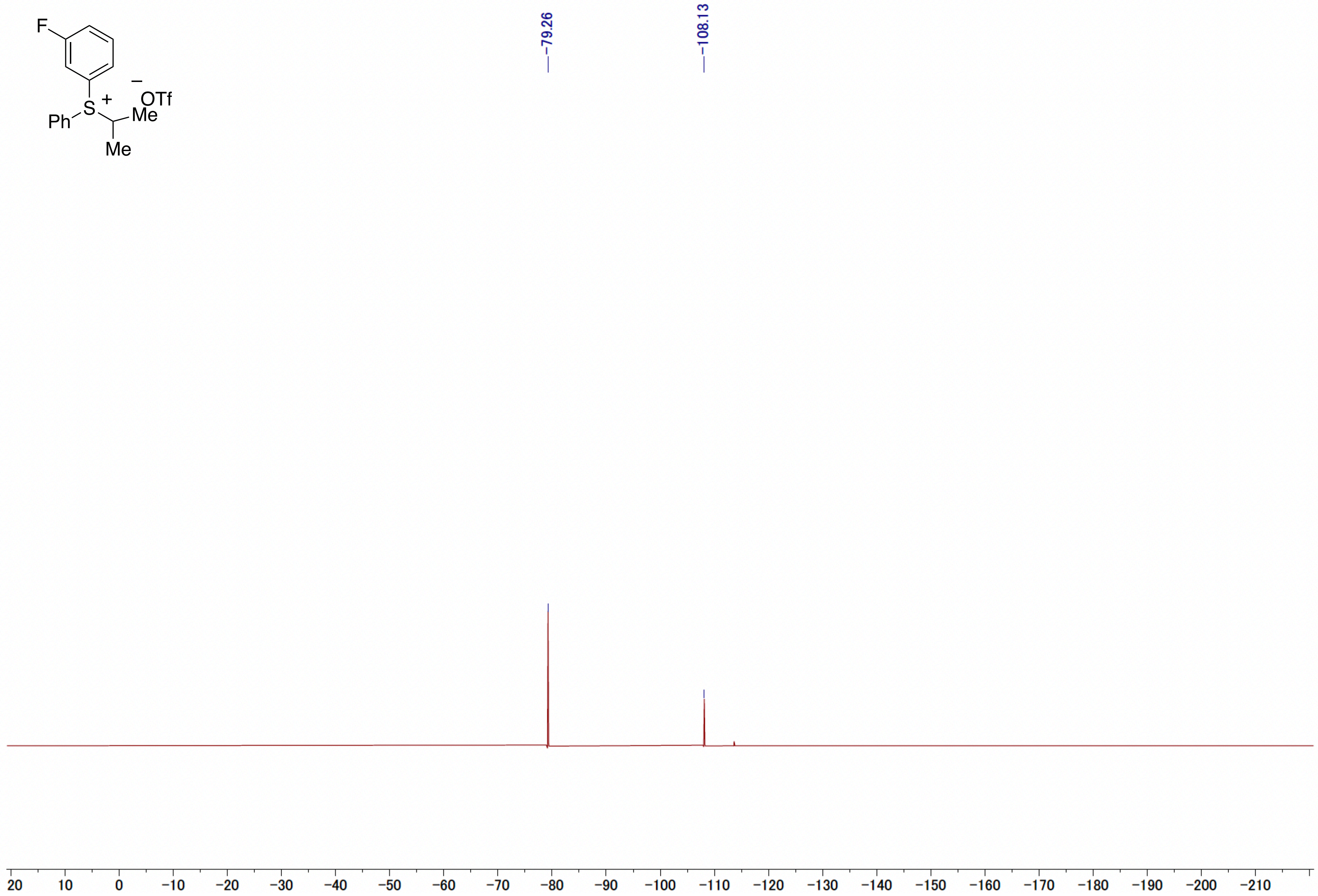

^{1}H NMR (400 MHz, $CDCl_3$) ; **3m**

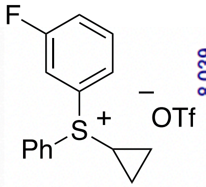

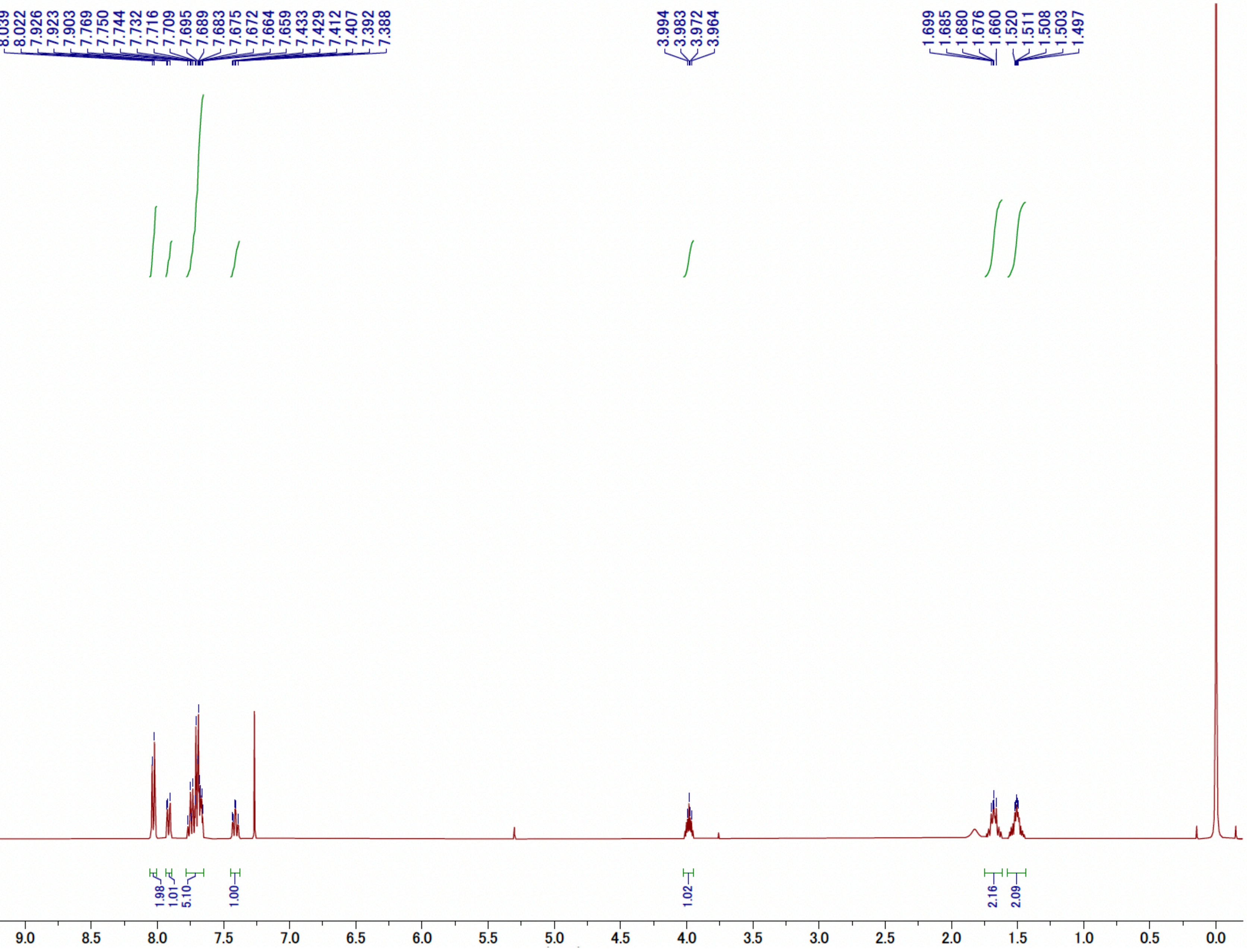

^{13}C NMR (100 MHz, CD_3CN) ; **3m**

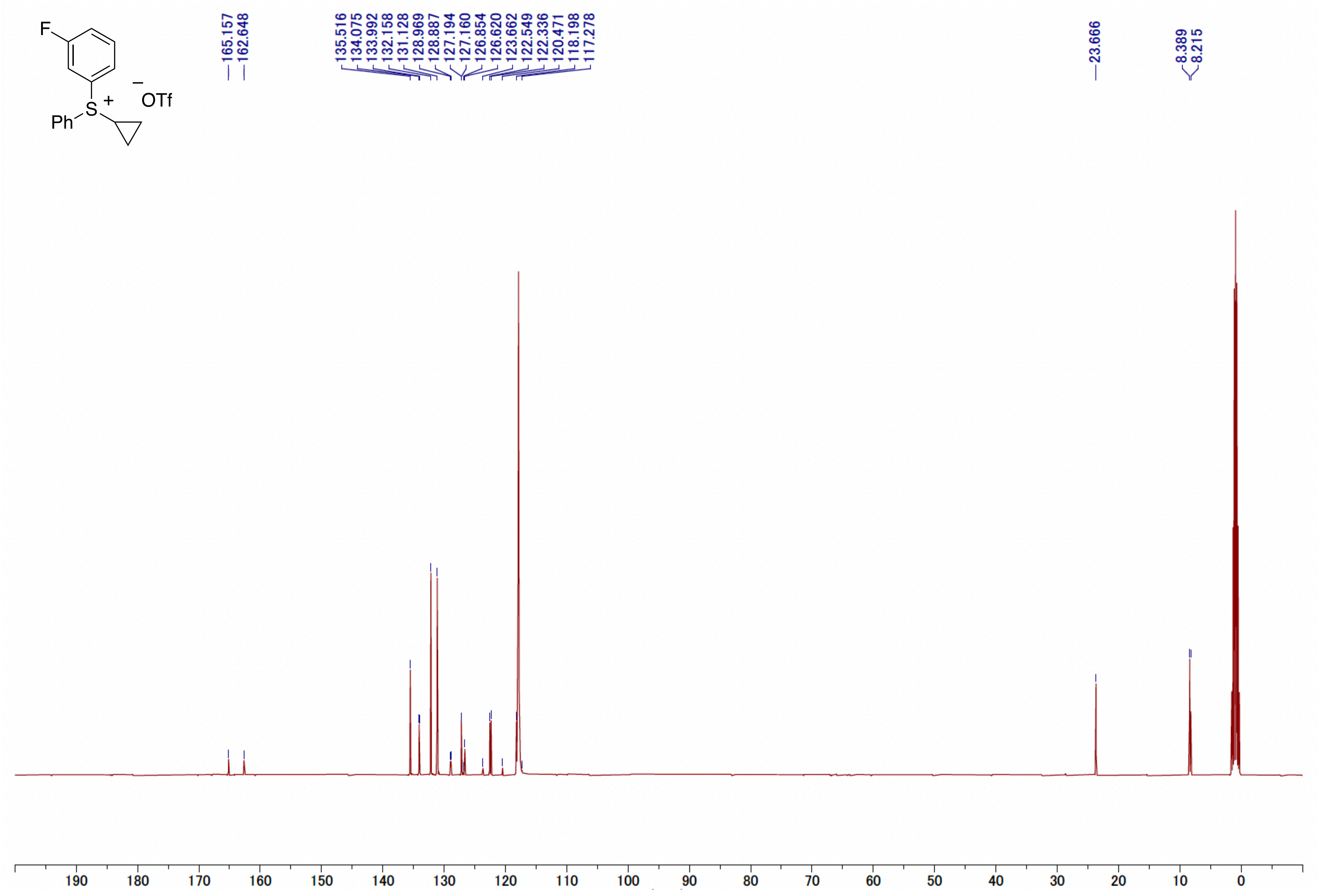

^{19}F NMR (376 MHz, $CDCl_3$) ; **3m**

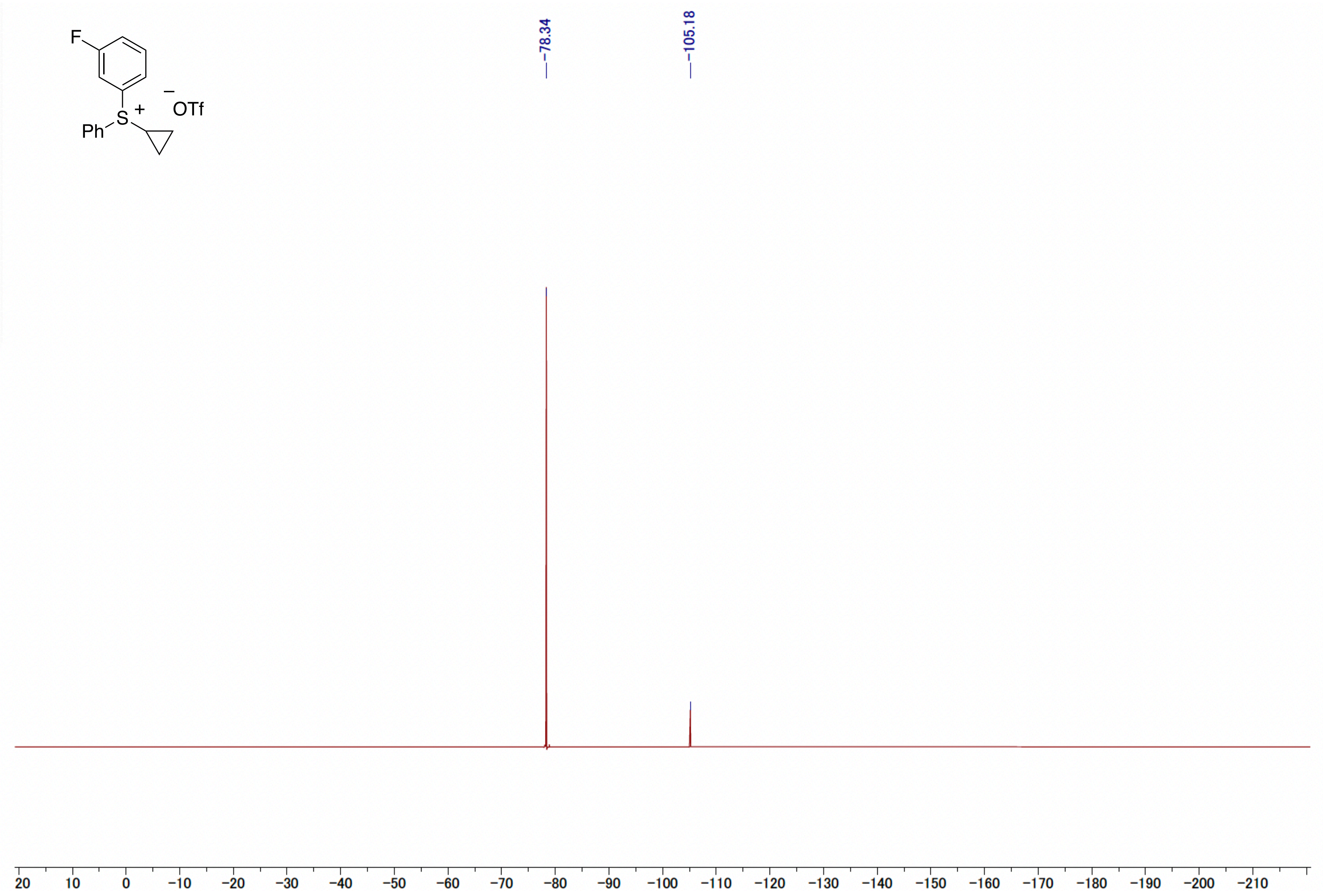

^{1}H NMR (400 MHz, $CDCl_3$) ; **3n**

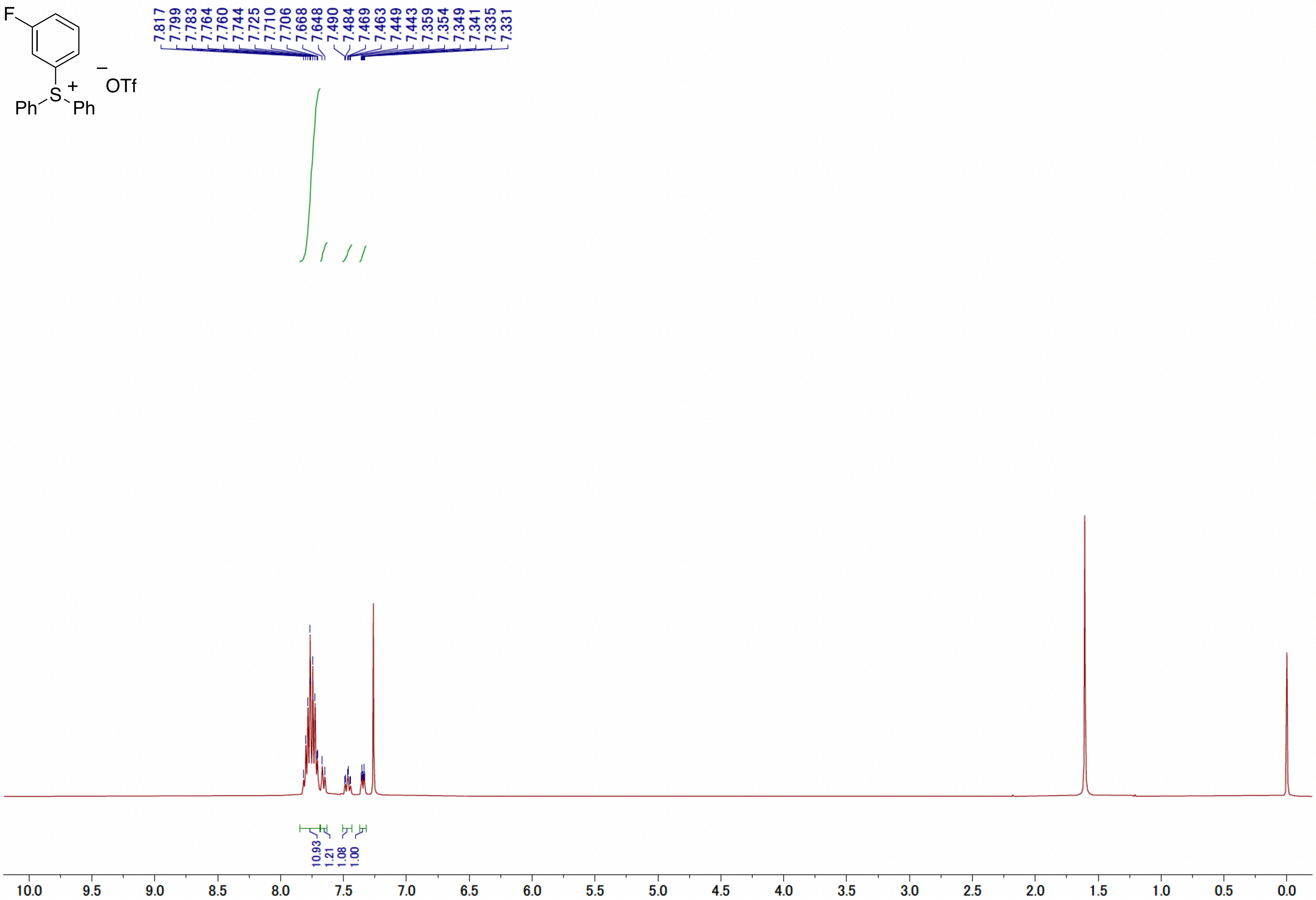

^{13}C NMR (100 MHz, $CDCl_3$) ; **3n**

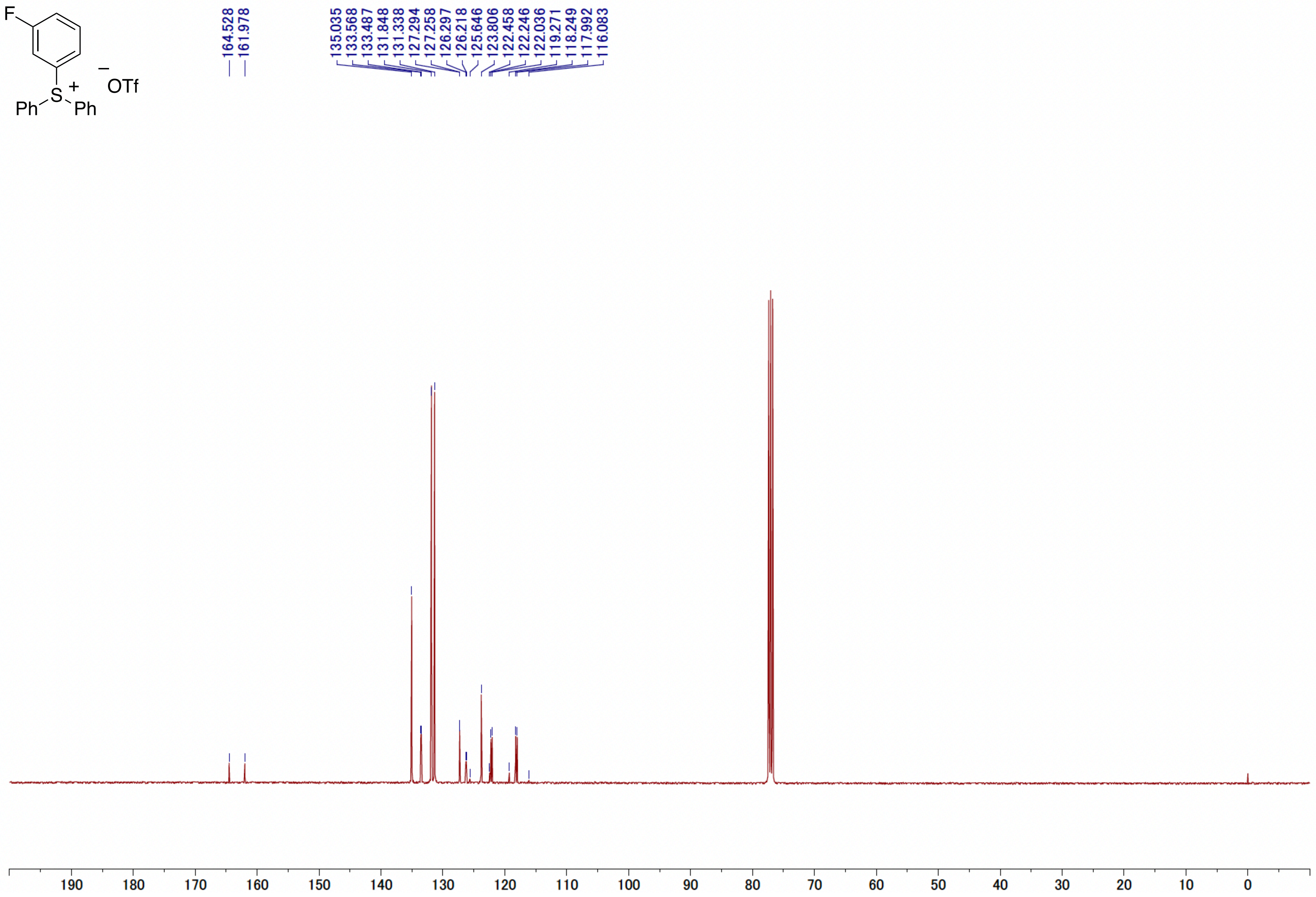

^{19}F NMR (376 MHz, $CDCl_3$) ; **3n**

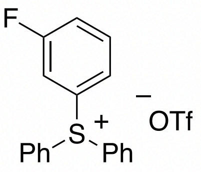

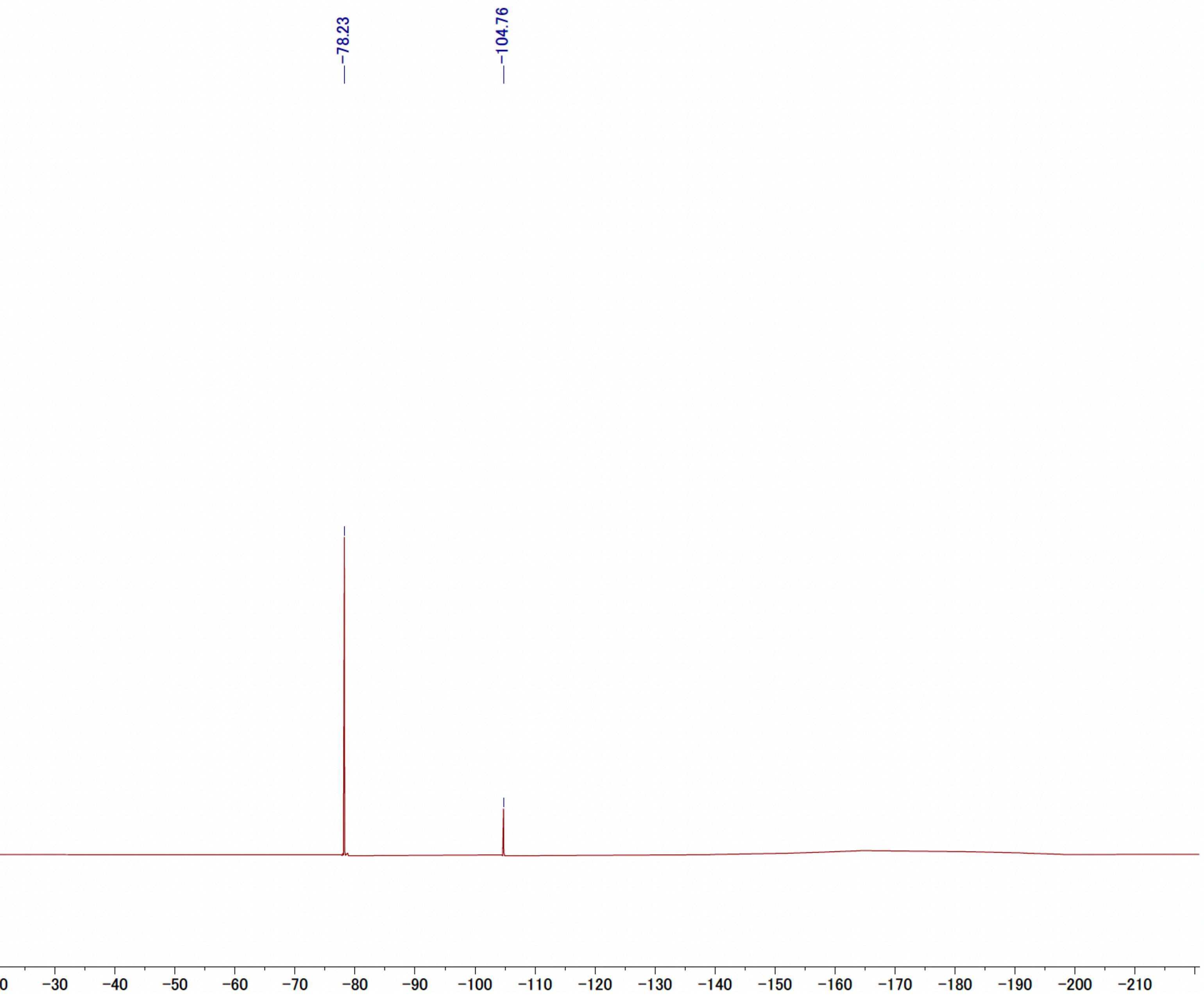

^{1}H NMR (400 MHz, $CDCl_3$) ; **3o**

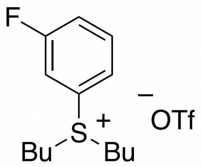

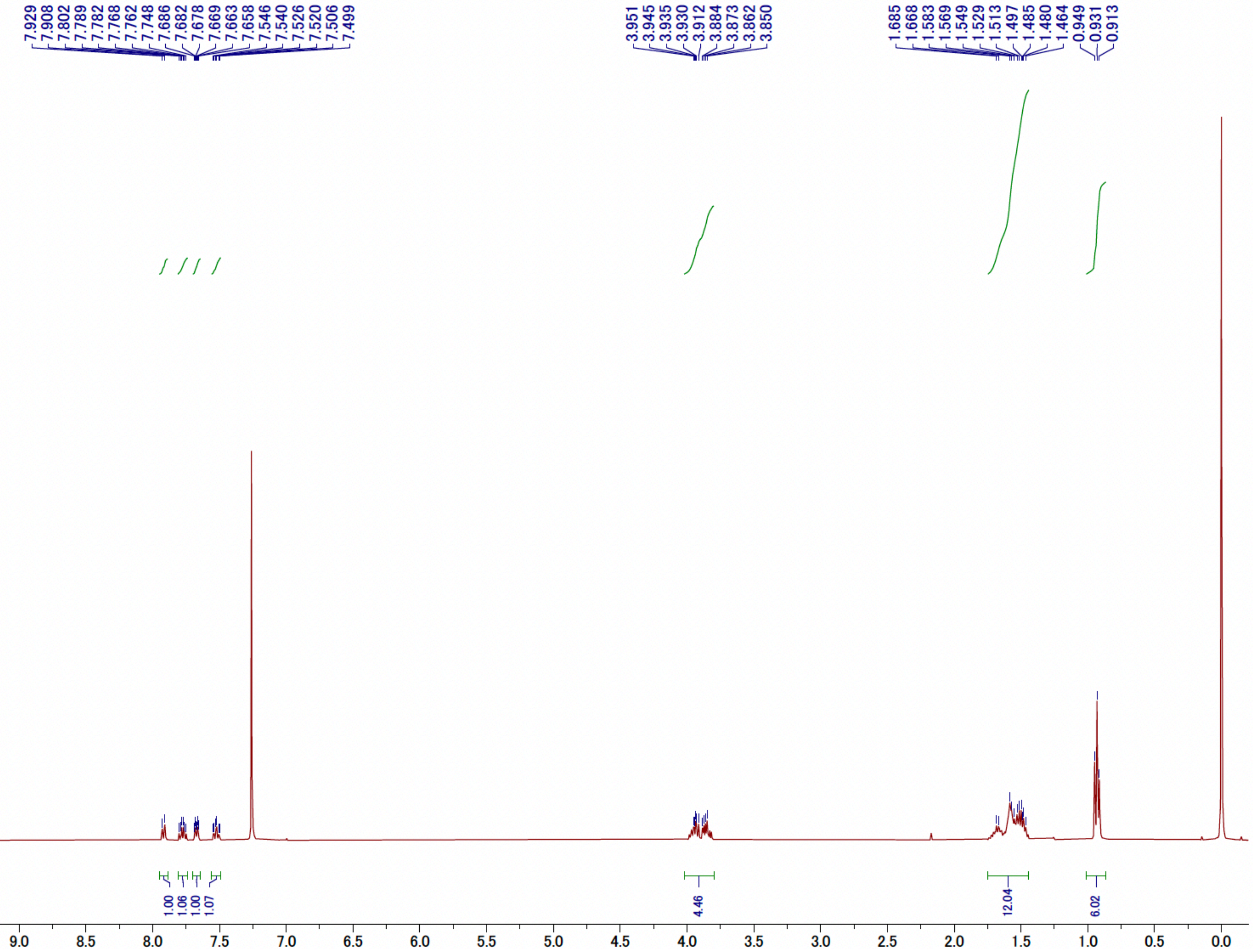

^{13}C NMR (100 MHz, CD_3CN) ; **3o**

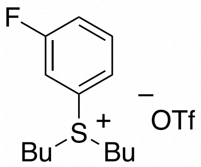

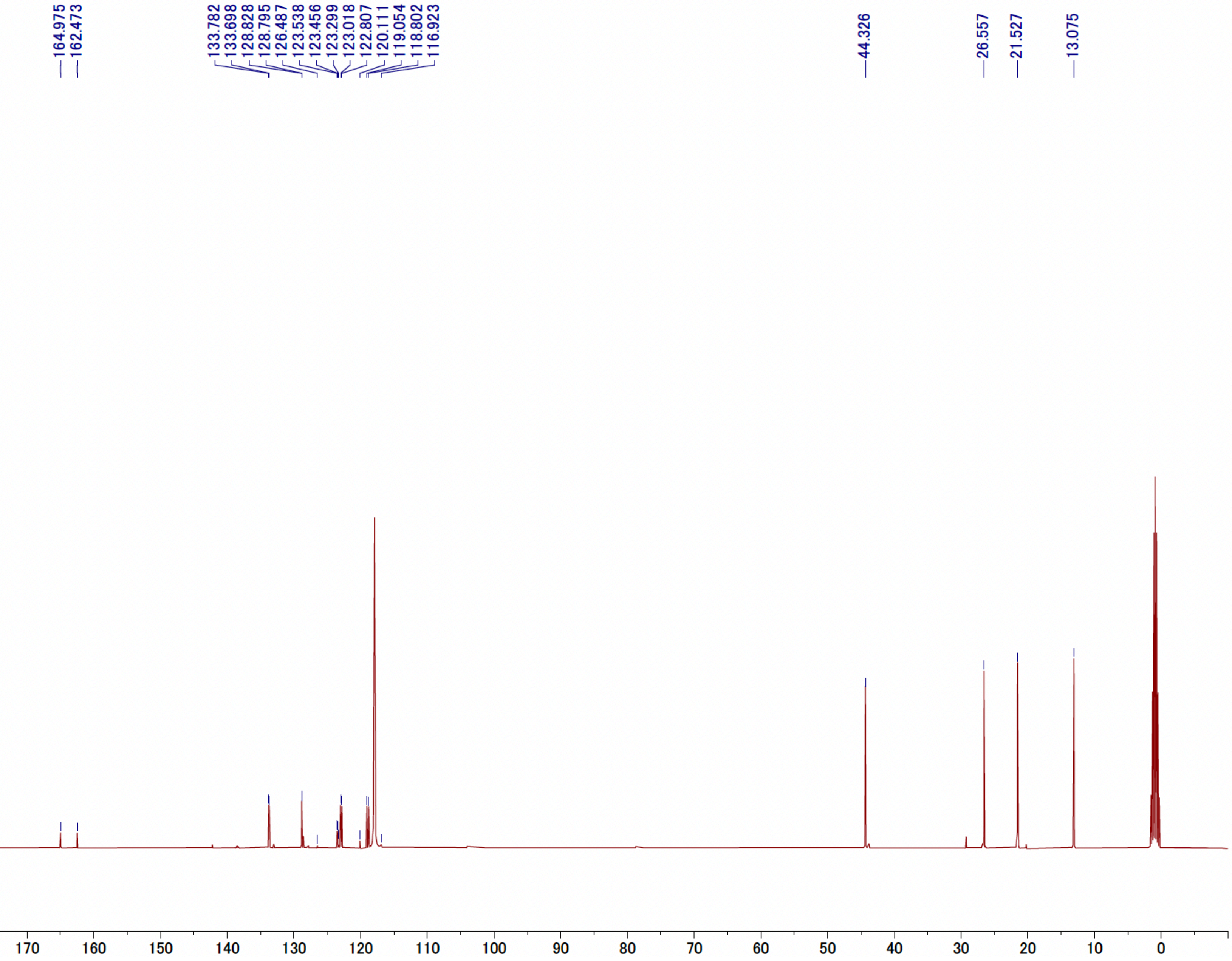

^{19}F NMR (376 MHz, CD_3CN) ; **3o**

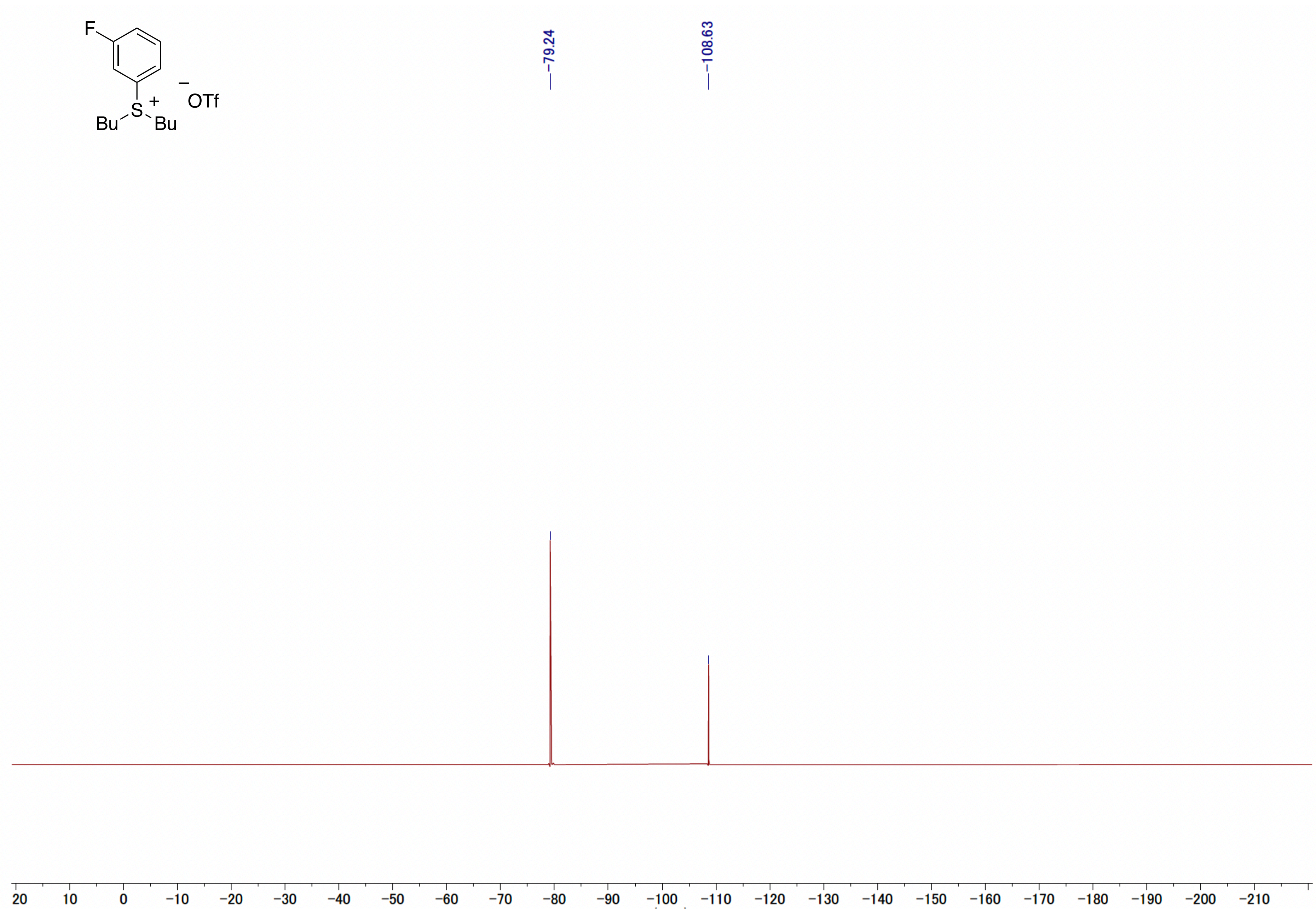

^{1}H NMR (400 MHz, $CDCl_3$) ; **3p**

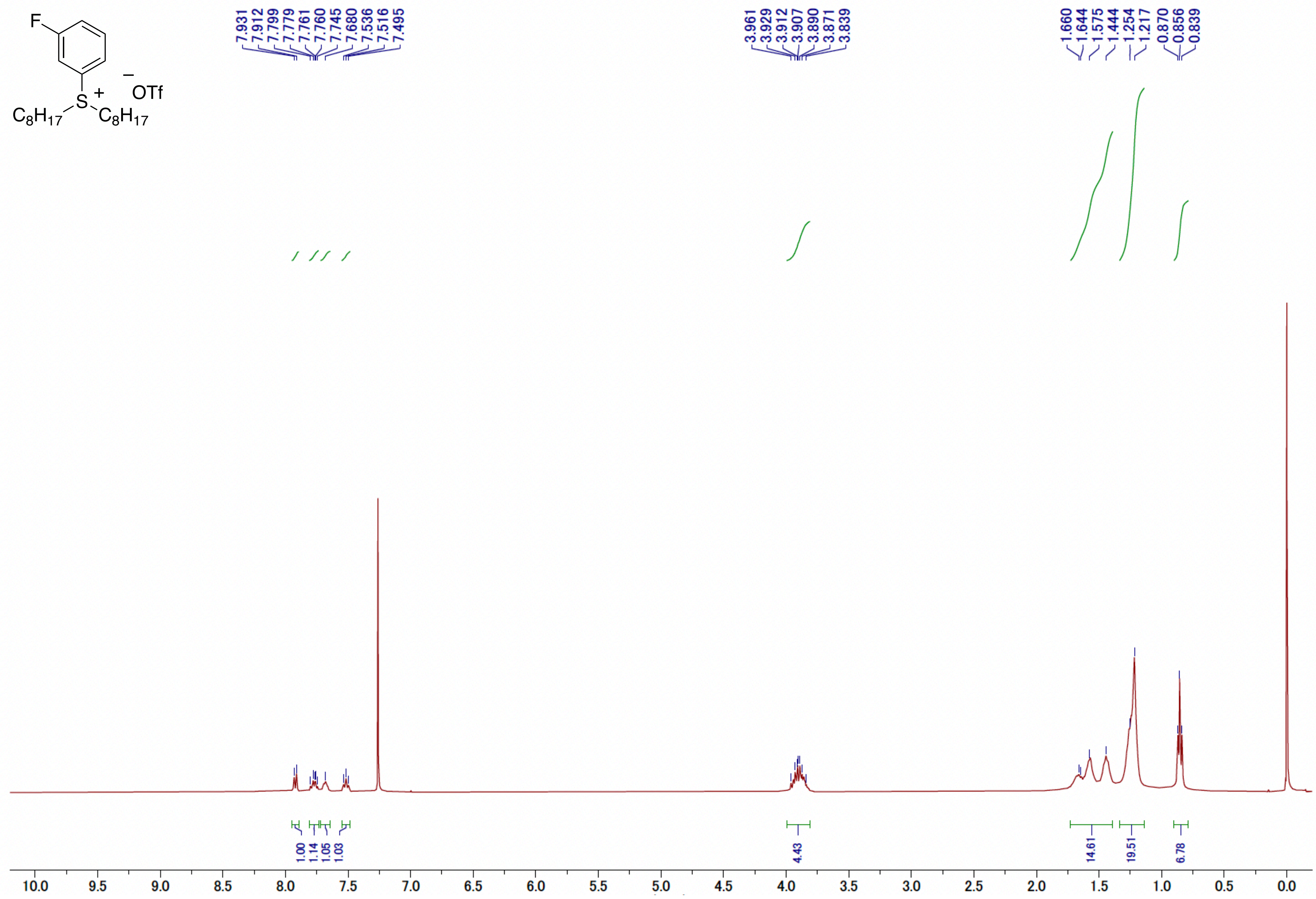

^{13}C NMR (100 MHz, CD_3CN) ; **3p**

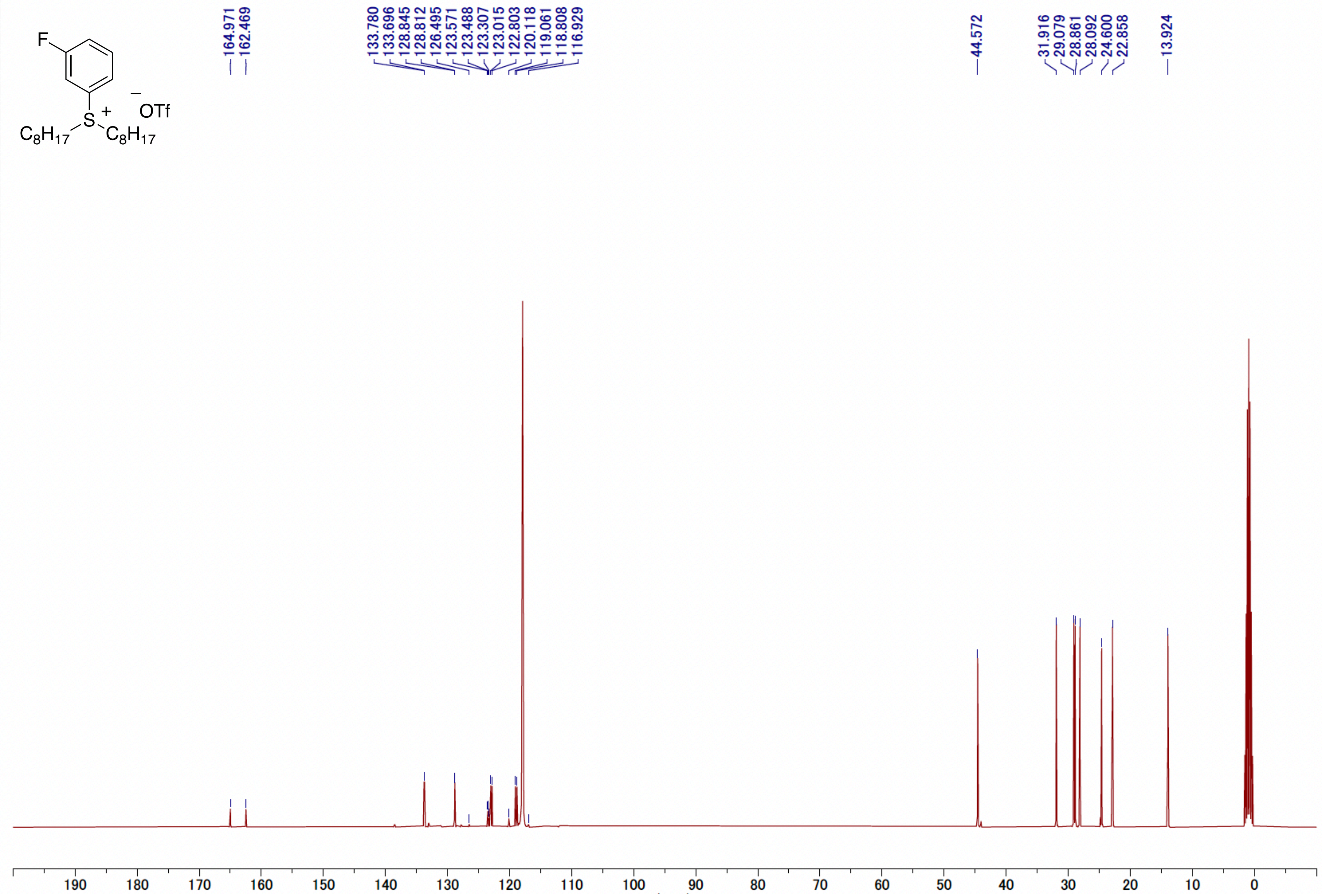

^{19}F NMR (376 MHz, CD_3CN) ; **3p**

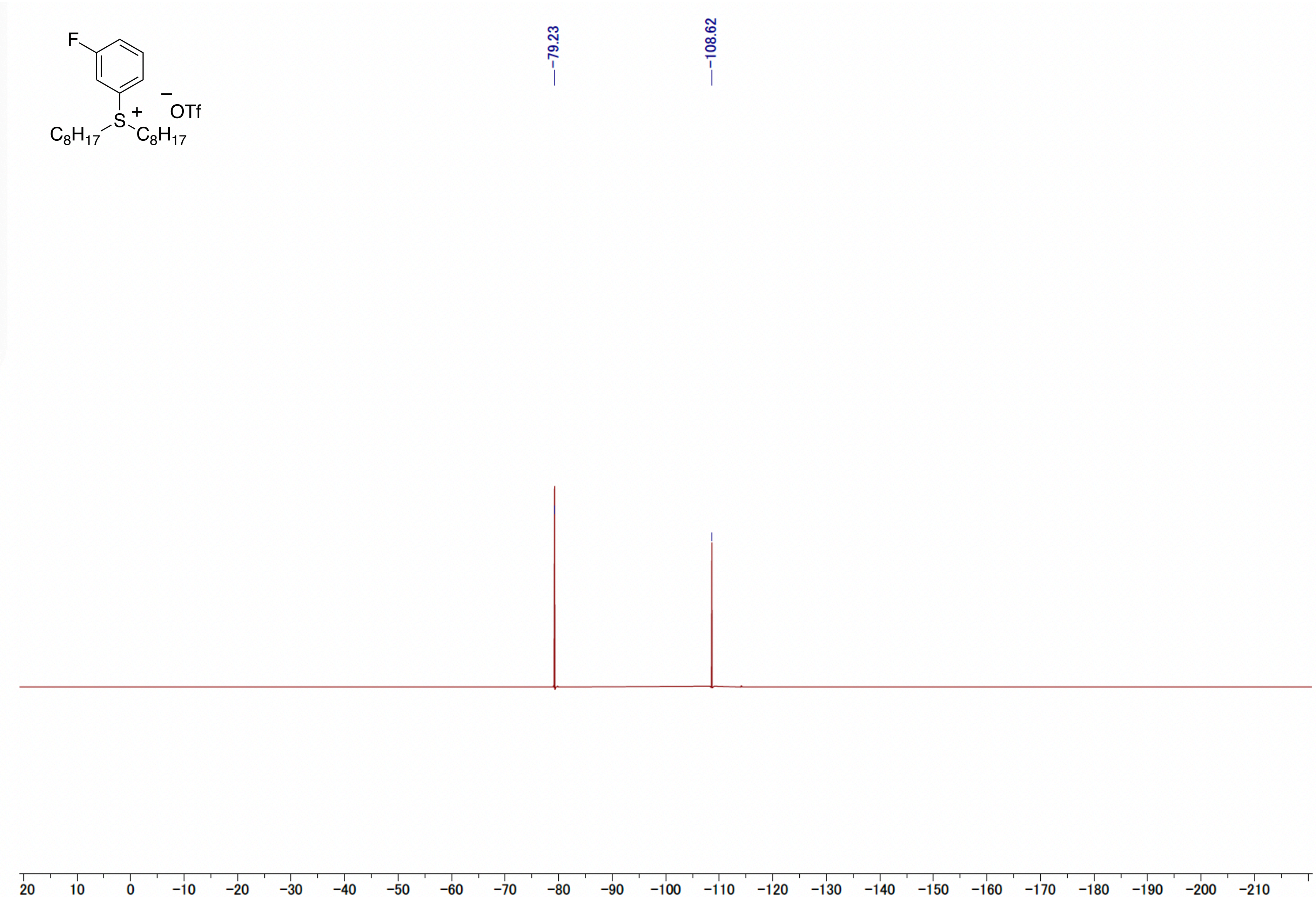

^{1}H NMR (400 MHz, CD_3CN) ; **3q**

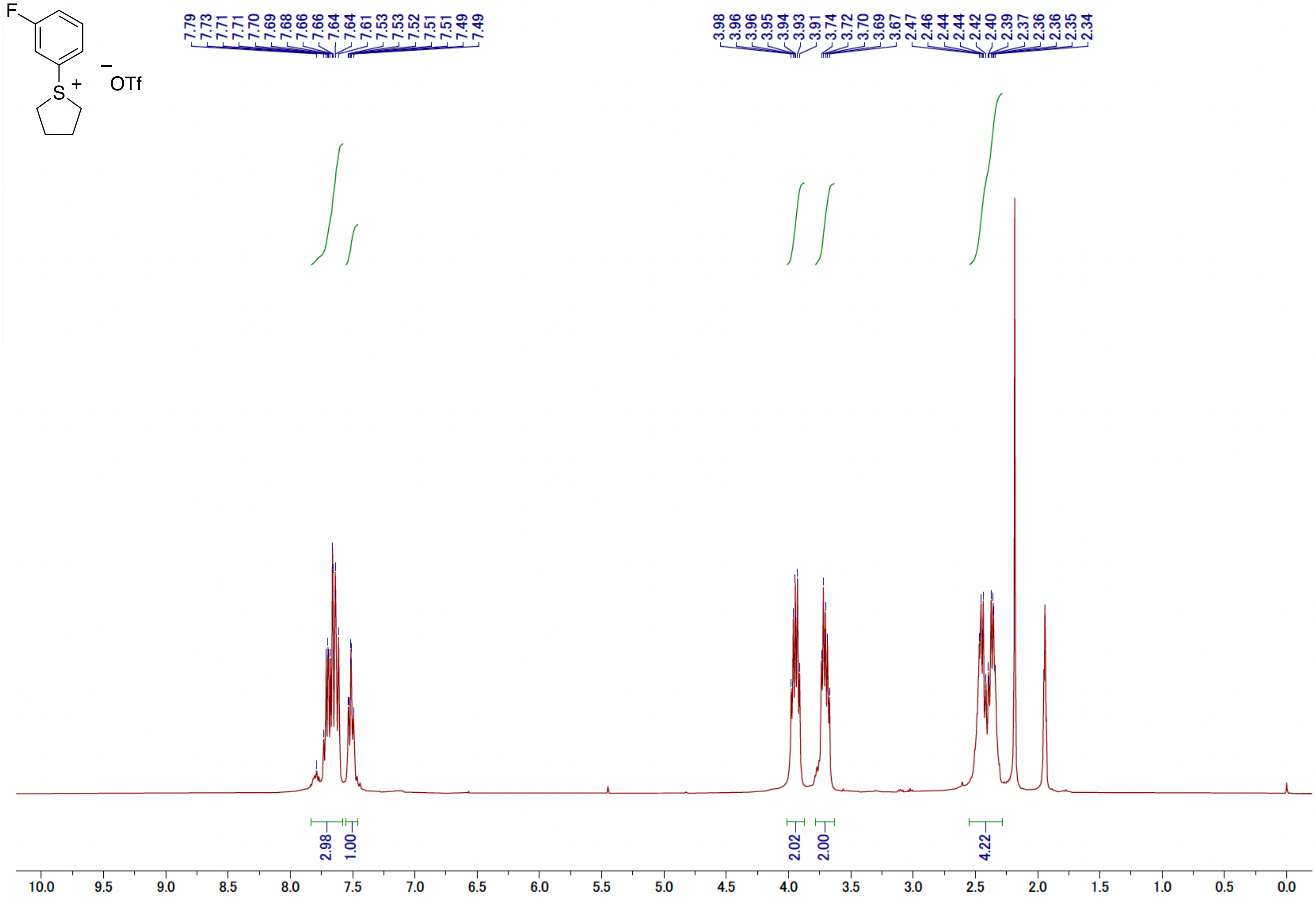

^{13}C NMR (100 MHz, CD_3CN) ; **3q**

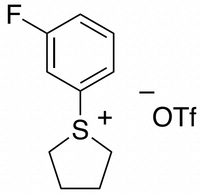

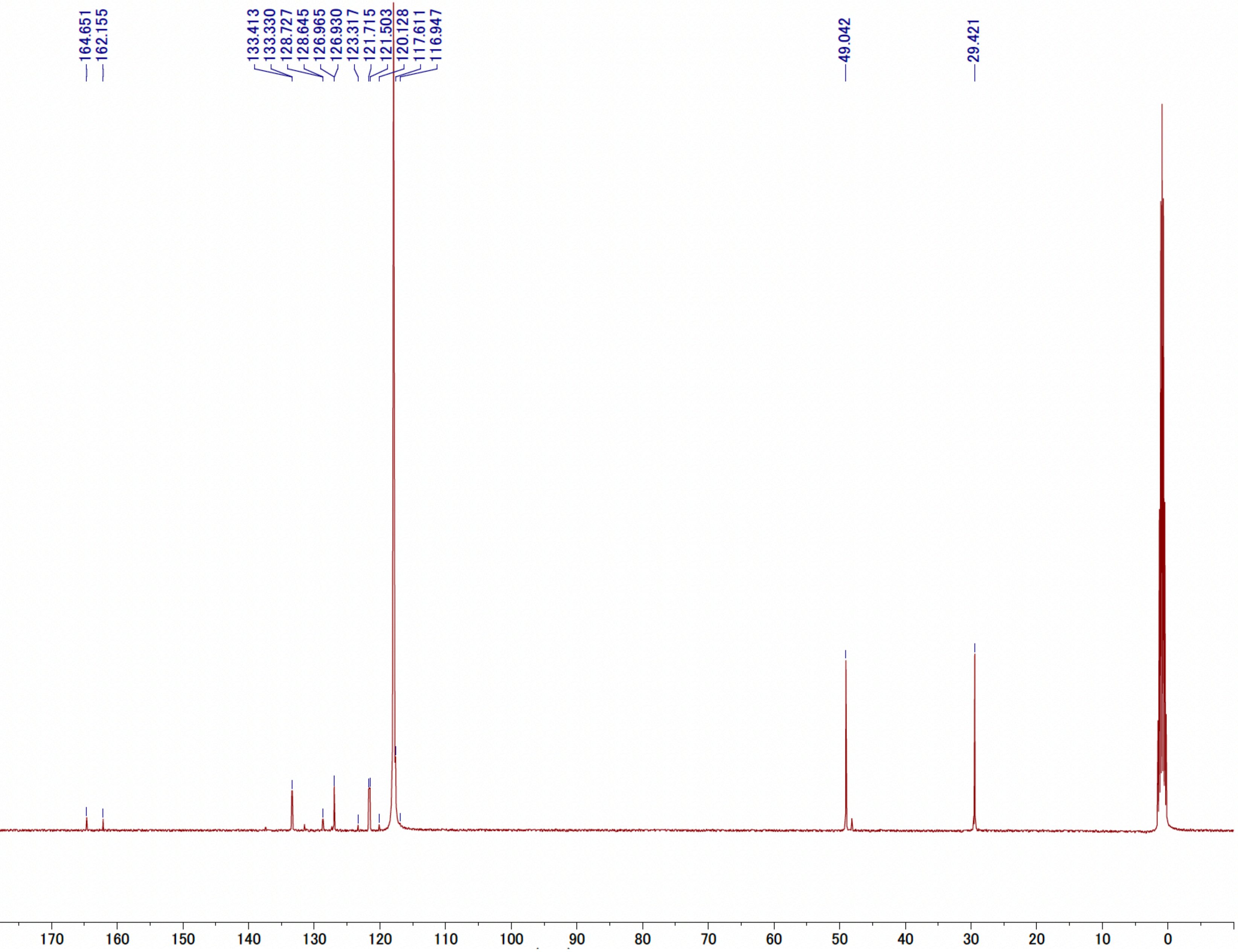

^{19}F NMR (376 MHz, CD_3CN) ; **3q**

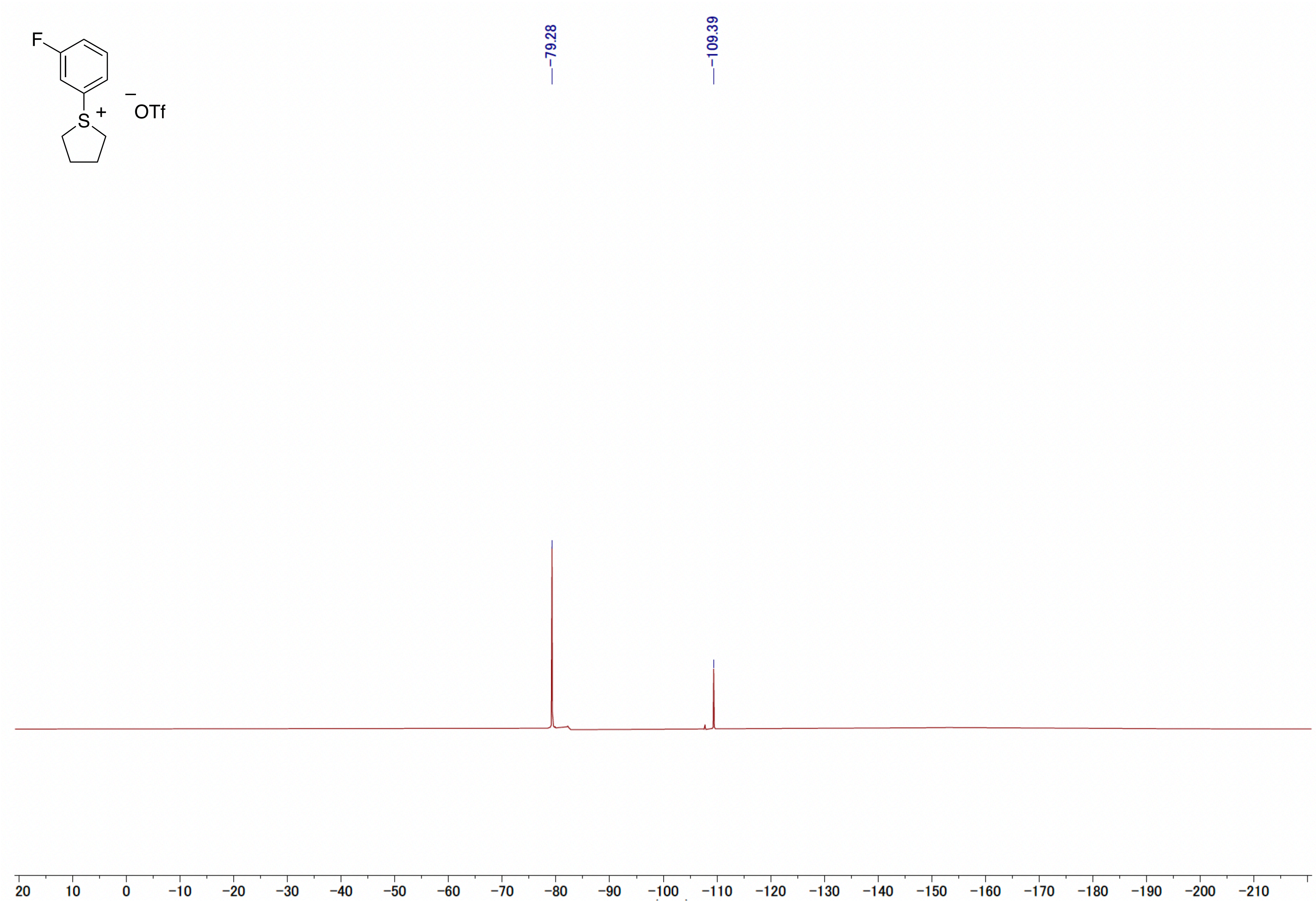

^{1}H NMR (400 MHz, $CDCl_3$) ; **3r**

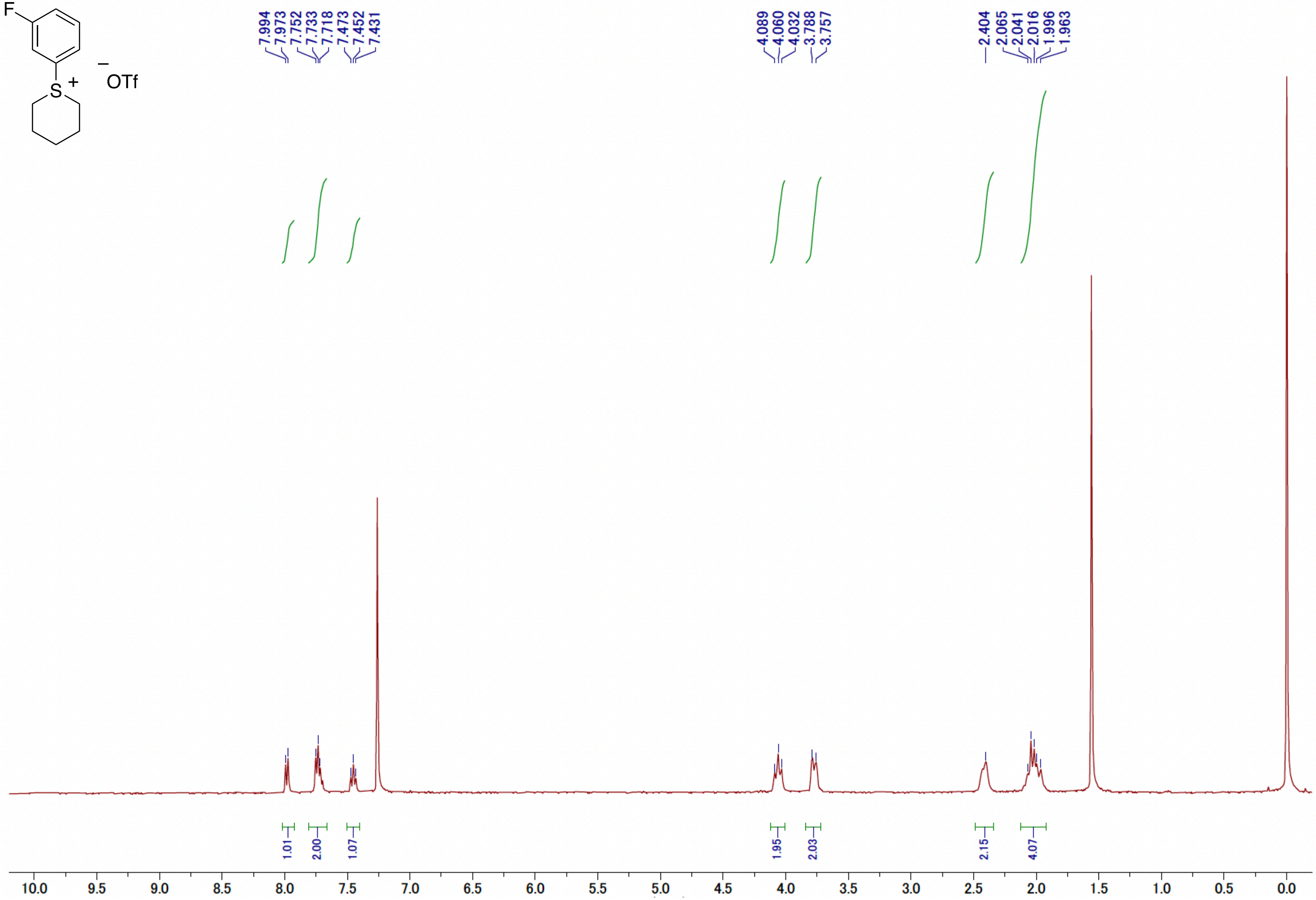

^{13}C NMR (100 MHz, CD_3CN) ; **3r**

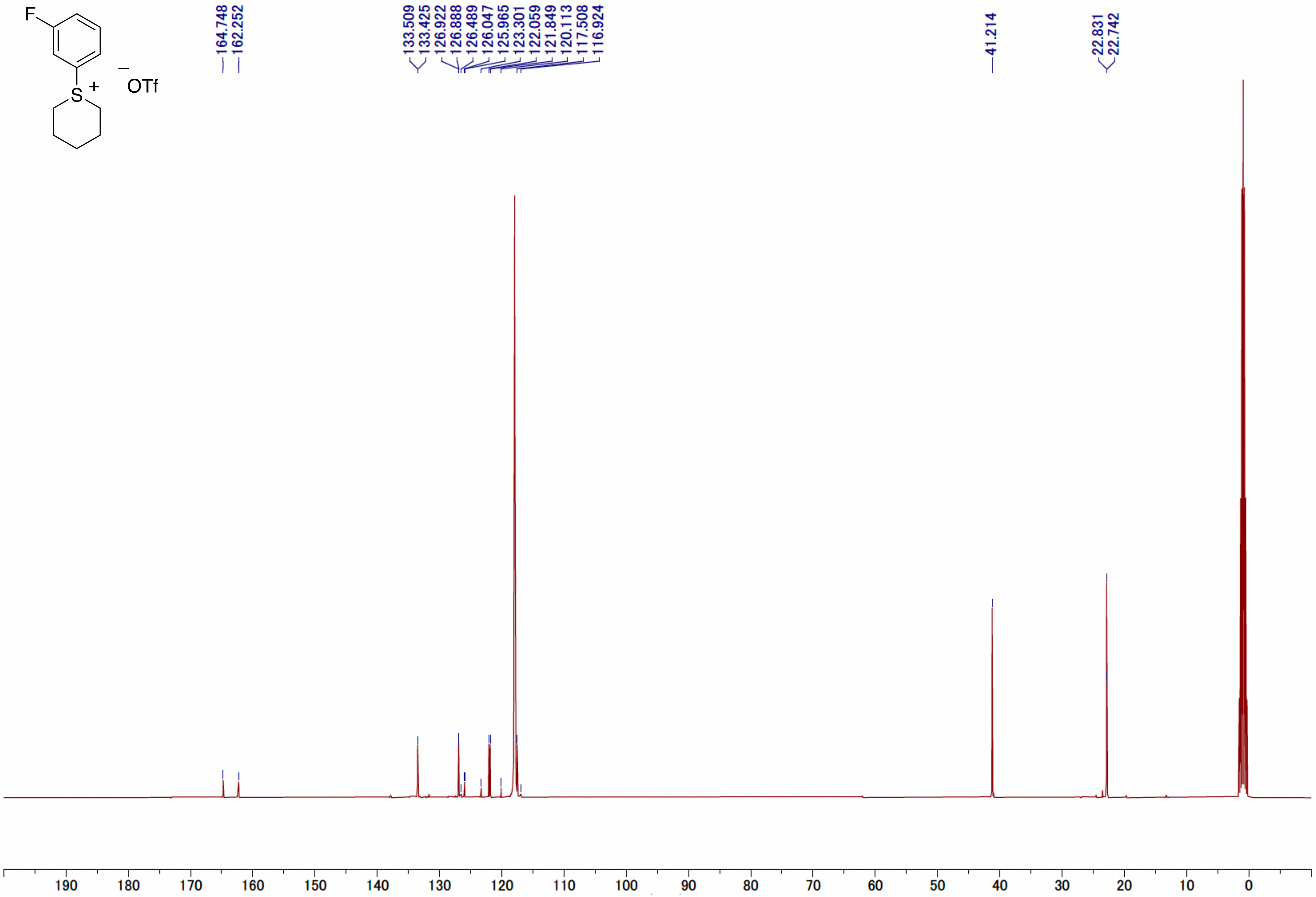

^{19}F NMR (376 MHz, CD_3CN) ; **3r**

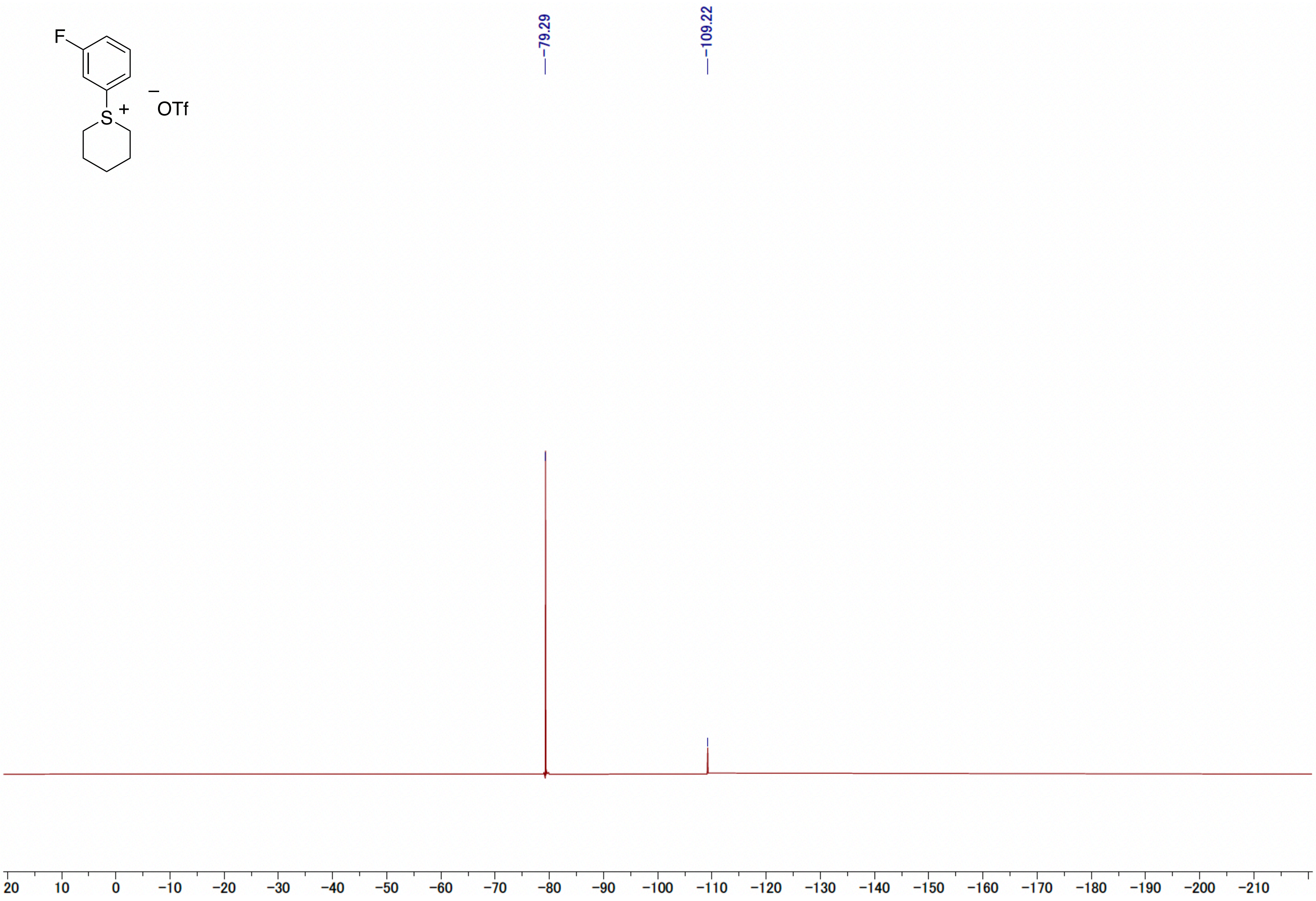

^{1}H NMR (400 MHz, $CDCl_3$) ; **3s**

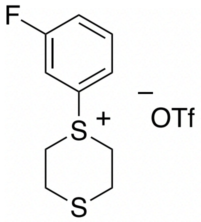

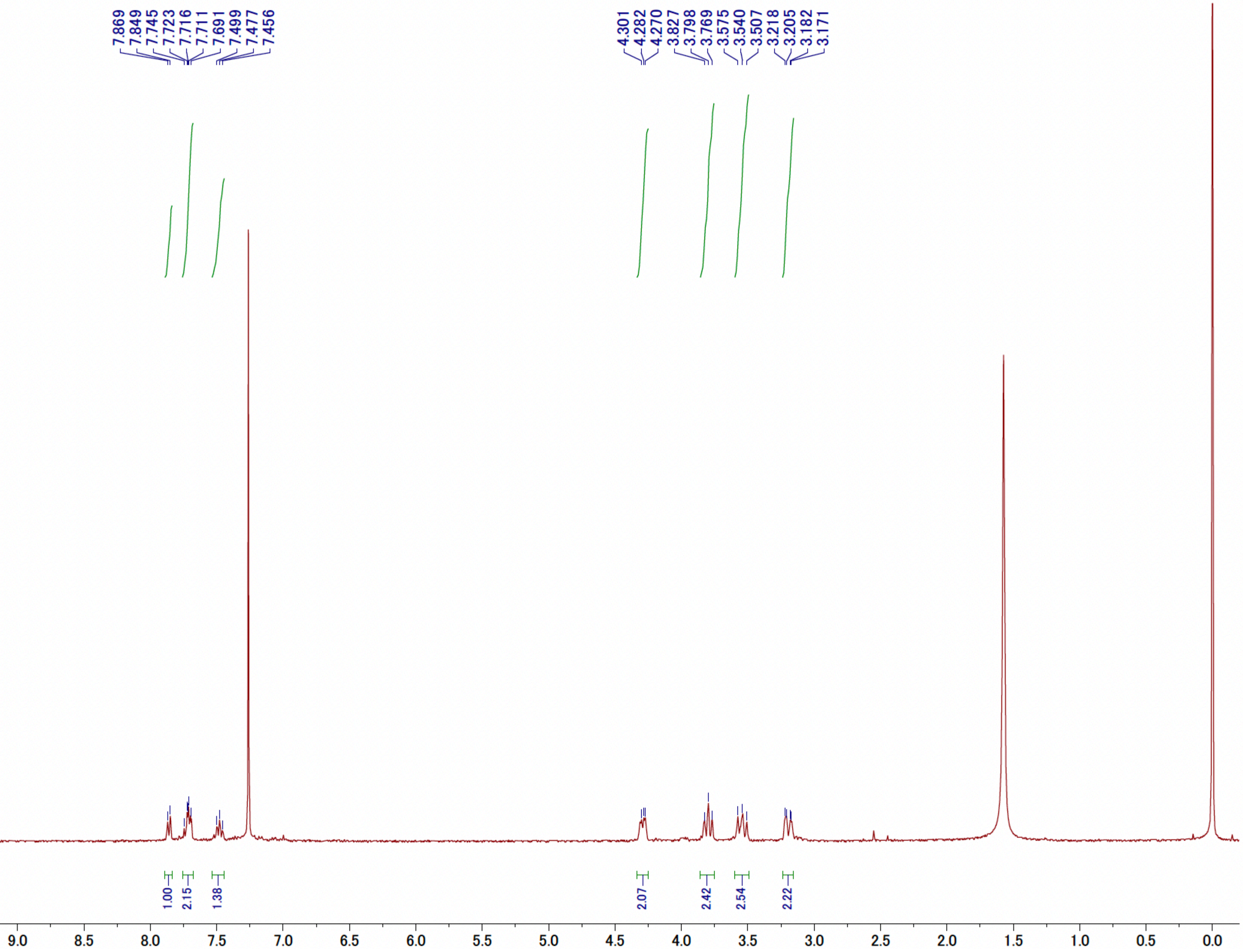

^{13}C NMR (100 MHz, CD_3CN) ; **3s**

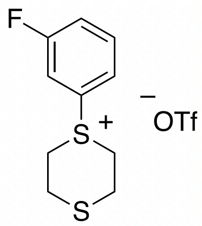

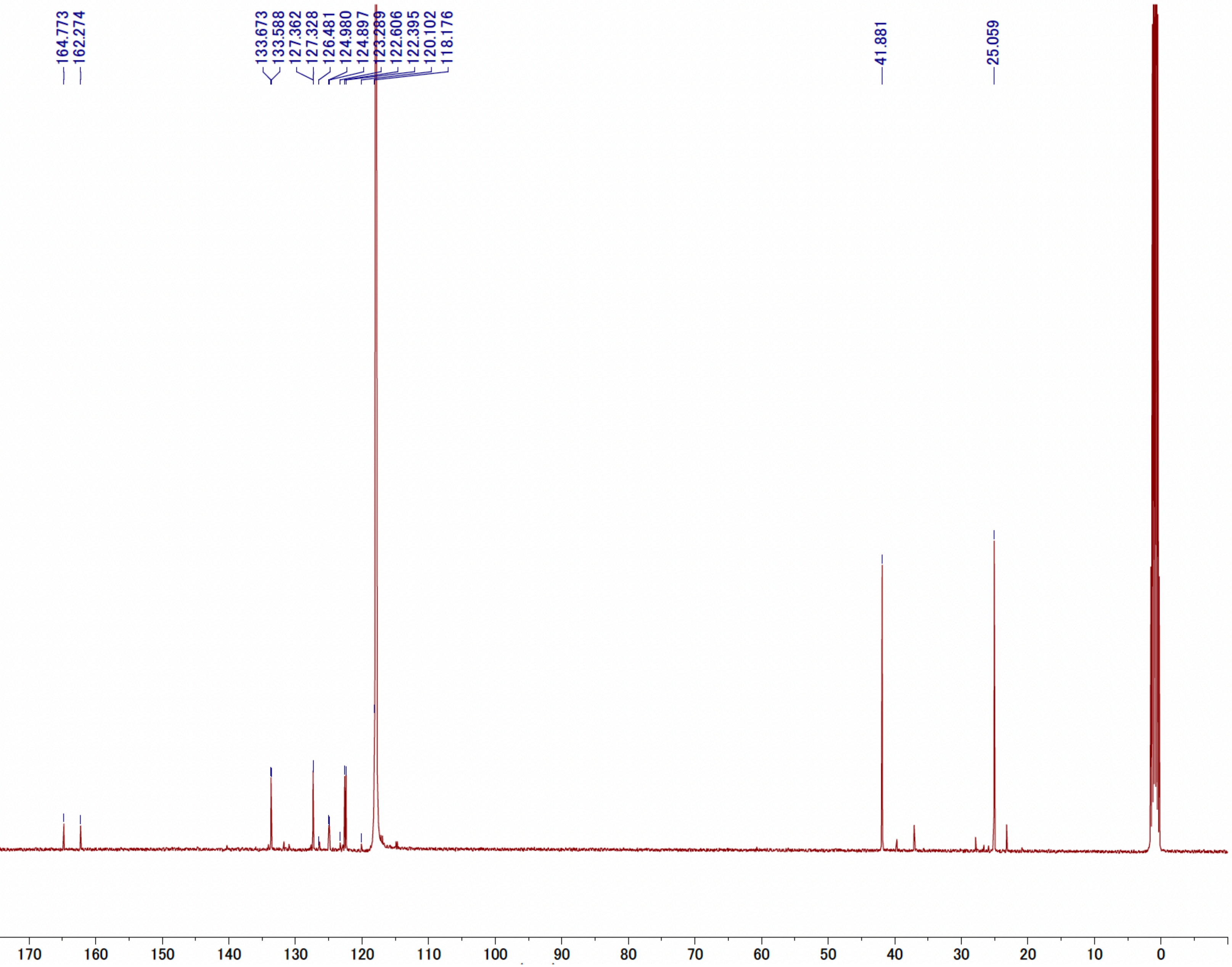

^{19}F NMR (376 MHz, CD_3CN) ; **3s**

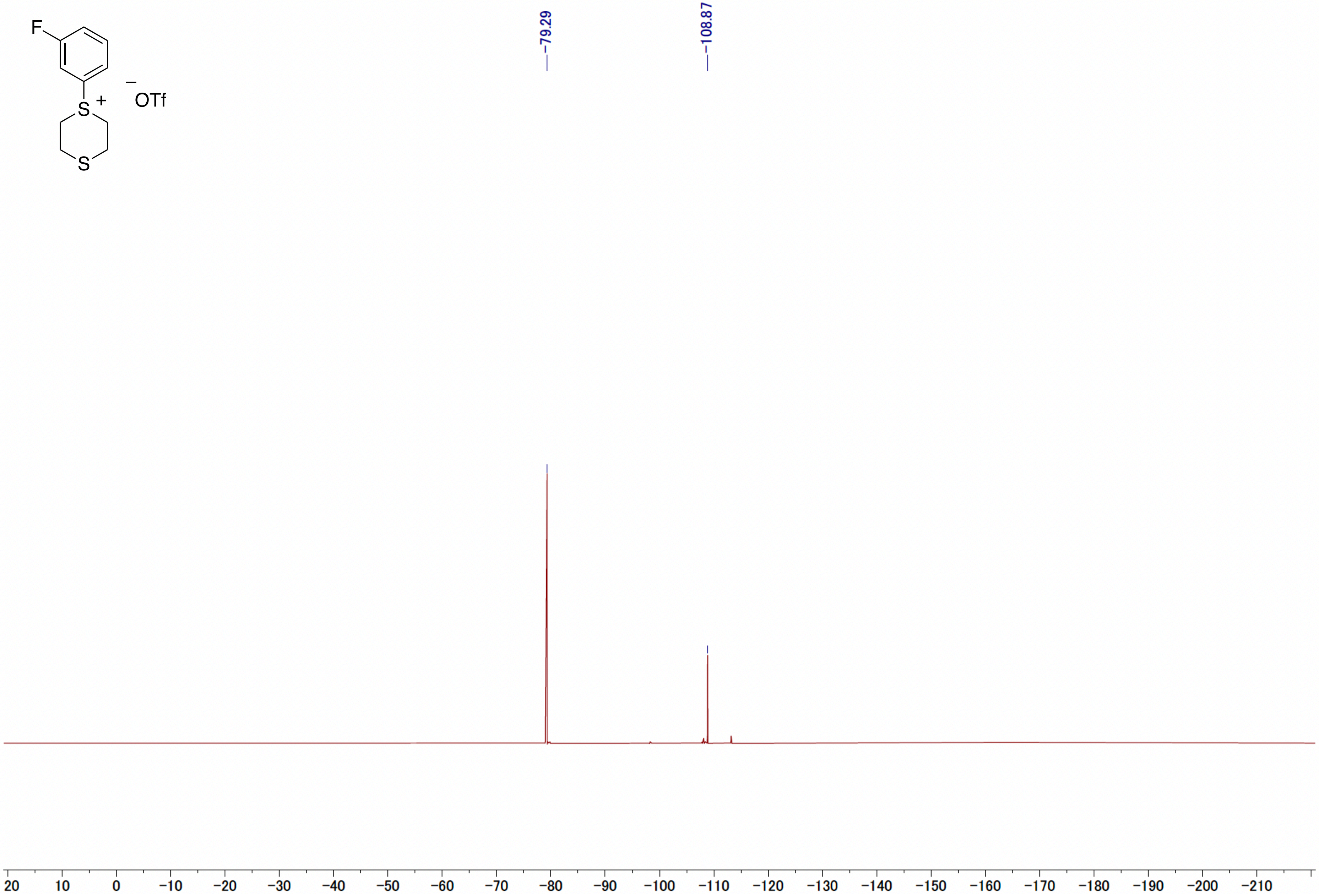

^{1}H NMR (400 MHz, $CDCl_3$) ; **3t**

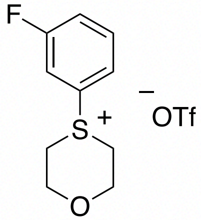

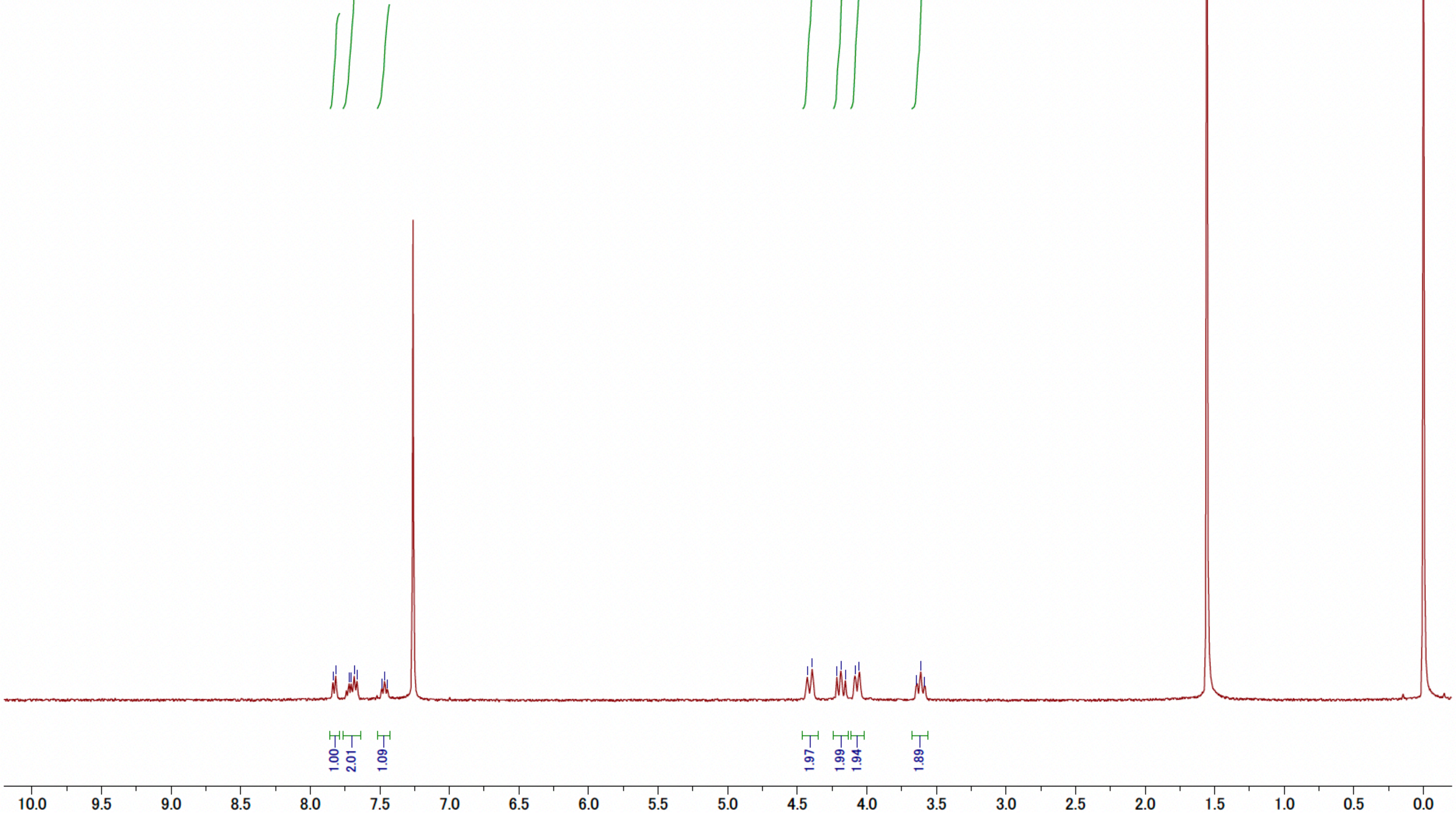

^{13}C NMR (100 MHz, CD_3CN) ; **3t**

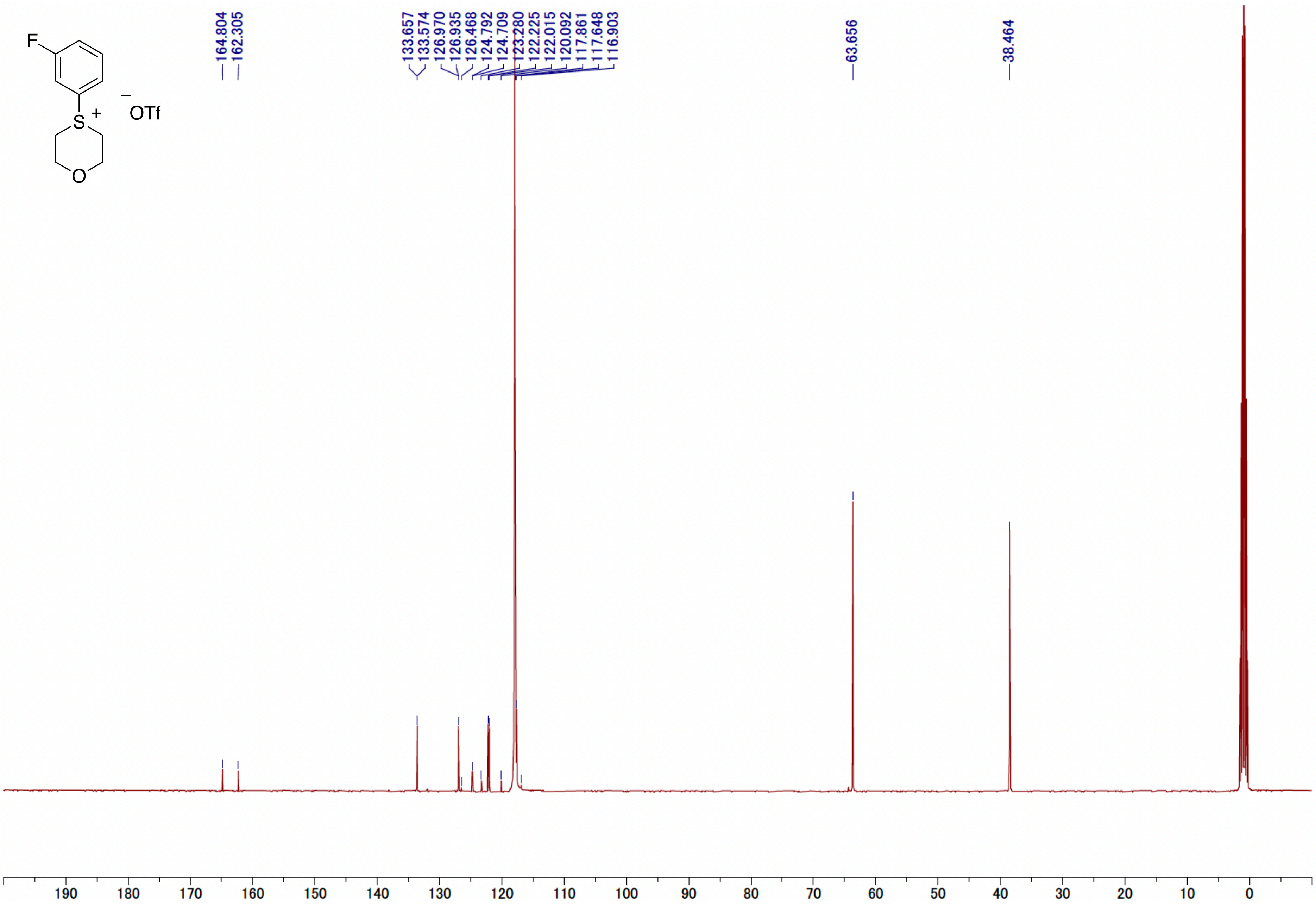

^{19}F NMR (376 MHz, CD_3CN) ; **3t**

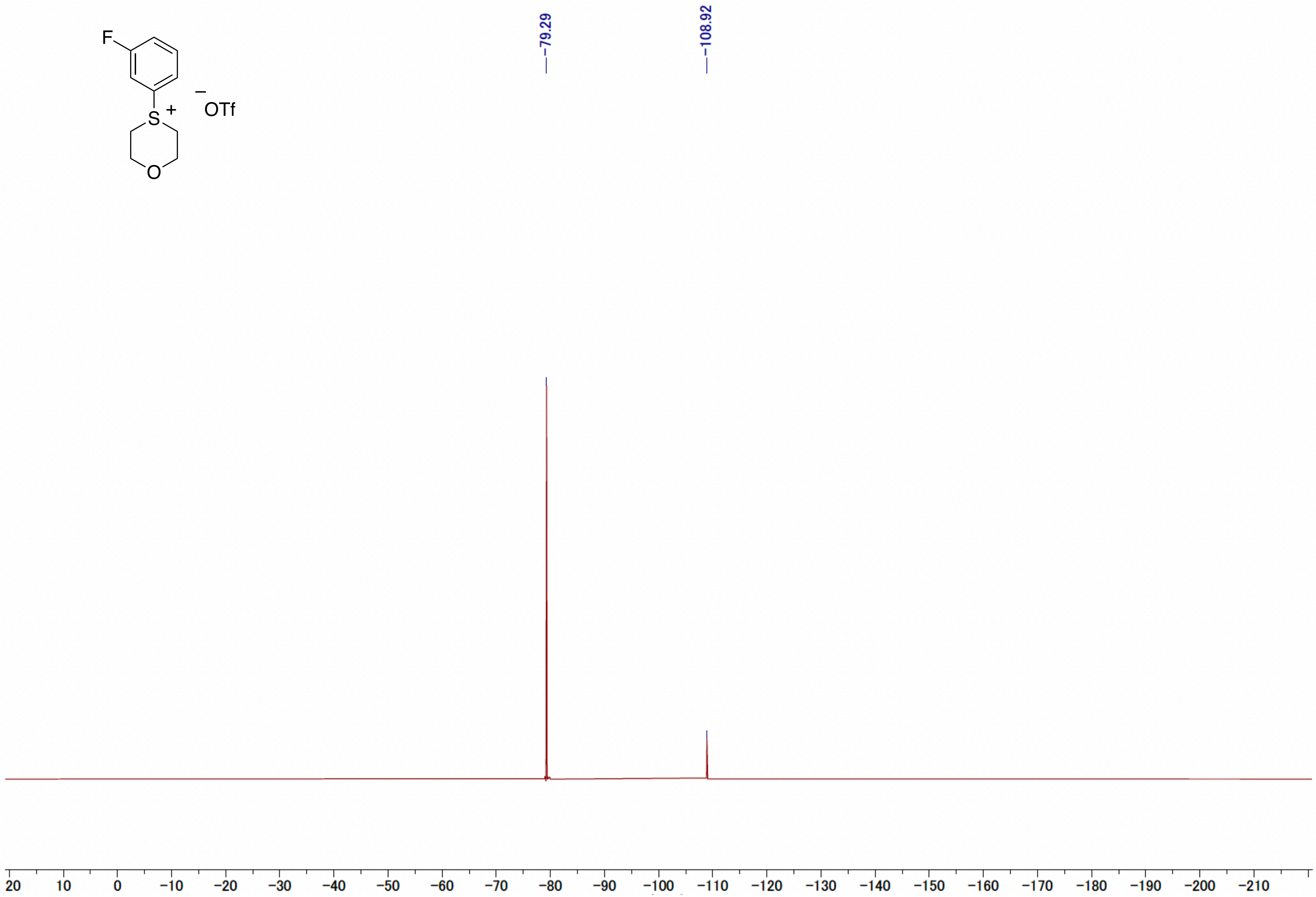

^{1}H NMR (400 MHz, CD_3CN) ; **3u**

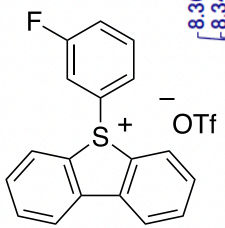

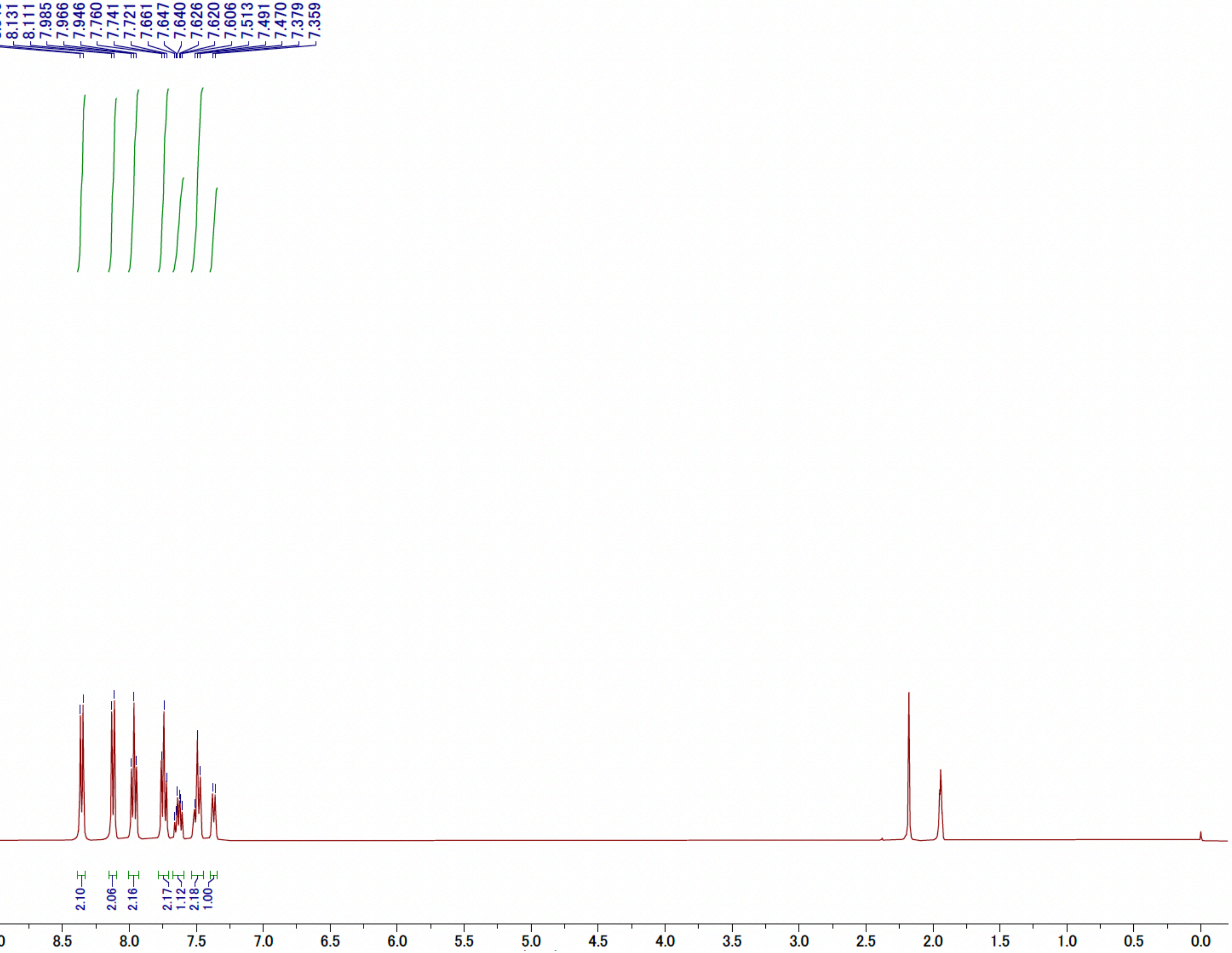

^{13}C NMR (100 MHz, CD_3CN) ; **3u**

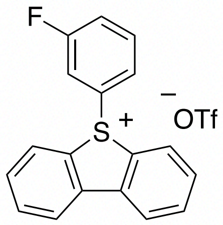

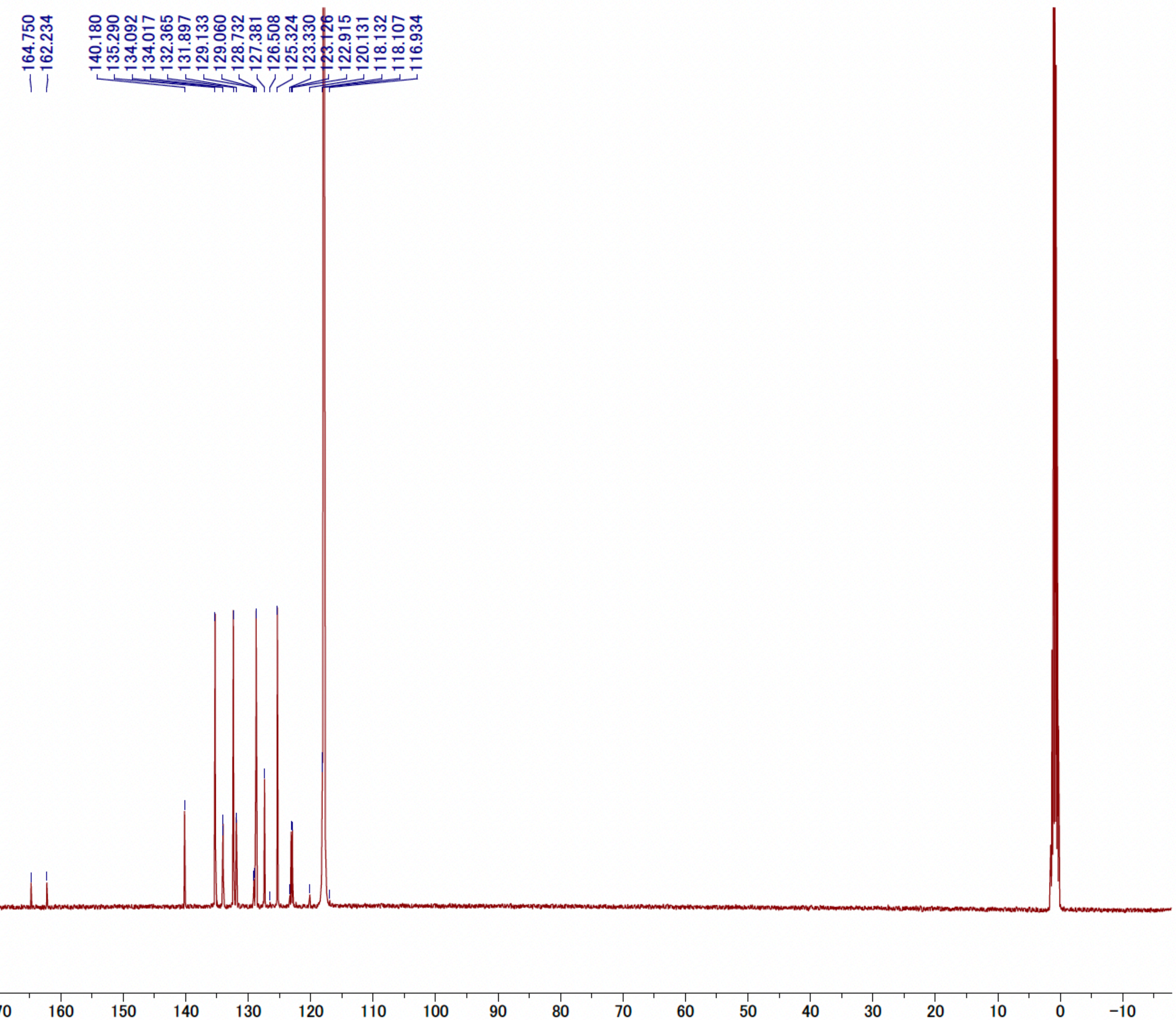

^{19}F NMR (376 MHz, CD_3CN) ; **3u**

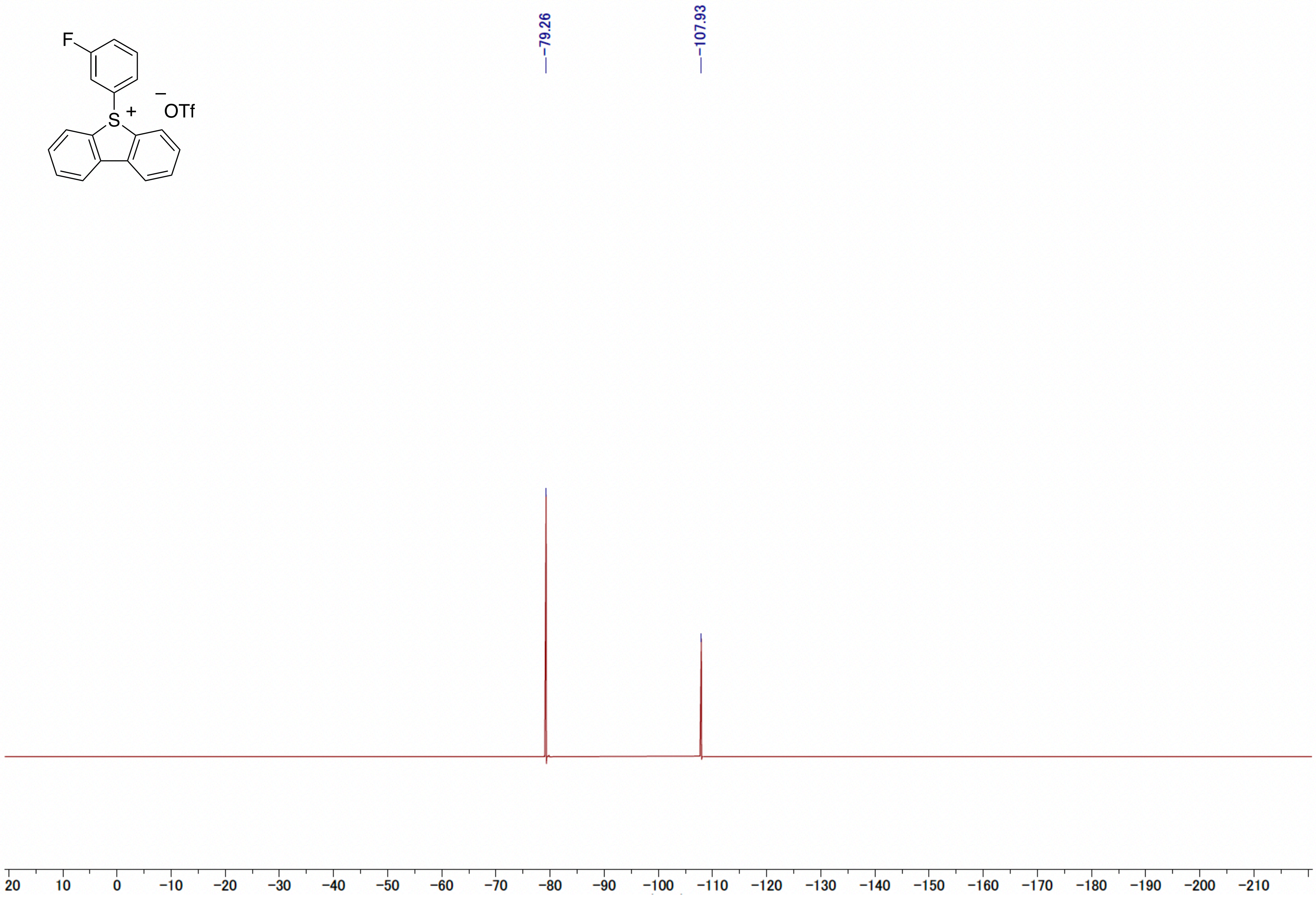

^{1}H NMR (400 MHz, $CDCl_3$) ; **3v**

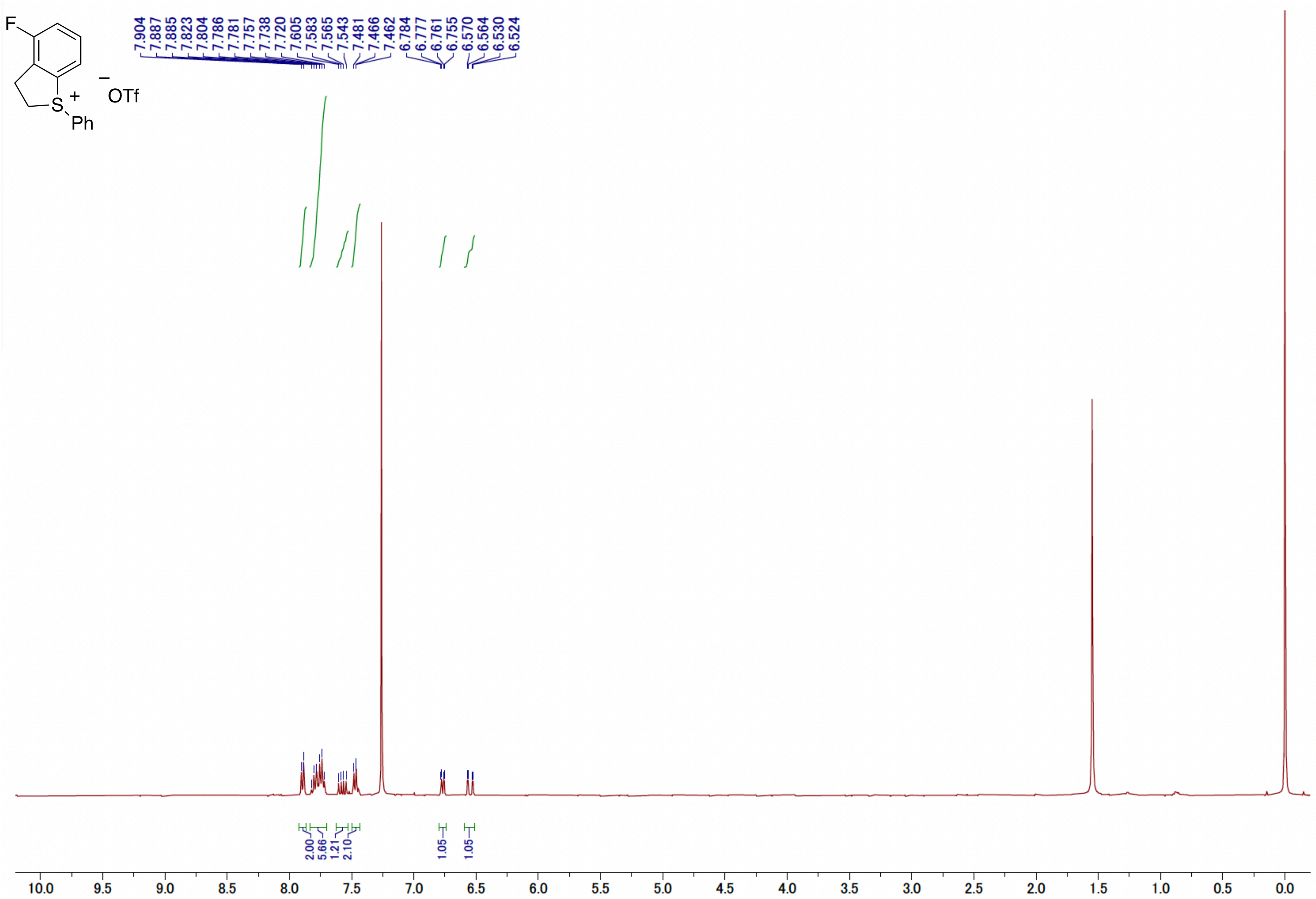

^{13}C NMR (100 MHz, $CDCl_3$) ; **3v**

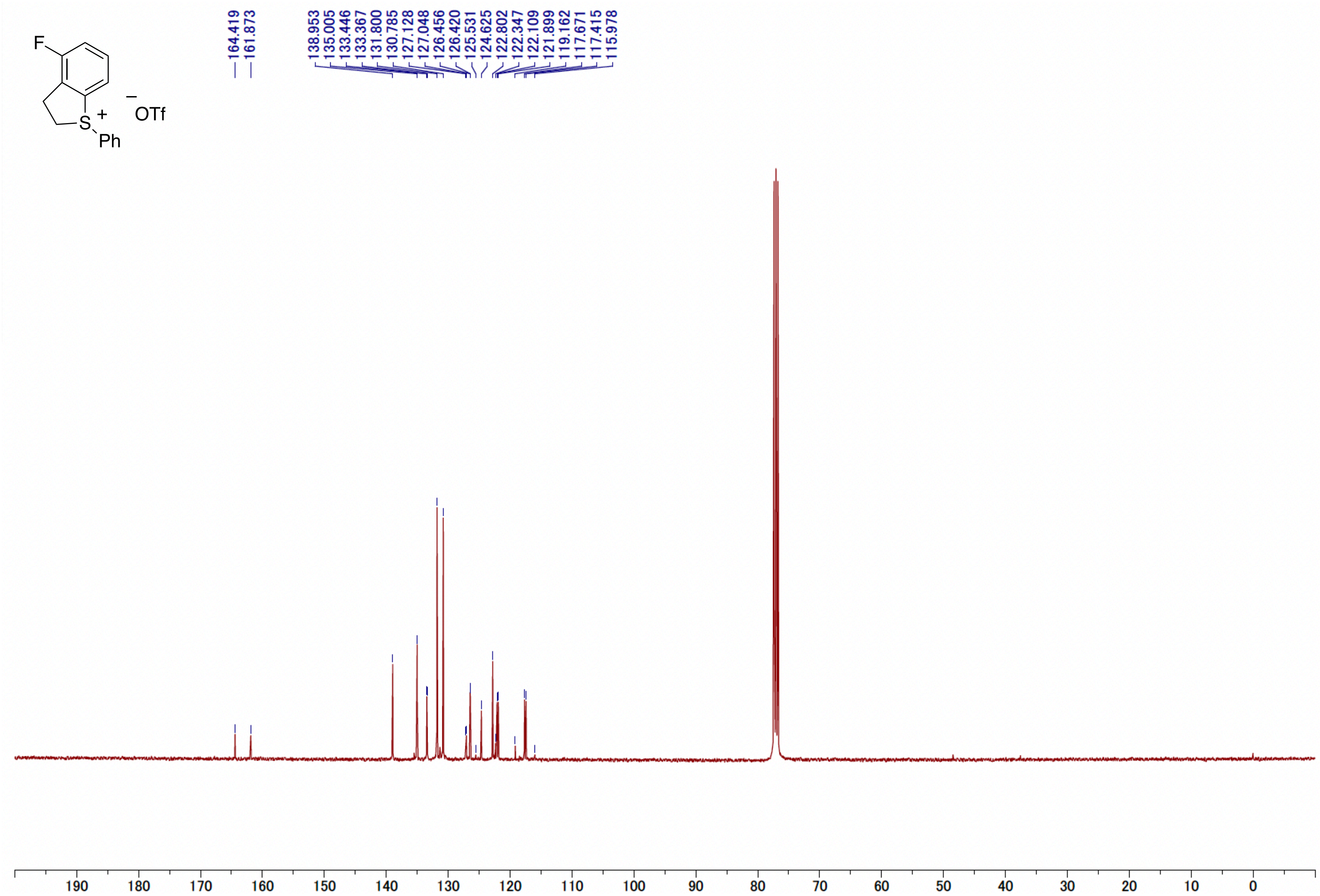

^{19}F NMR (376 MHz, $CDCl_3$) ; **3v**

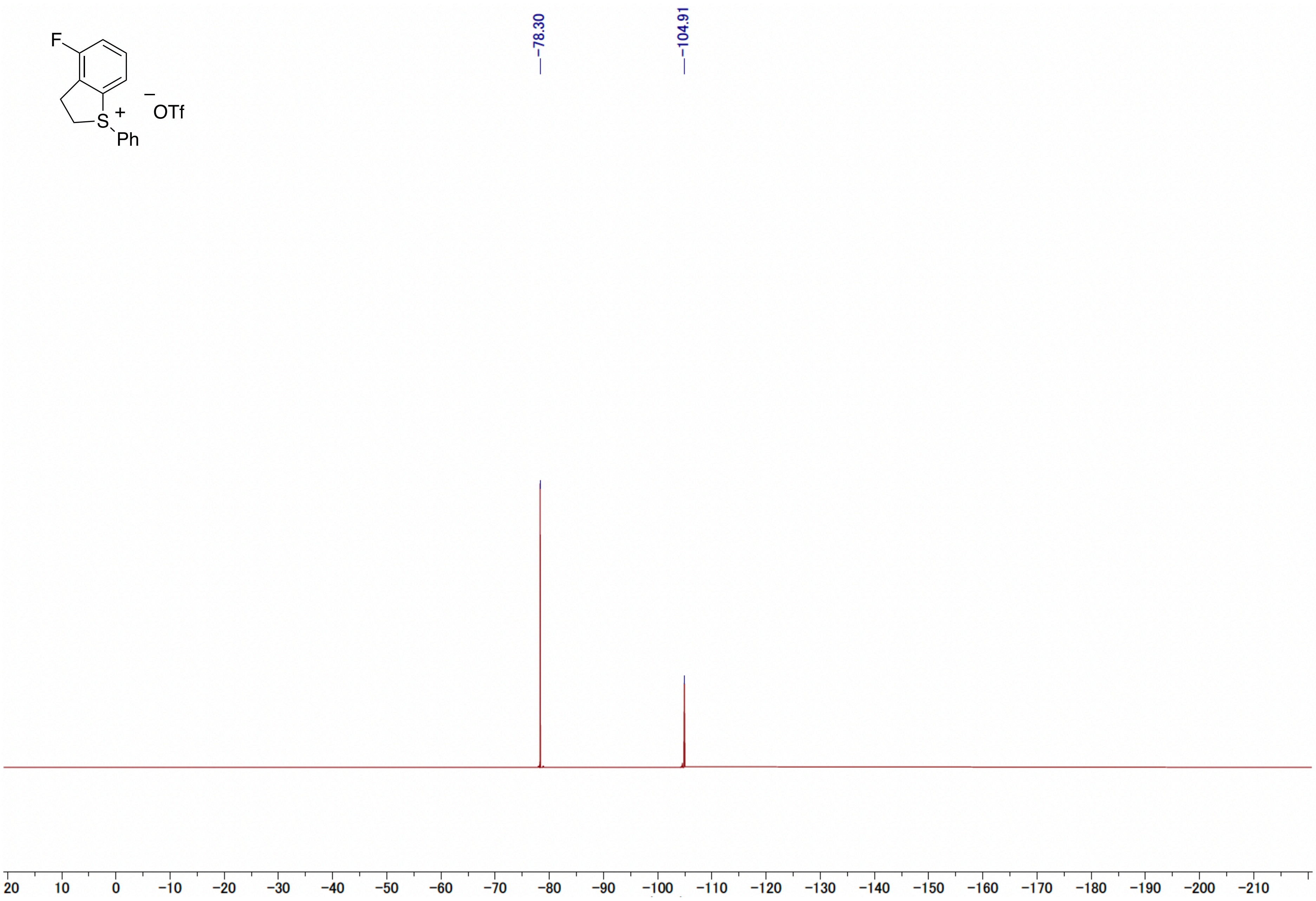

^{1}H NMR (400 MHz, $CDCl_3$) ; **3w**

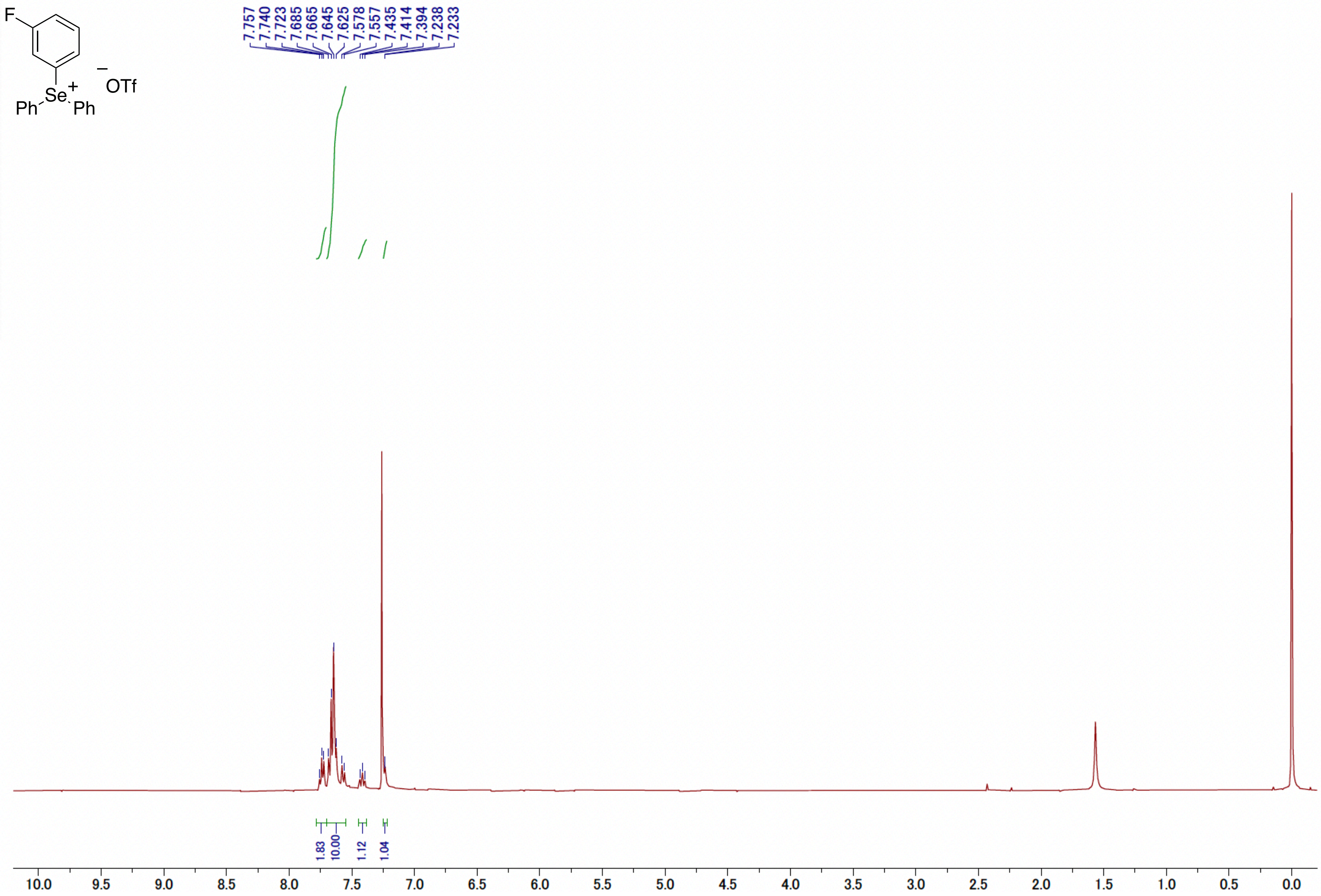

^{13}C NMR (100 MHz, CD_3CN) ; **3w**

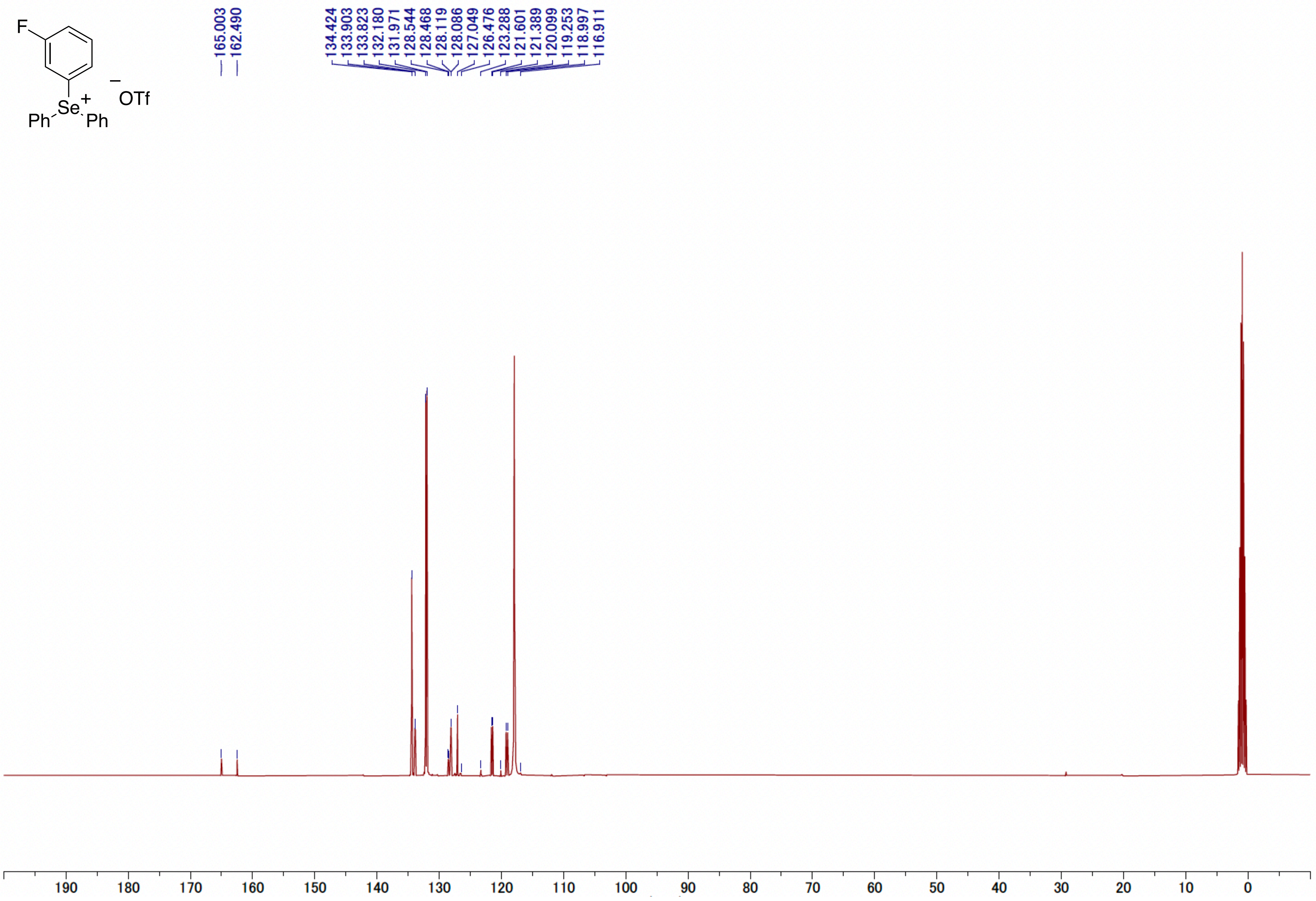

^{19}F NMR (376 MHz, CD_3CN) ; **3w**

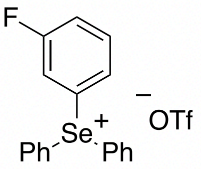

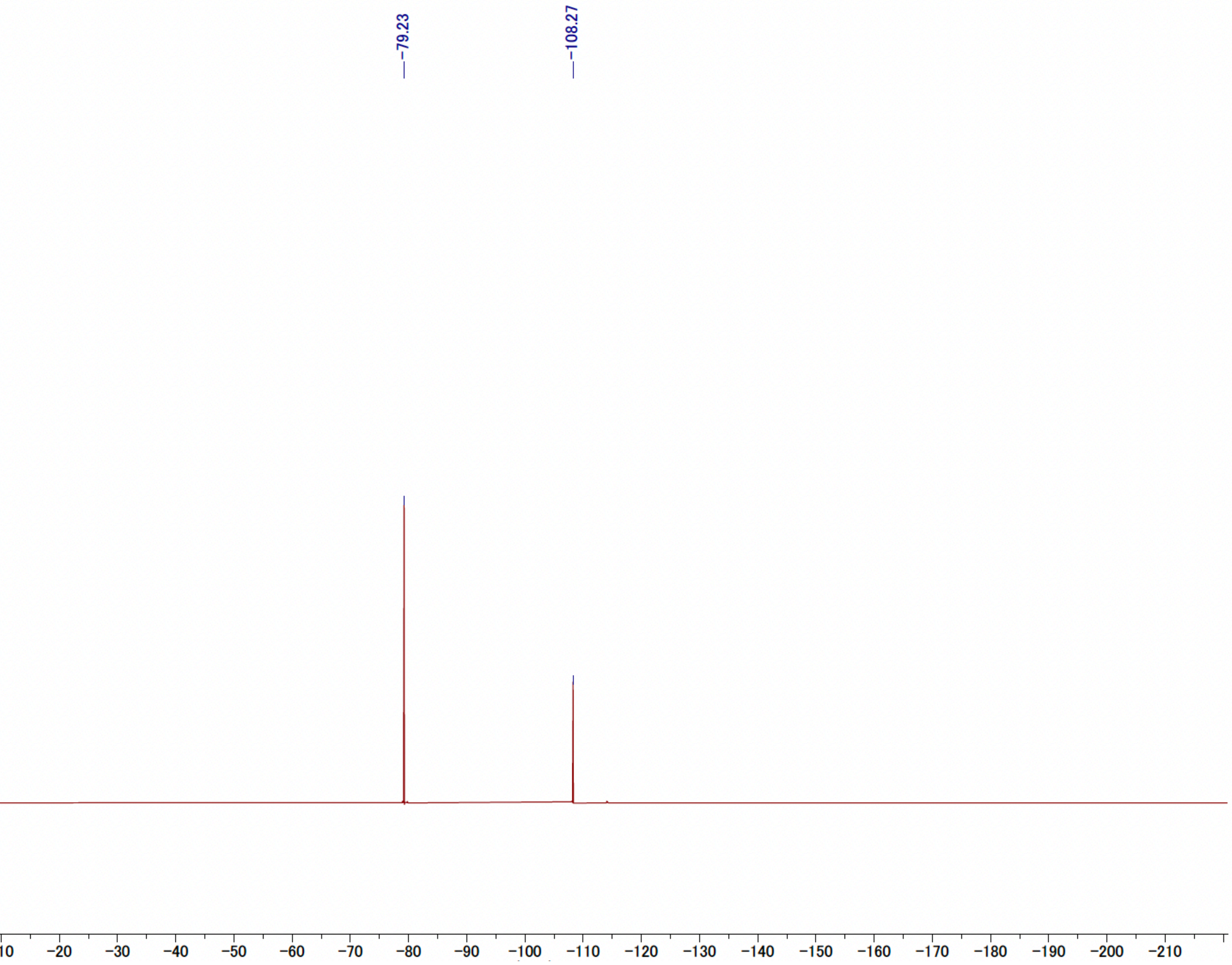

^{1}H NMR (400 MHz, $CDCl_3$) ; **4**

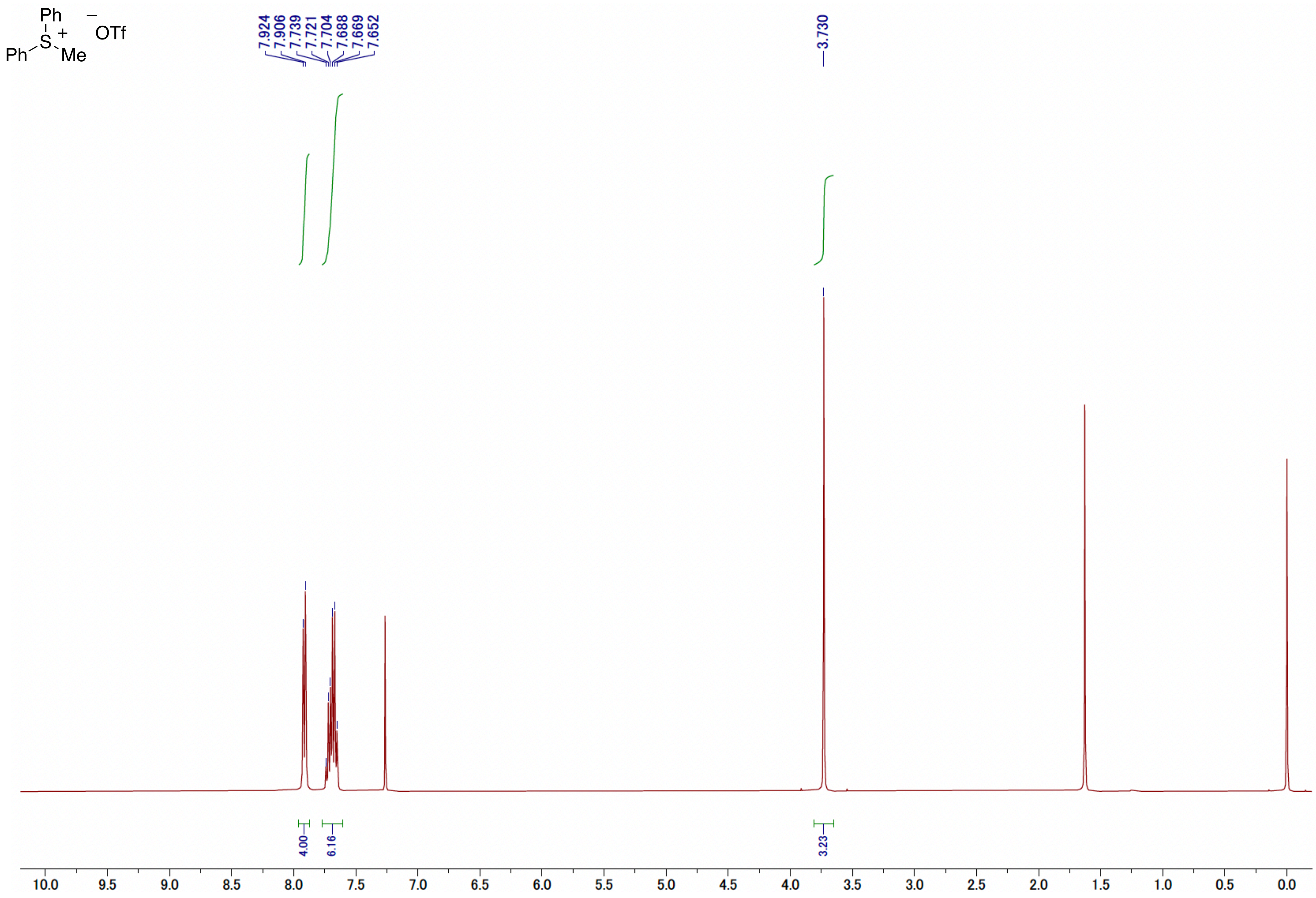

^{13}C NMR (100 MHz, CD_3CN) ; **4**

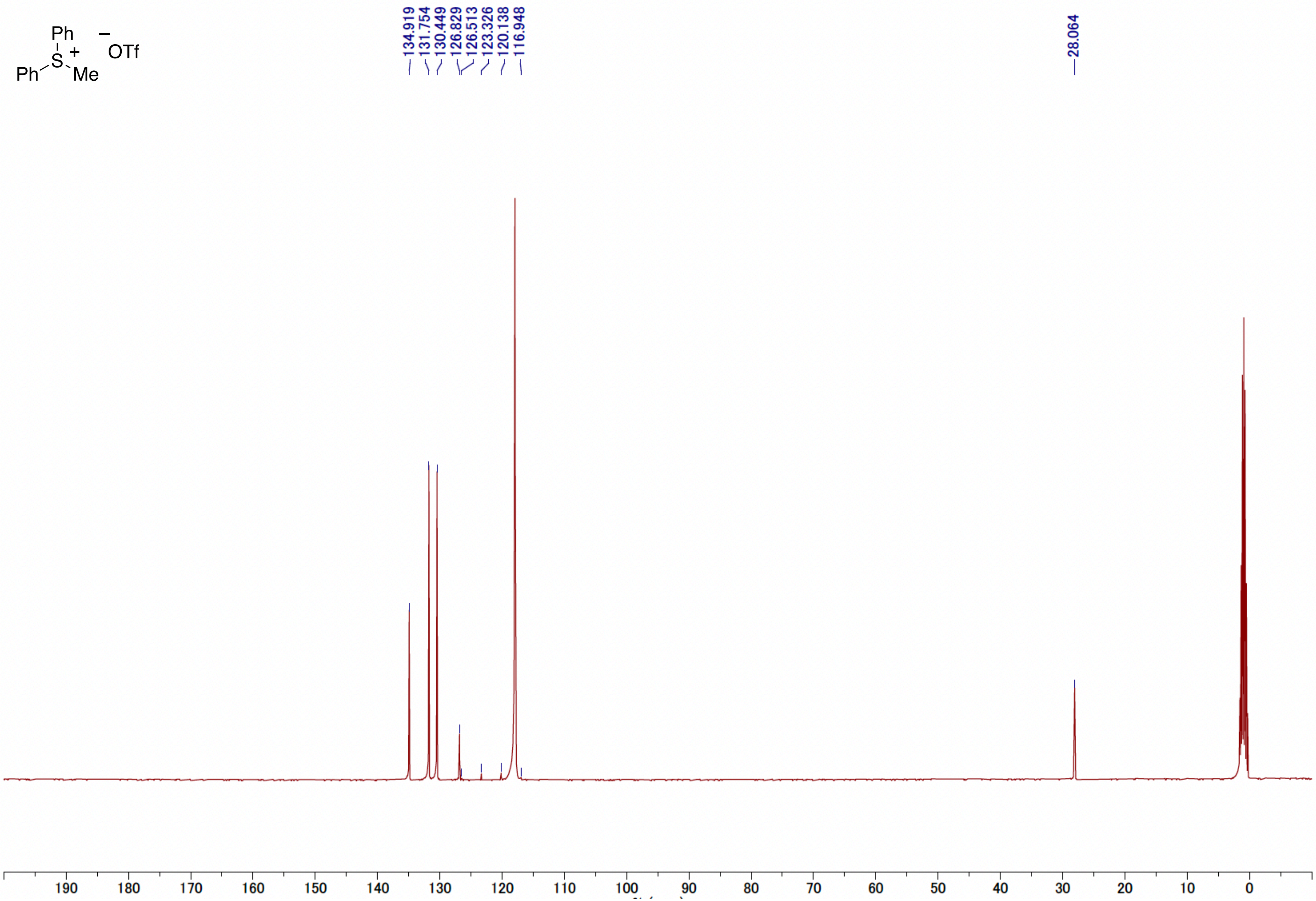

^{19}F NMR (376 MHz, CD_3CN) ; **4**

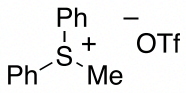

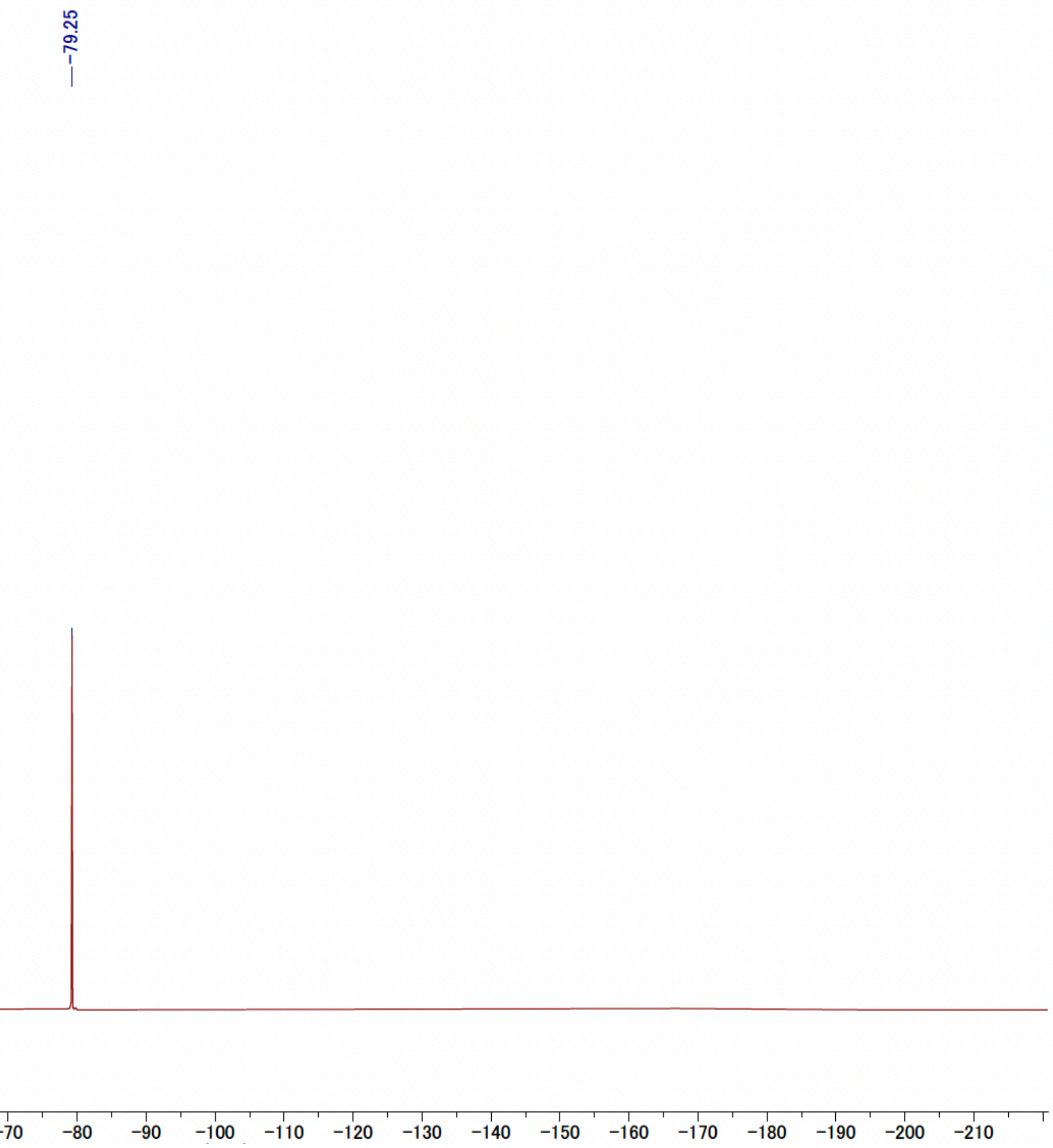

^{1}H NMR (400 MHz, $CDCl_3$) ; **5**

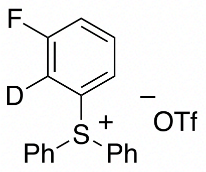

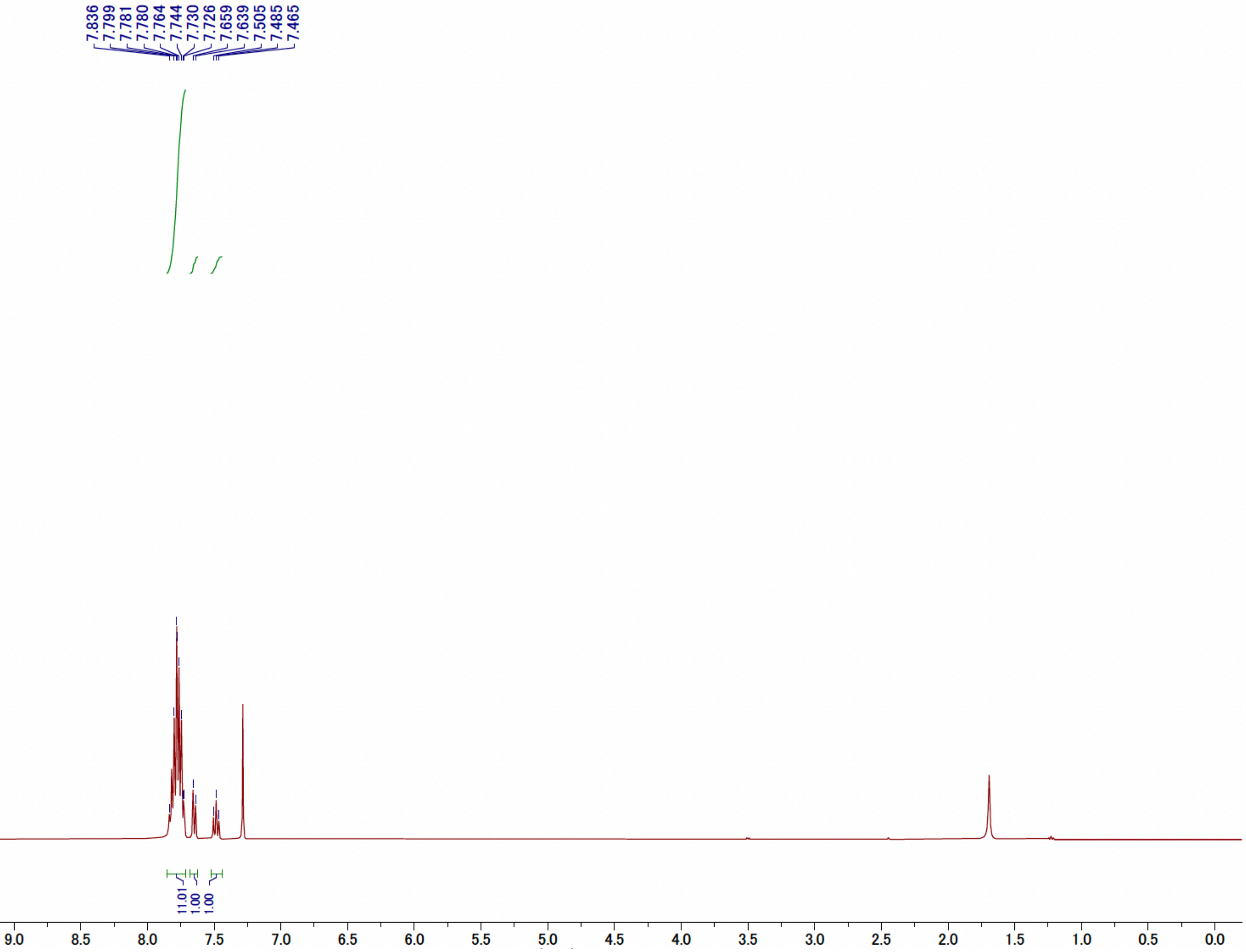

^{13}C NMR (100 MHz, CD_3CN) ; **5**

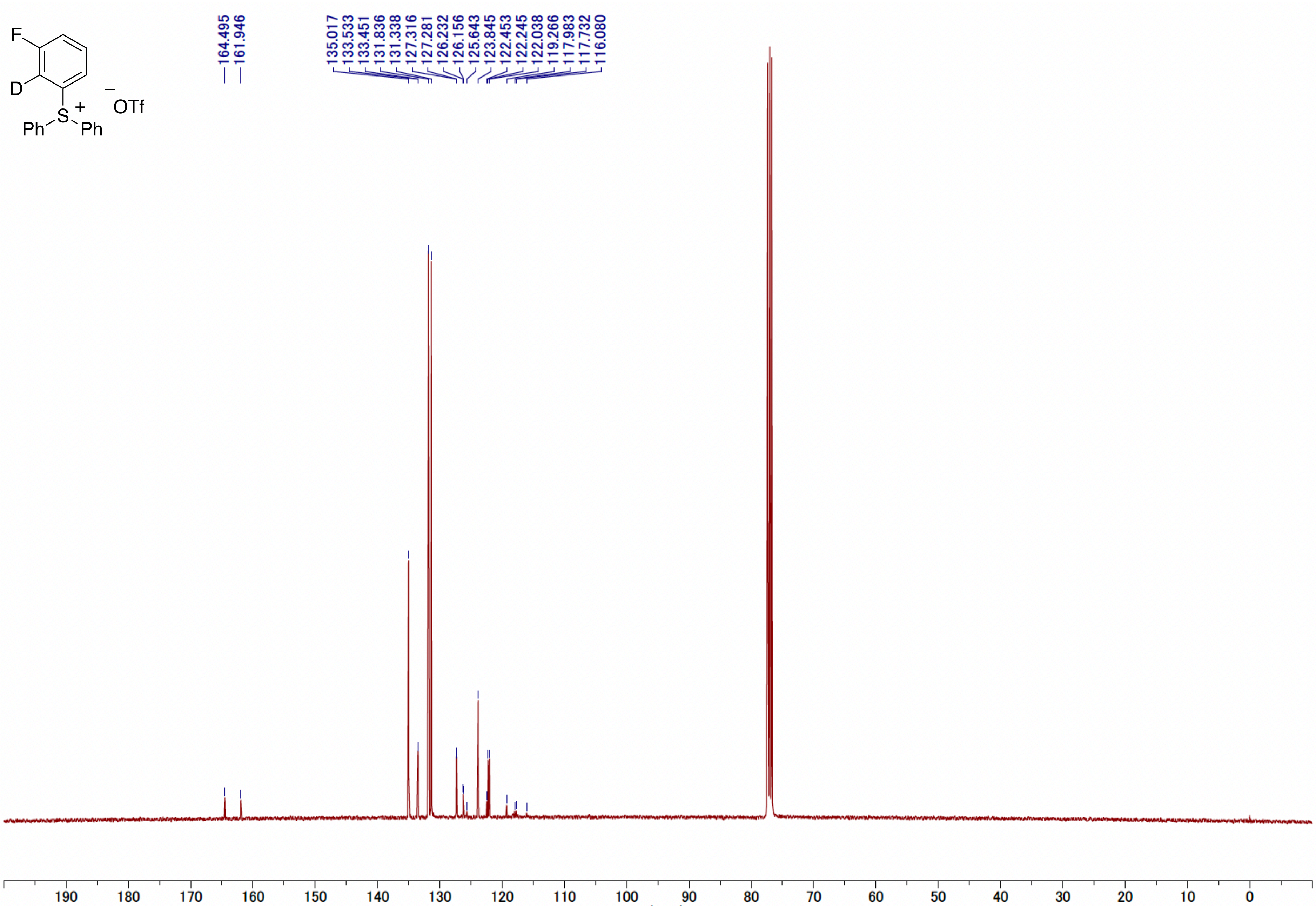

^{19}F NMR (376 MHz, $CDCl_3$) ; **5**

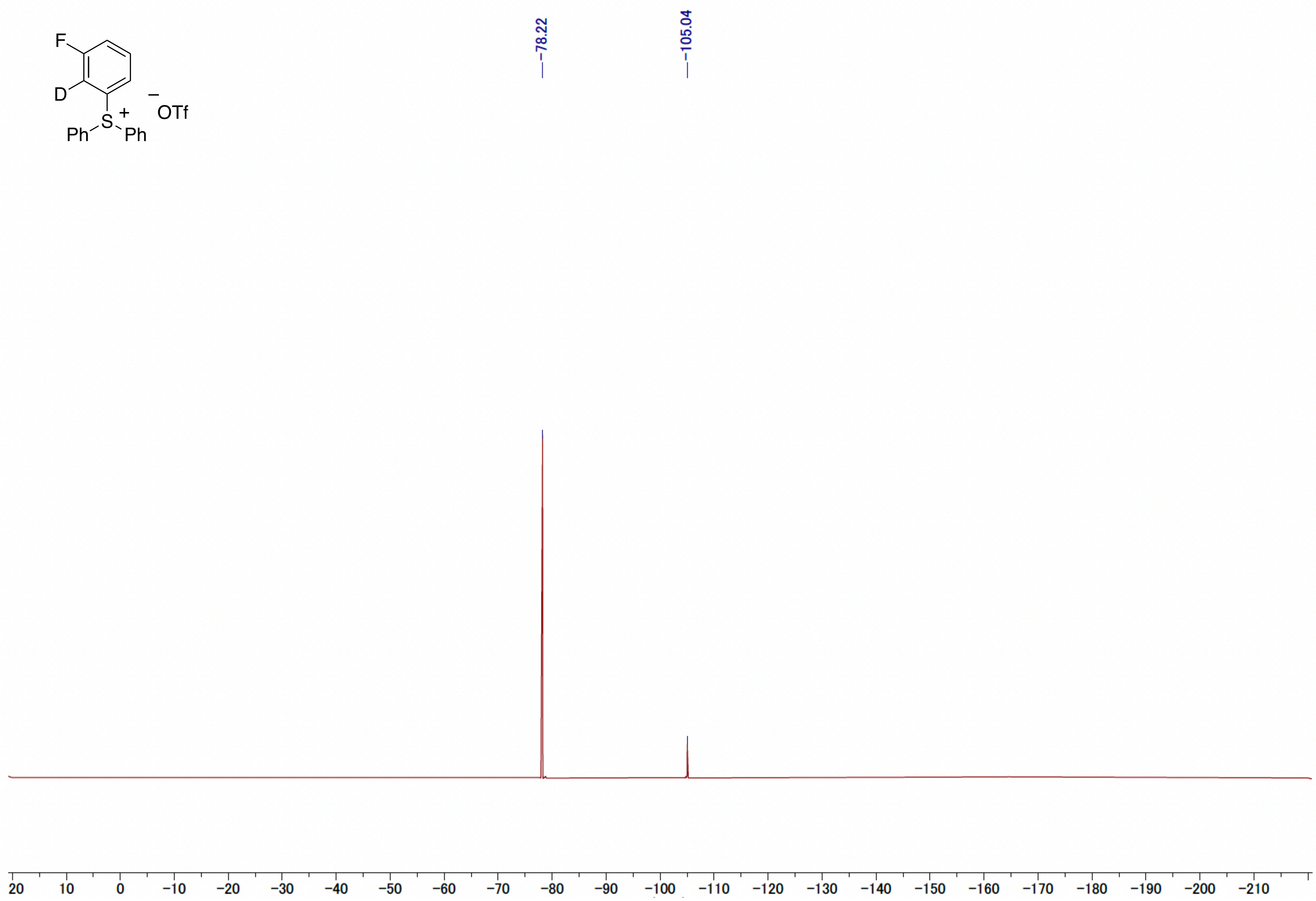

^{1}H NMR (400 MHz, $CDCl_3$) ; **7a**

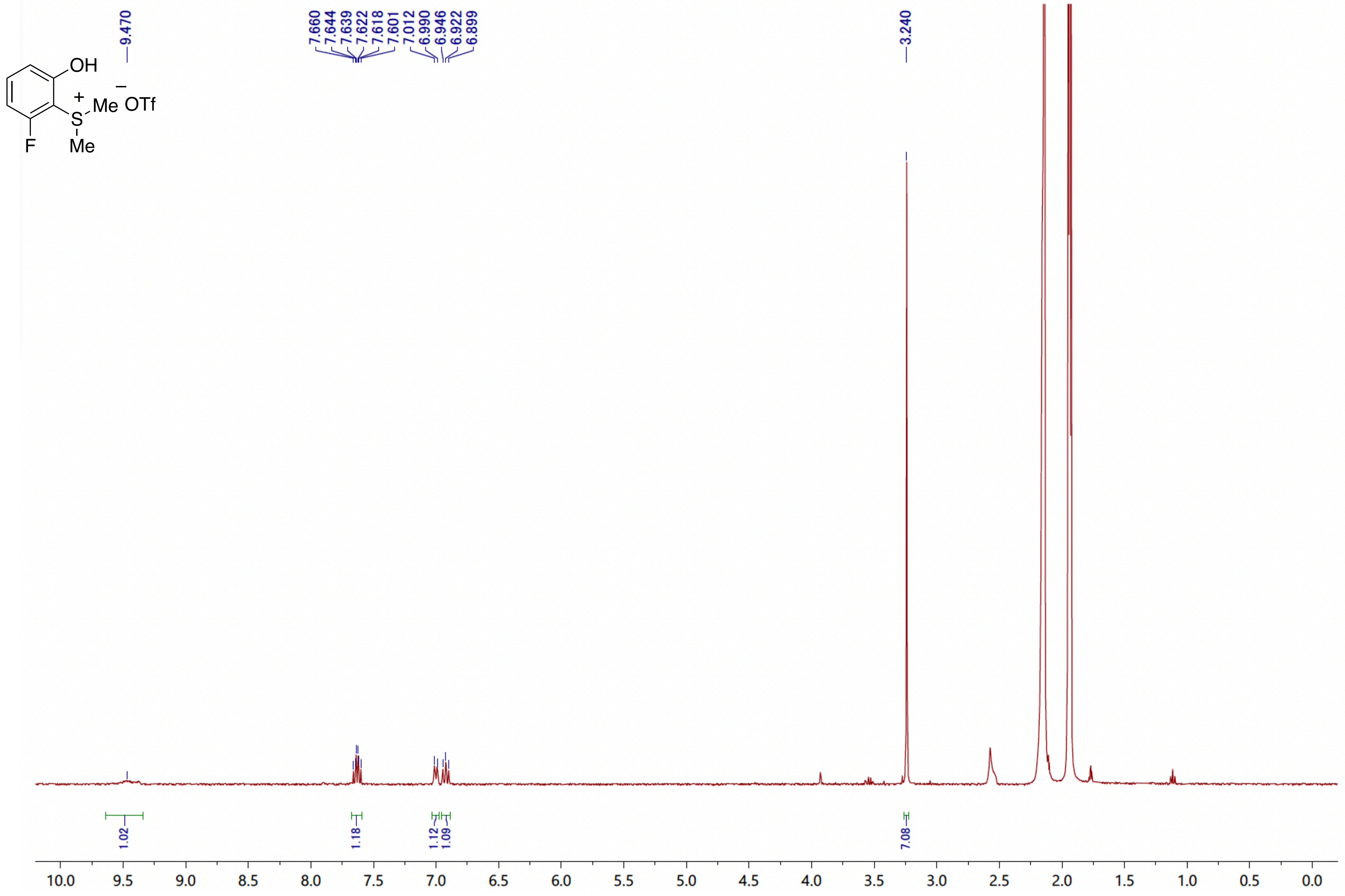

^{13}C NMR (100 MHz, CD_3CN) ; **7a**

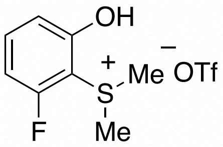

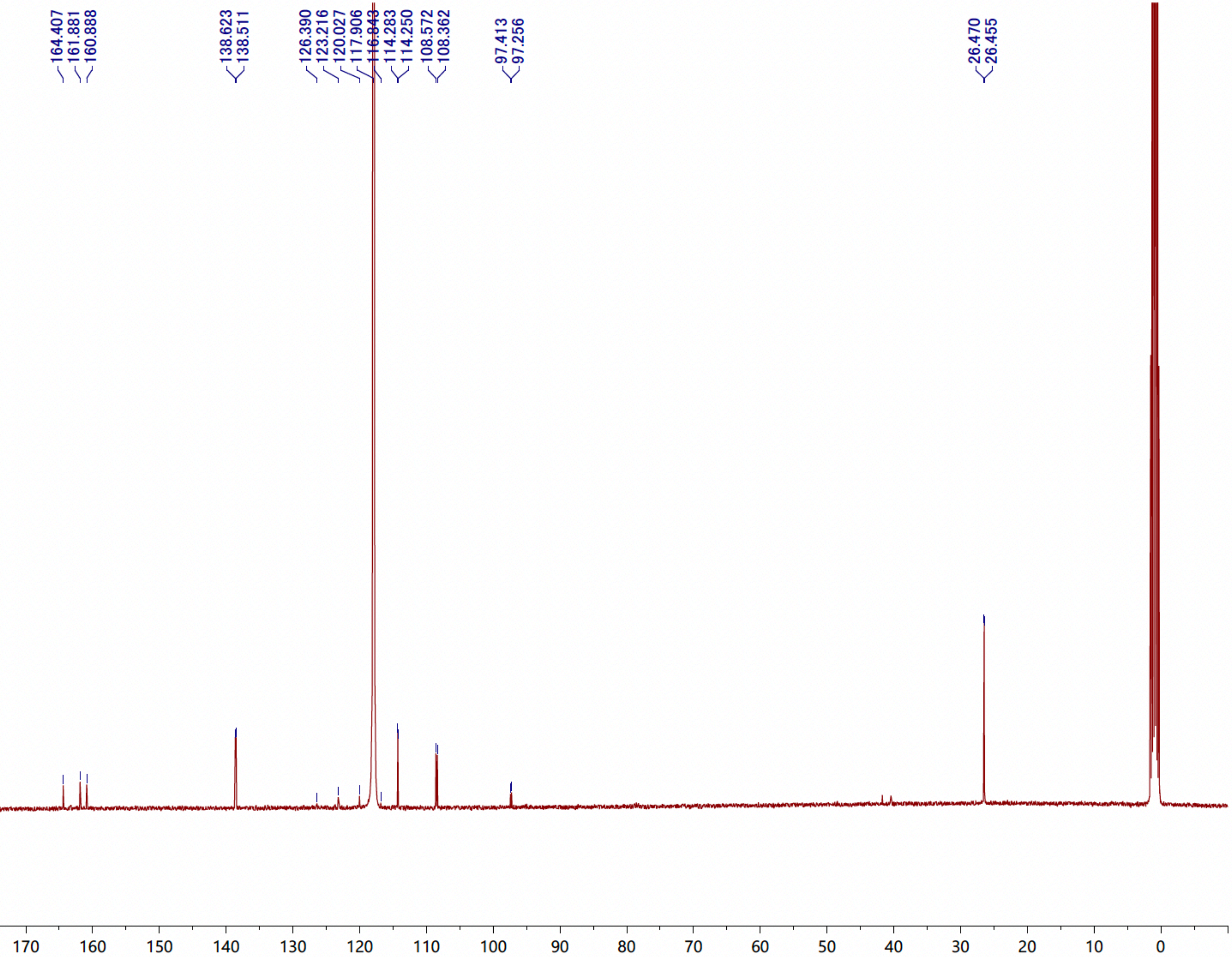

^{19}F NMR (376 MHz, $CDCl_3$) ; **7a**

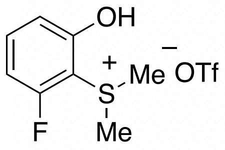

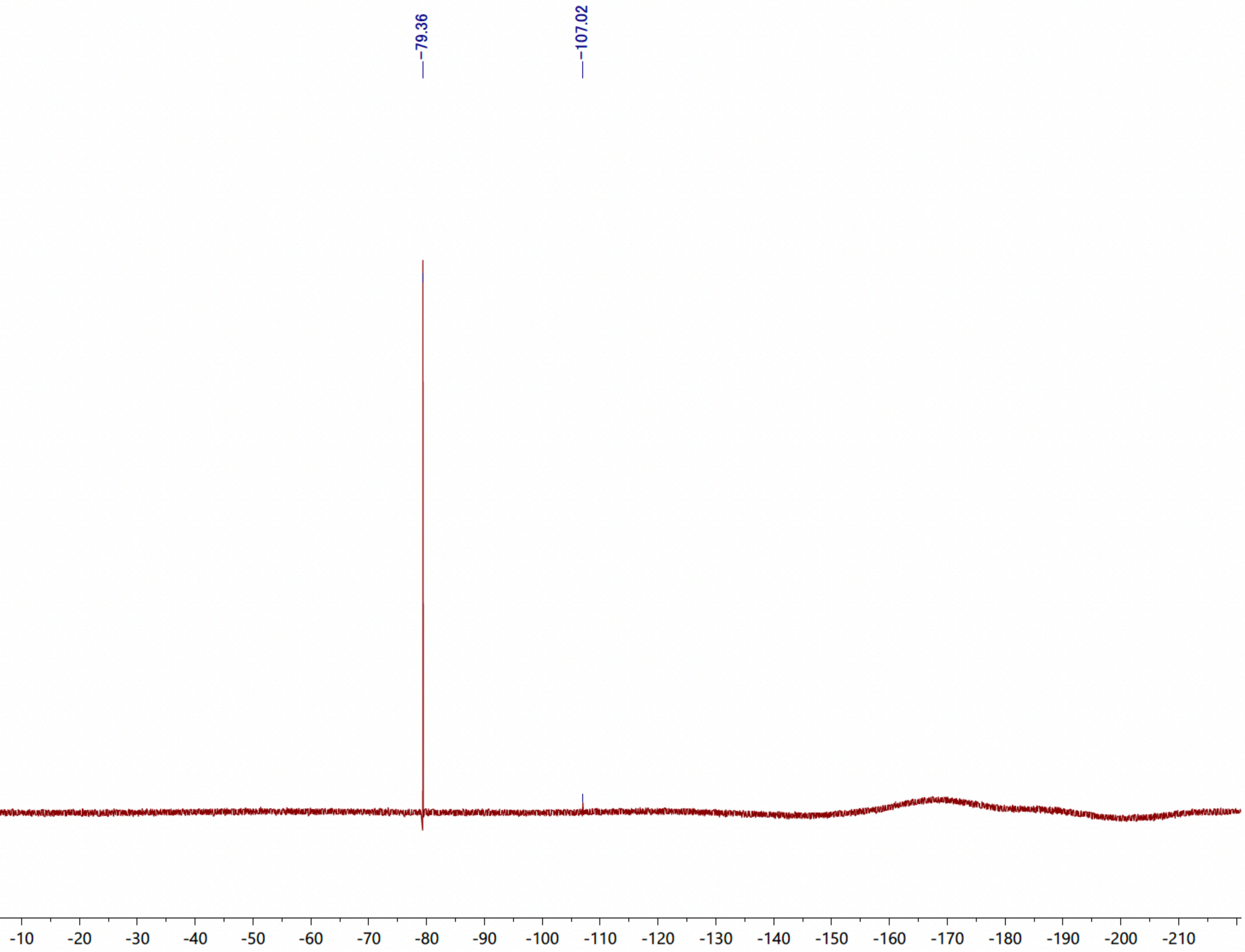

^{1}H NMR (400 MHz, $CDCl_3$) ; **7b**

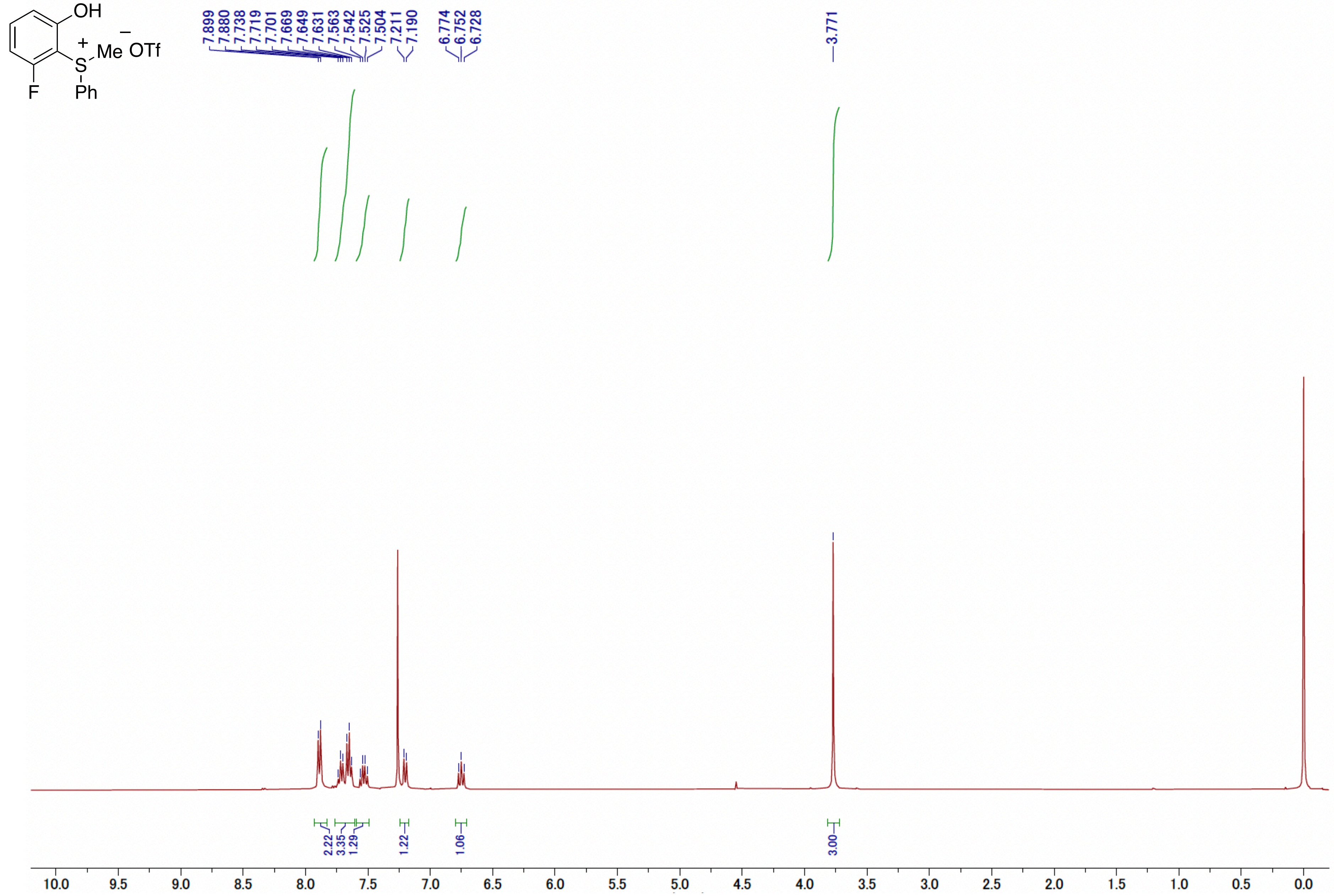

^{13}C NMR (100 MHz, CD_3CN) ; **7b**

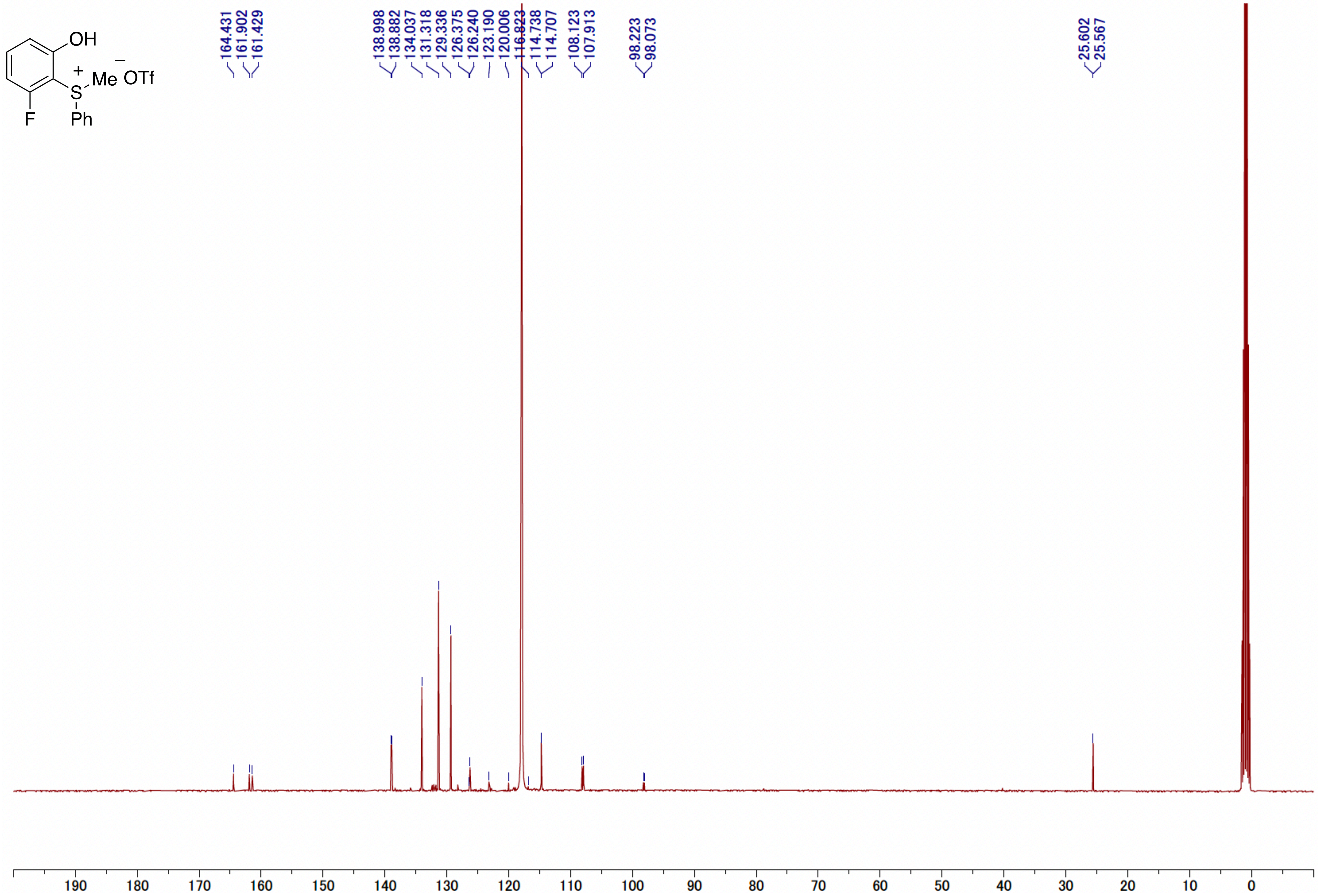

^{19}F NMR (376 MHz, CD_3CN) ; **7b**

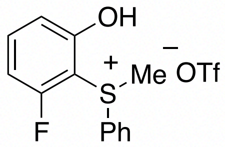

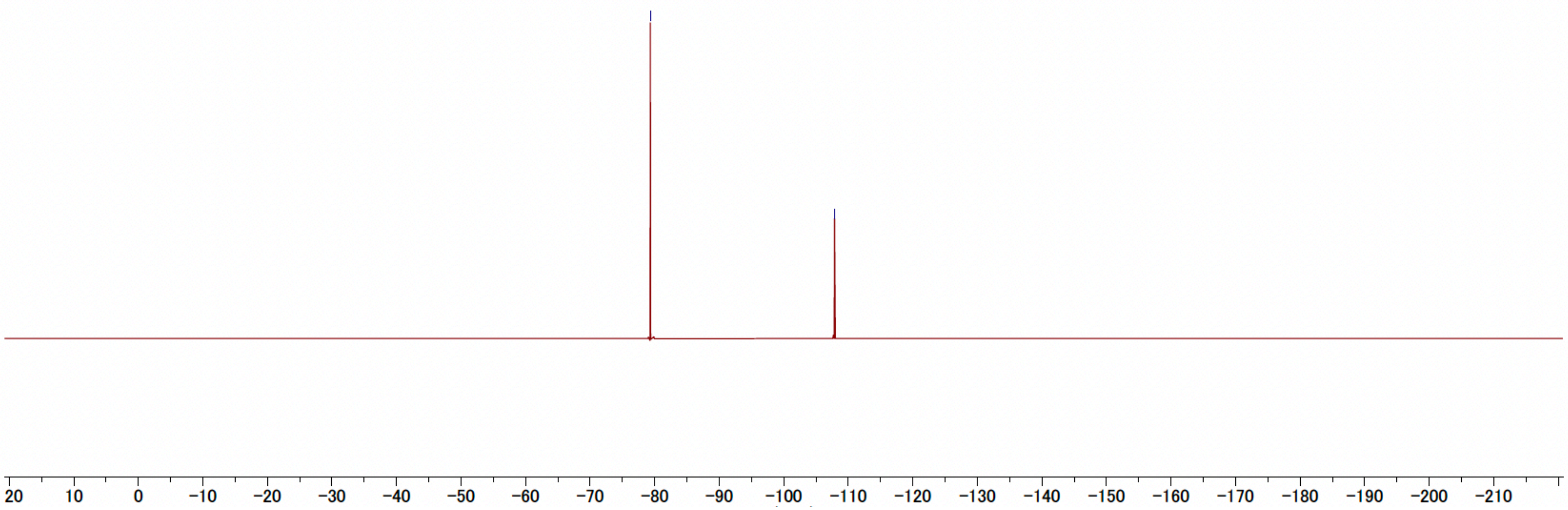

^{1}H NMR (400 MHz, $CDCl_3$) ; **11**

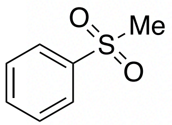

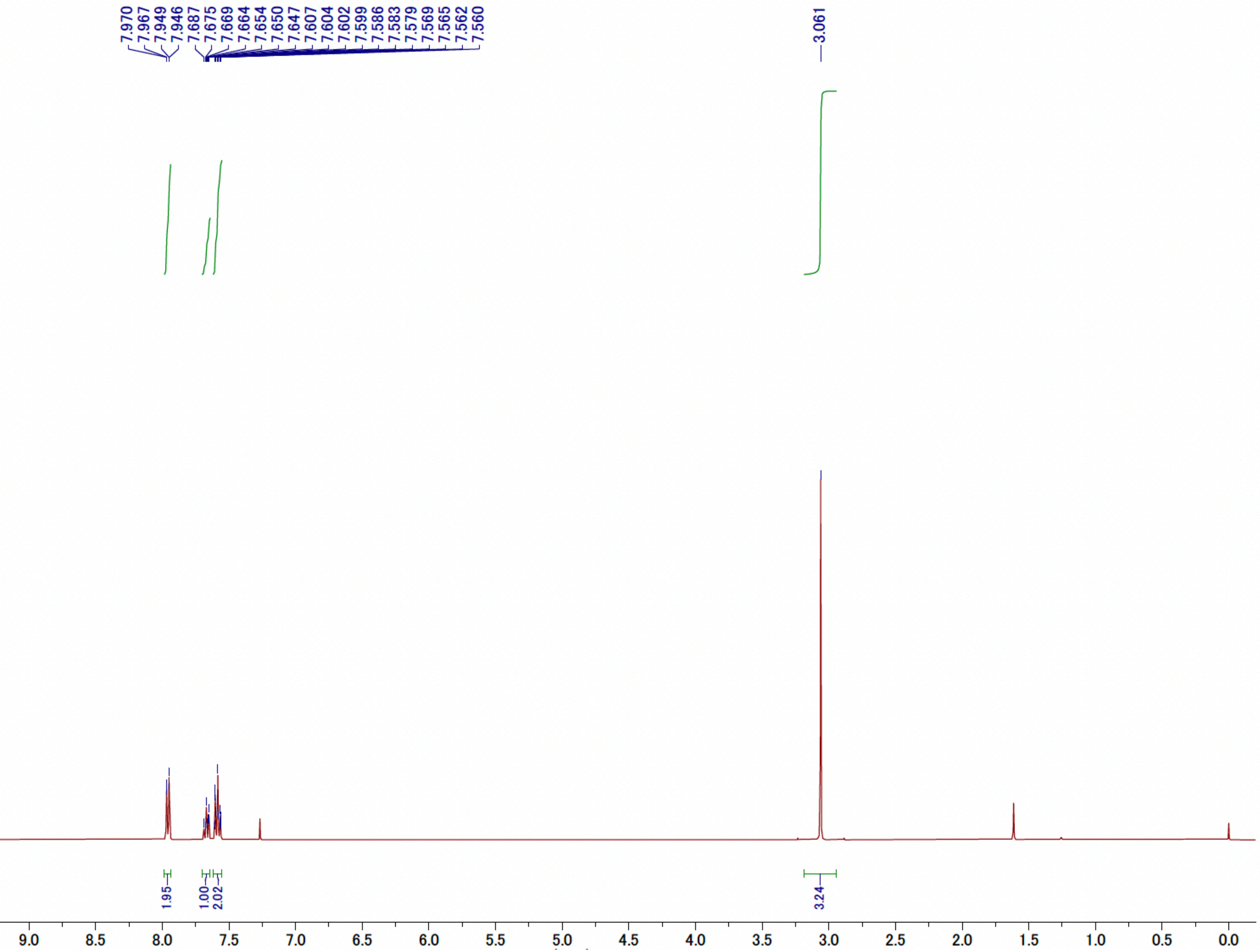

^{1}H NMR (400 MHz, $CDCl_3$) ; **12**

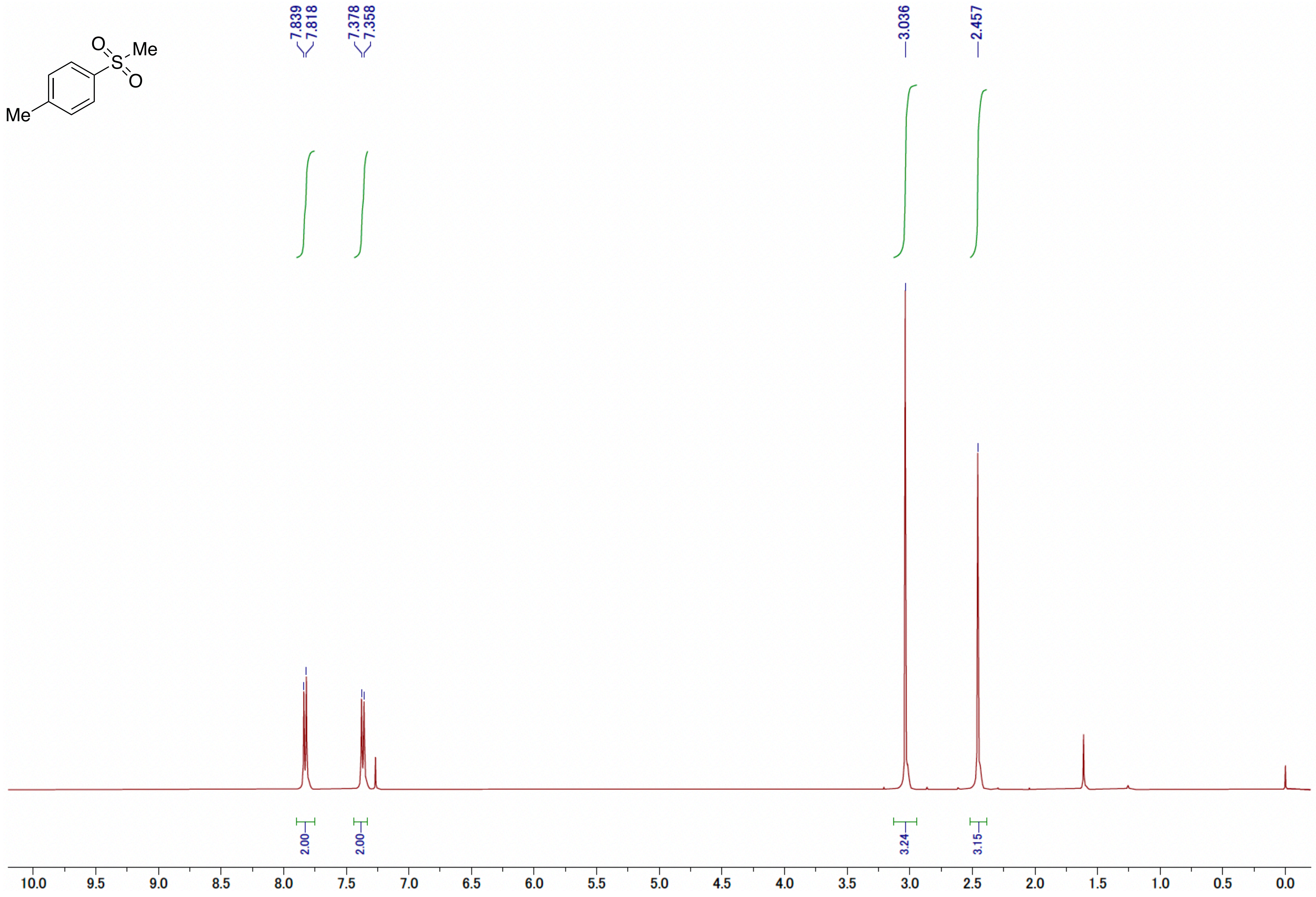

^{13}C NMR (100 MHz, $CDCl_3$) ; **12**

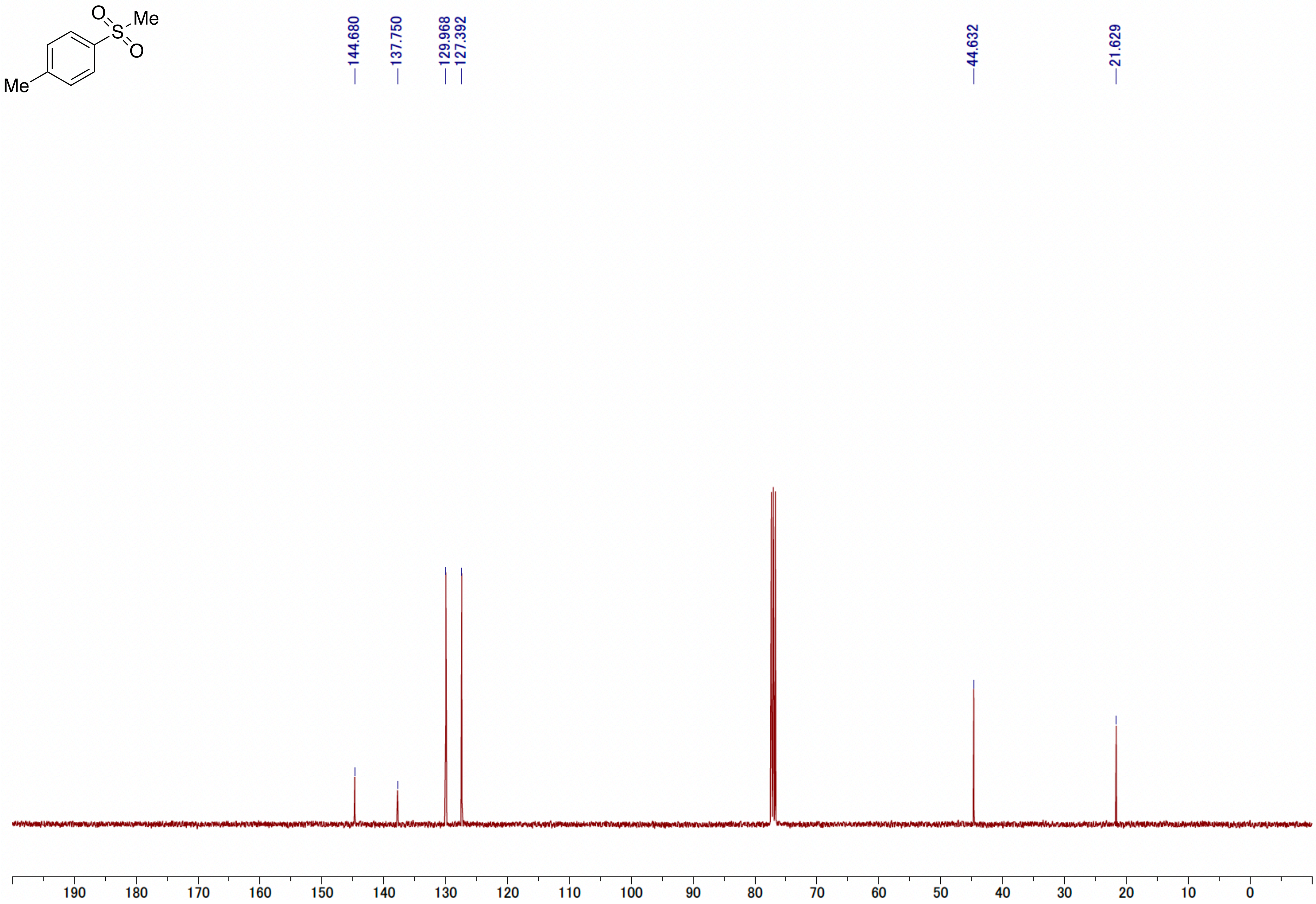

^{1}H NMR (400 MHz, $CDCl_3$) ; **13**

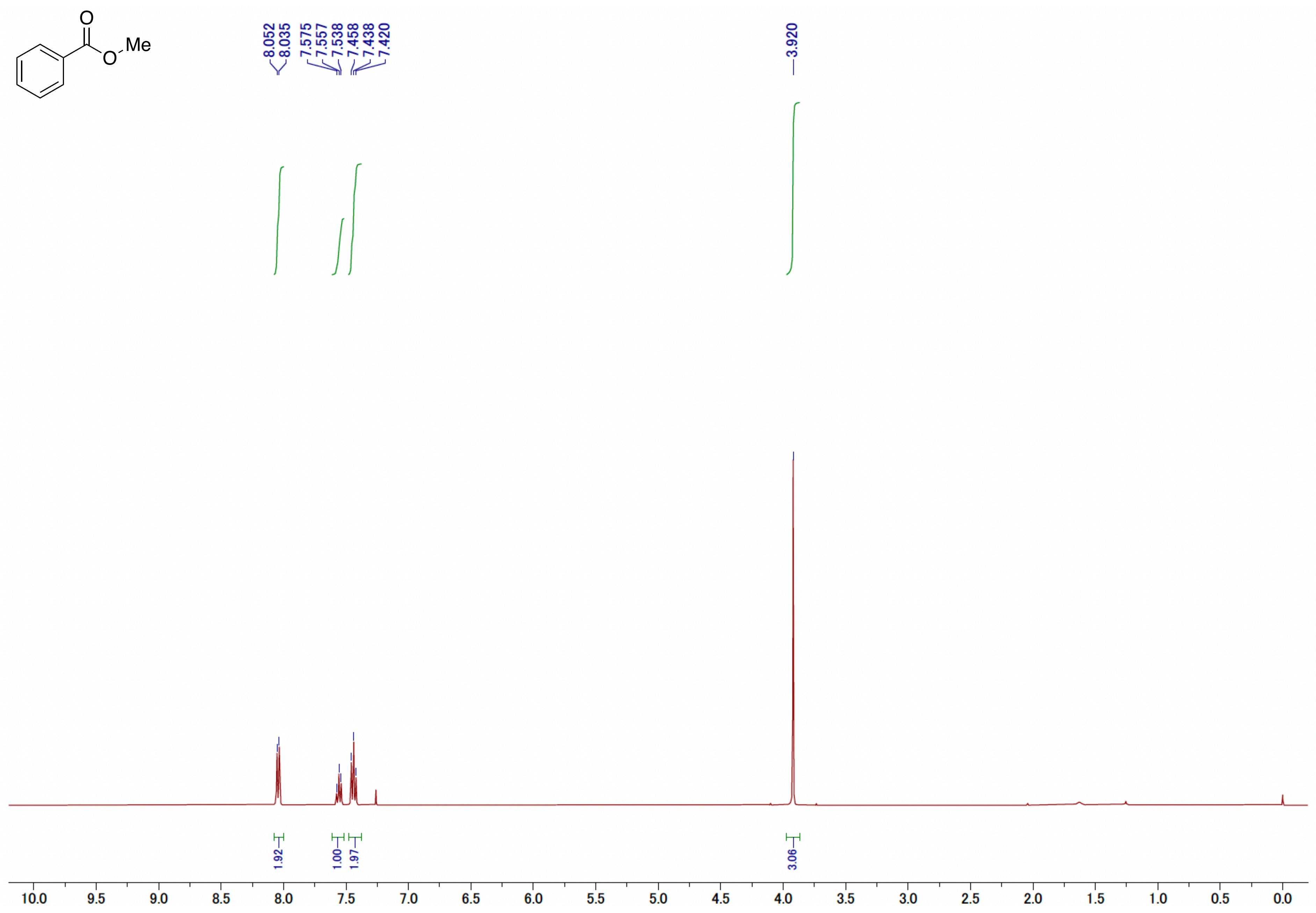

^{13}C NMR (100 MHz, $CDCl_3$) ; **13**

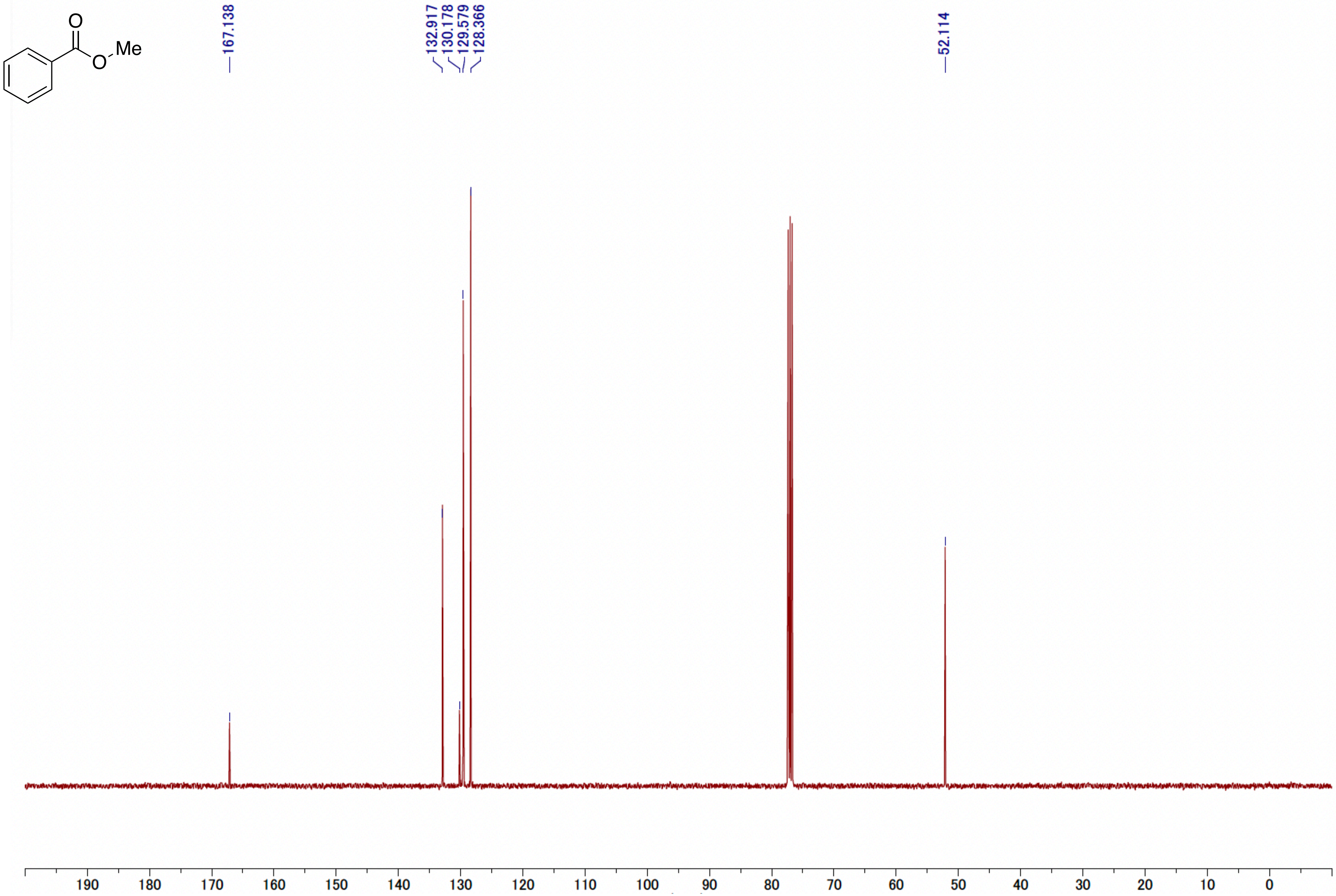

Supplement: Supplementary file 1 — ol4c00197_si_001.pdf [file ol4c00197_si_001.pdf]
